# Rational Design of Simple Organocatalysts for the HSiCl_3_ Enantioselective Reduction of (E)-*N*-(1-Phenylethylidene)aniline

**DOI:** 10.3390/molecules26226963

**Published:** 2021-11-18

**Authors:** María Maciá, Raúl Porcar, Vicente Martí-Centelles, Eduardo García-Verdugo, Maria Isabel Burguete, Santiago V. Luis

**Affiliations:** 1 Department of Inorganic and Organic Chemistry, Jaume I University, Av. Vicent Sos Baynat s/n, 12071 Castellón, Spain; maciam@uji.es (M.M.); rporcar@ccia.uned.es (R.P.); martiv@uji.es (V.M.-C.); burguete@uji.es (M.I.B.); 2 Department of Organic and Bio-Organic Chemistry, Faculty of Science, UNED—Universidad Nacional de Educación a Distancia, Avenida de Esparta s/n, 28232 Las Rozas-Madrid, Spain; 3 Instituto Interuniversitario de Investigación de Reconocimiento Molecular y Desarrollo Tecnológico (IDM), Universitat Politècnica de València, Universitat de València, 46022 Valencia, Spain

**Keywords:** organocatalysis, asymmetric catalysis, trichlorosilane, imine reduction, mechanistic studies

## Abstract

Prolinamides are well-known organocatalysts for the HSiCl_3_ reduction of imines; however, custom design of catalysts is based on trial-and-error experiments. In this work, we have used a combination of computational calculations and experimental work, including kinetic analyses, to properly understand this process and to design optimized catalysts for the benchmark (E)-*N*-(1-phenylethylidene)aniline. The best results have been obtained with the amide derived from 4-methoxyaniline and the *N*-pivaloyl protected proline, for which the catalyzed process is almost 600 times faster than the uncatalyzed one. Mechanistic studies reveal that the formation of the component supramolecular complex catalyst-HSiCl_3_-substrate, involving hydrogen bonding breaking and costly conformational changes in the prolinamide, is an important step in the overall process.

## 1. Introduction

Chiral amines are an important class of organic compounds widely used as building blocks in synthetic organic chemistry [1,2]. A large number of chiral active pharmaceutical ingredients, about one-third of the total API market, and agrochemical substances are amines or contain functional groups derived from amines. These include (*S*)-Rivastigmine (Alzheimer’s and Parkinson’s diseases), NPS R-568 (hyperparathyroidism treatment), and (*R*)-Fendiline (calcium channel blocker) [3]. In this context, the asymmetric transfer hydrogenation of imines represents a simple and attractive approach to target the synthesis of enantiopure amines, and a variety of catalytic methodologies have been developed for this purpose [4,5,6,7,8,9,10,11,12]. The use of trichlorosilane and related reagents for this purpose has attracted much attention in recent years, having shown their capacity for the transfer hydrogenation of a variety of groups, such imines [4,5,6,7,8,9,10,11,12], enones [13,14], nitro groups [15,16], and *N*-heteroarenes [17,18], in the synthesis of substituted hydrazines [19], and in the reduction of CO_2_ in the presence of amines [20,21,22,23]. Thus, the organocatalytic reduction of ketimines with trichlorosilane provides an efficient methodology for the asymmetric preparation of amines [24,25,26]. Different organocatalysts have been reported and evaluated against a series of benchmark ketimines [27,28,29,30,31,32,33] and related substrates, in particular enamines [34,35,36,37,38,39], to compare their reactivity and selectivity. Some of them have provided very good levels of asymmetric induction, but their development was often based on empirical approaches, where structural modifications were sequentially tested until the required level of chiral induction was achieved. To carry out a more rational catalyst design, better insight on the mechanisms for this process is needed to understand the effects of structural modifications. However, only a few computational or experimental systematic mechanistic studies are available, and, in most instances, tentative models are elaborated in order to understand the observed enantioselectivity at the molecular level [40,41,42,43]. In the seminal work of Schreiner and co-workers, a detailed evaluation of the possible mechanism of this reaction using density function theory calculations (B3PW91/cc-pVDZ) was reported [40]. These studies were performed using simplified models for both ketimine and catalyst. The calculated transition state for the uncatalyzed reaction involved a four-membered cyclic structure of high energy (ΔH = 40.0 kcal/mol), whereas the enthalpy of activation calculated for the reaction catalyzed by a HSiCl_3_-ligand complex was clearly lower (19.2 kcal/mol). The function of the ligand appeared to be to coordinate with trichlorosilane and serve as a proton donor for the imine. Based on these calculations, the authors proposed a reaction mechanism and explained the experimentally observed stereoselectivity. The catalytic reduction of ketimines with trichlorosilane is described as a formal transfer reaction of H^+^/H^−^ to the C = N double bond. Recently, Jones and co-workers have performed a kinetic study on an imidazole-based catalytic system able to display a dual activation mechanism, leading to high catalytic activity at unprecedented low levels of catalyst loading [41]. This study, however, did not provide any computational studies on these systems to reveal at a molecular level the activation mode proposed. A different mechanistic proposal, involving the participation of two molecules of trichlorosilane, has been recently reported by Dong et al. in the case of axially chiral biscarboline-based alcohols [43].

Natural amino acids are a simple, abundant, and cheap source of chirality for the preparation of organocatalysts [44]. Indeed, most of the catalytic systems described in the literature for the reduction of ketimines with HSiCl_3_ include in their structure an amino-acid-derived fragment as the source of chirality. According to the most general mechanism considered, the Lewis basic sites found in the organocatalyst interact with HSiCl_3_ to form a hexa-coordinated species able to transfer the H^+^/H^−^ couple to the C=N double bond of the ketimine. Taking this into account, any amino-acid-based catalyst for the enantioselective reduction of ketimines with HSiCl_3_ should contain a functional group at the nitrogen providing a second coordination site for the silicon atom of HSiCl_3_. Amine-protecting groups, such as carboxybenzyl (Cbz) or related fragments, contain carbonyl groups that can act as this second coordination site, and a variety of them are commercially available.

**Scheme 1:**
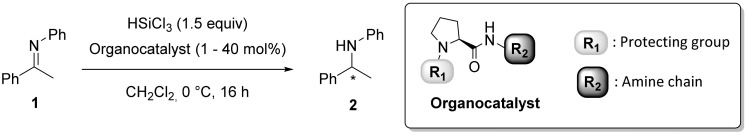
Benchmark asymmetric reduction of ketimine **1** used to develop the organocatalysts in this work.

Here, we report our efforts to design l-proline-based organocatalysts for the reduction of ketimine **1** with HSiCl_3_ as a benchmark reaction (Scheme 1). We have used kinetic and computational studies to shed light on the mechanism of these systems, allowing a rational design of more efficient catalysts in terms of both activity and enantioselectivity.

## 2. Results and Discussion

### 2.1. Catalyst Screening

An initial screening of Cbz-protected amino acids as organocatalysts for the benchmark reduction reaction of ketimine **1** with HSiCl_3_ was performed. All Cbz-protected amino acids (Ala, Phe, His, Trp, Val, and Pro) provided good yields (>85%, entries 2–7, Table S1, Supplementary Materials), but it is important to note that under those standard conditions ([1] = 0.512 M, 1.5 equiv. HSiCl_3_, 40 mol% organocatalyst, CH_2_Cl_2_, 0 °C, 16 h), the uncatalyzed reaction providing the racemic product is important (entry 1, Table S1) [45,46]. Thus, the catalyst role was mainly analyzed through the observed enantioselectivity. The low degree of induction achieved with Cbz-l-Val was surprising, since a variety of catalysts have been reported based on this amino acid [4,5,6,7,47,48,49]. Only appreciable asymmetric induction (62% *ee*) was observed for Cbz-l-Pro, probably due to its cyclic structure that can reduce the conformational freedom at the transition state, favoring an efficient chirality transfer [50]. Therefore, further proline-containing organocatalysts were used in the same reaction. While no asymmetric induction was observed for l-Pro (entry 8, Table S1), the catalytic activity was maintained for functionalization at the l-Pro nitrogen atom using a carbamate group (Boc-l-Pro, entry 9, Table S1) or different amides (*N*-acetyl-l-Pro or Piv-l-Pro, entries 10-11, Table S1), highlighting the key role of the carbonyl group attached to the l-Pro amine group. The enantioselectivity was similar for carbamates Cbz (62% *ee*) and Boc (63% *ee*) and slightly better for amides (*N*-acetyl-l-Pro (68% *ee*) and Piv-l-Pro (77% *ee*). This observation was in line with previous studies suggesting pivaloyl (Piv) as an excellent *N*-substituent in related organocatalysts [42].

**Figure 1:**
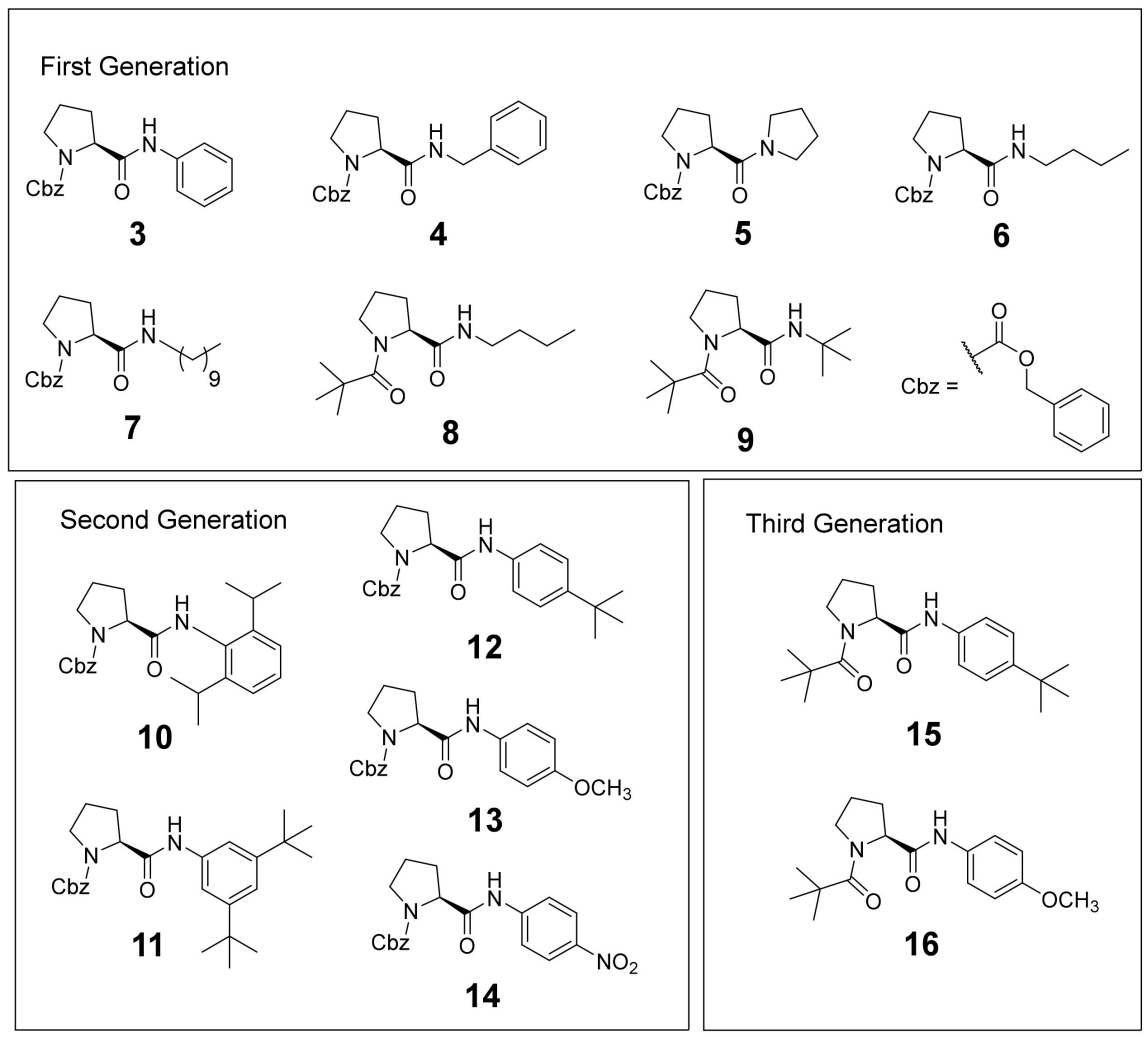
Catalysts used for the reduction of the ketimine **1** in this study.

To further improve the efficiency of these l-Pro systems, a variety of *N*-carbamate-amides and bisamides were designed (Figure 1). The l-Pro amine group was *N*-protected by either Cbz or Piv groups, and the carboxylic group was functionalized using different types of aliphatic and aromatic residues. Synthesis of organocatalysts **3**–**16** was achieved from the corresponding *N*-protected l-proline derivatives [51,52,53,54].

Organocatalysts **3**–**9** (first generation catalysts) were tested for the reduction of the benchmark reduction of ketimine **1** with HSiCl_3_ under the same standard reaction conditions (Table 1). Catalysts **3**, **4**, **6**, **7**, and **8** afforded amine **2** in good yields (>83%). In general, the presence of the amide group in the structure of the catalyst improved the asymmetric induction with regard to Boc-l-Pro (from 62% to 81–85% *ee* for Cbz-l-Pro derivatives). Organocatalysts **5** and **9** show the importance of the proline amide proton in the catalytic cycle, absent in catalyst **5** and strongly sterically hindered in catalyst **9**. For both organocatalysts, racemic product was obtained (entries 3 and 7, Table 1) in good agreement with the mechanistic proposal by Jones [41].

**Figure 2:**
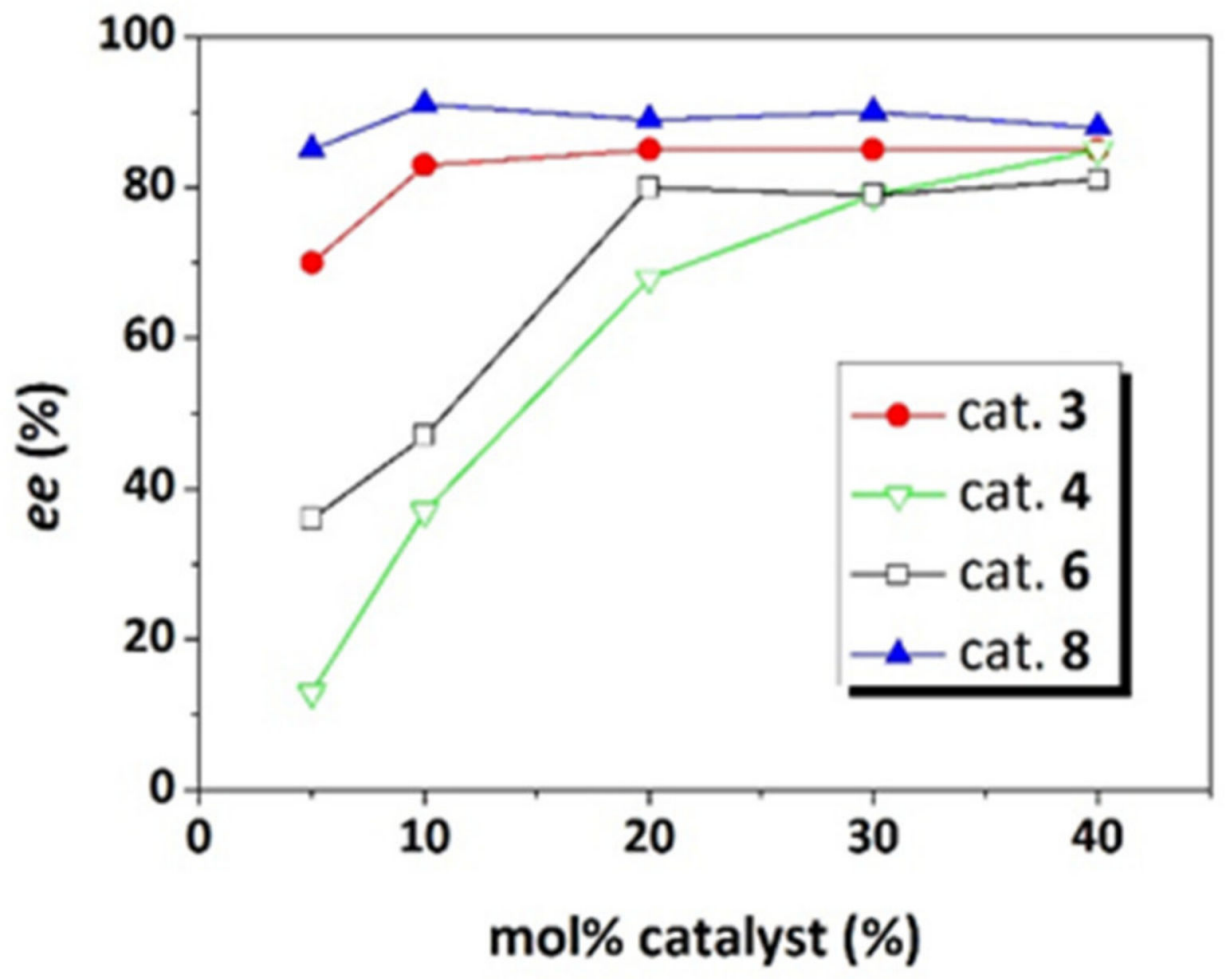
Influence of catalyst loading on enantioselectivity for the reduction of ketimine **1** (0.512 M) with HSiCl_3_ (1.5 equiv.) in CH_2_Cl_2_ at 0 °C for 16 h.

In light of the results in Table 1, the effect of catalyst loading (40 to 5 mol%) was evaluated for catalysts **3**, **4**, **6**, and **8** (Figure 2). While catalysts **4** and **6** showed a significant reduction in enantiocontrol when reducing the amount of catalyst, catalysts **3** and **8** maintained their activity at low catalyst loadings. Catalyst **3** retained the asymmetric induction (83–85% *ee*) until a 10 mol% catalyst loading, with an appreciable decrease (ca. 70% *ee*) for a further decrease in loading to 5 mol%. Catalyst **8** was the most active, displaying only a minor variation of enantioselectivity (85–90% *ee* for 5 to 40 mol% loadings). A good correlation between yield and asymmetric induction was observed, especially for the most active catalysts, having an appreciably quicker rate than the uncatalyzed background reaction conditions (Table S2, Supplementary Materials) [45,46]. Catalyst **8** was also the most active, with only a minor decrease in yield for the lowest catalyst loading (Δyield_5–40%_ ≈ 5% *vs* Δyield_5–40%_ ≈ 36% for compound **4**). Thus, there is a clear relationship between the molecular structure of the catalyst and the chiral induction, which is more relevant for low molar loadings. The comparison of Cbz-l-proline derivatives **3**, **4,** and **6**, just differing on the amide residue, shows that the aniline derivative provided the best results [54]. On the other hand, data for catalysts **6** and **8**, both being butylamine derivatives, suggested that *N*-Piv protection can improve the efficiency significantly. For the most active catalysts, 10 mol% catalyst loading seems to be an excellent option [55].

### 2.2. Kinetic Studies

**Figure 3:**
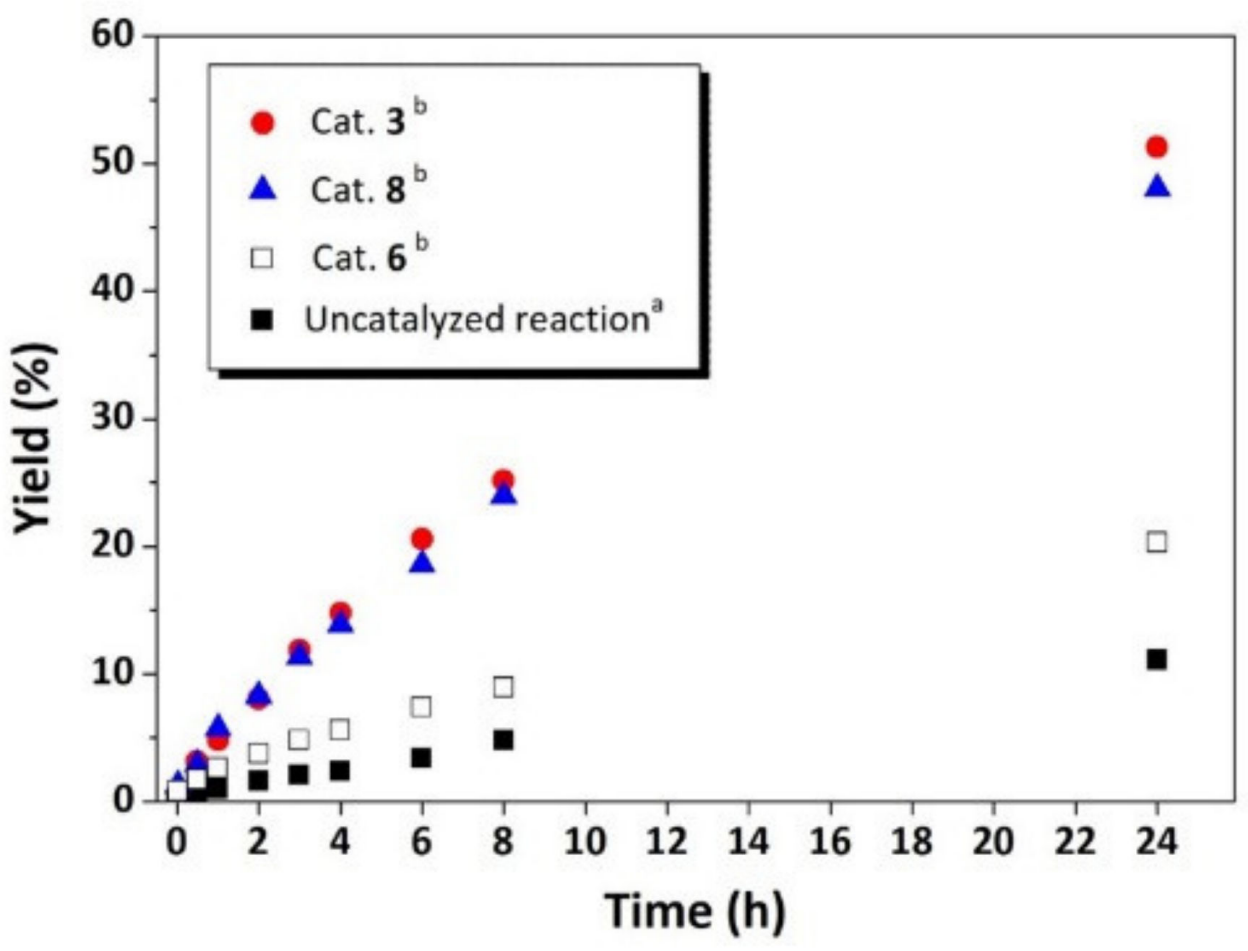
Variation of the yield as a function of time in the asymmetric reduction. See Supplementary Materials (pages S22–S25); *^a^* Concentration **1** = 0.66 M, 1.5 equiv. HSiCl_3_, CDCl_3_, 0 °C; *^b^* Concentration **1** = 0.66 M, [cat.] = 0.066 M, 1.5 equiv. HSiCl_3_, CDCl_3_, 0 °C.

For a better understanding of such differences, a kinetic study was carried out by in situ ^1^H-NMR monitoring of the model reduction using catalysts **3**, **6,** and **8** in CDCl_3_ (0 °C) and a 10 mol% loading. The signals characteristic for the amine formed, and the starting imine and the acetophenone obtained by hydrolysis of the imine were monitored (Figure S1, Supplementary Materials). The resulting kinetic profiles are presented in Figure 3. Changing the solvent from CH_2_Cl_2_ to CHCl_3_ in this kind of reaction often provides lower *ee* values and slower reaction rates [54,56,57,58], but slower kinetics can enable more accurate NMR kinetic analyses and reduce the contribution of the uncatalyzed reaction.

**Scheme 2:**
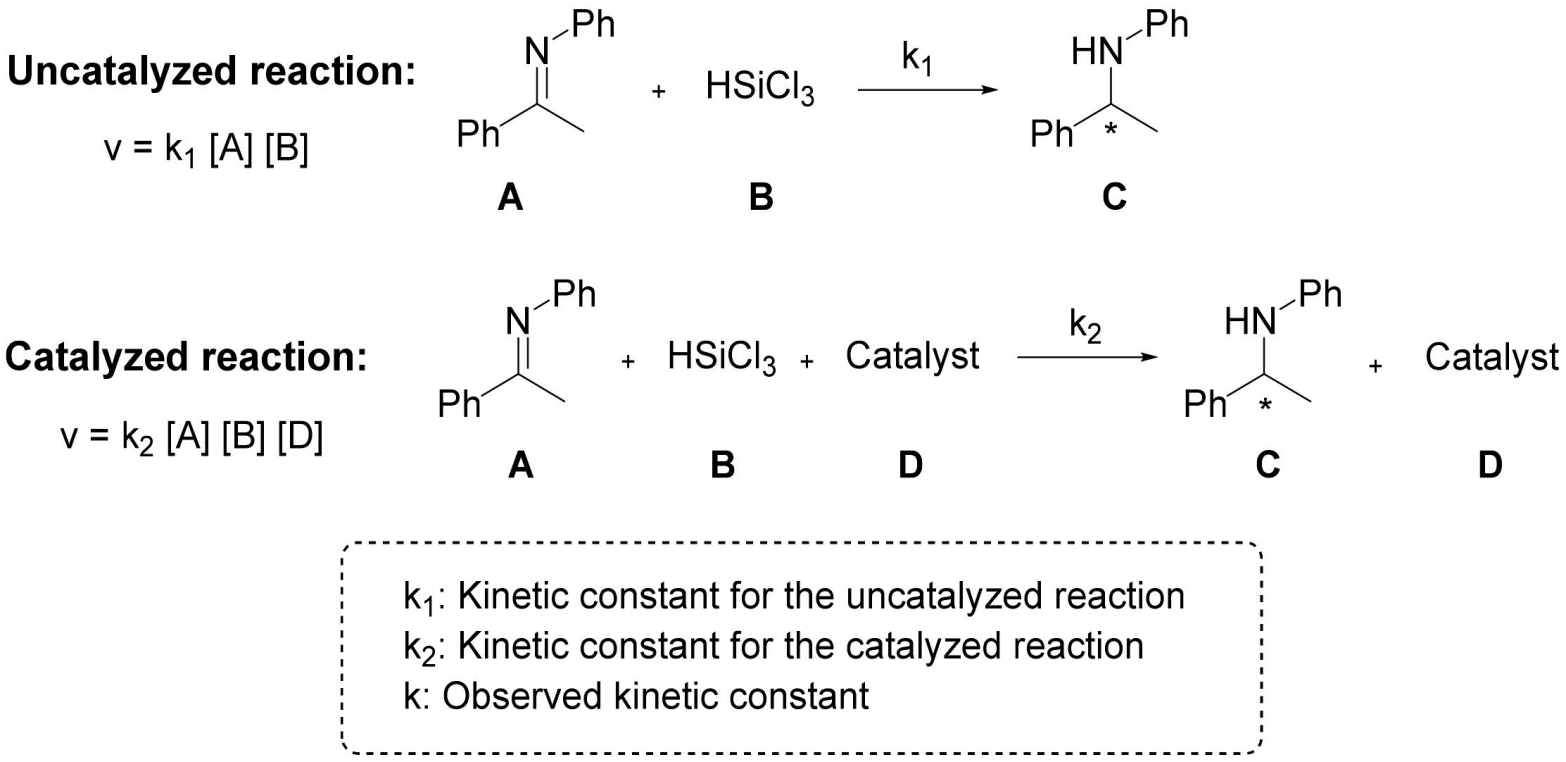
Kinetic model for uncatalyzed and catalyzed reactions.

The rate constants were calculated considering a simultaneous contribution of the uncatalyzed and catalyzed reactions with first-order rate equations in both substrate and reagent, in agreement with most mechanistic studies (Scheme 2 and Equation (1)) [24,25,26,27,28,29,30,31,32,33,34,35,36,37,38,39,40,41,42]. Although for axially chiral biscarboline-based alcohols, the participation of two molecules of HSiCl_3_ has been included in the reaction mechanism, to consider the quantitative reaction of this reagent with alcohols to produce hydrogen, this could be discarded in our case, as this process was not observed [43]. Accordingly, the uncatalyzed reaction has a rate v = k_1_[A][B] and can be obtained for the reaction in the absence of any catalyst. The catalyzed reaction should be defined as v = k_cat_[A][B] but taking into account the analysis of the effects of catalyst concentration, k_cat_ can be defined as k_2_[D], while k represents the global experimental constant of the process (Equation (1)). The integration of Equation (1) provides the kinetic model (Equation (2)). Although in one instance, nonlinear effects (NLE) have indicated the participation of two catalyst molecules in the mechanism [59], NLE data have confirmed in most instances the involvement of one single catalyst molecule in the mechanism, in particular for catalytic systems related to the ones in this study and involving a dual activation mechanism [24,25,26,27,28,29,30,31,32,33,34,35,36,37,38,39,40,41,42,43].

(1) $$v_{c}=\frac{d\left[C\right]}{\mathrm{dt}}{=k}_{1}\left[A\right]\left[B\right]{+k}_{2}\left[A\right]\left[B\right]\left[D\right]\;=\;\left(k_{1}{+k}_{2}\left[D\right]\right)\left[A\right][B]=k\left[A\right]\left[B\right]$$

(2) $${\left[C\right]}_{\mathrm{theoretical}}=\frac{{\left[A\right]}_{0}{\left[B\right]}_{0}{(1\;-\;e}^{\left({\left[B\right]}_{0}\;-\;{\left[A\right]}_{0}\right)\mathrm{kt}})}{{\left[A\right]}_{0}-\;{\left[B\right]}_{0}{\;\times \;e}^{\left({\left[B\right]}_{0}\;-\;{\left[A\right]}_{0}\right)\mathrm{kt}}}$$

Fitting the experimental data in the presence of catalyst to Equation (2) allowed us to obtain the experimental kinetic constant (k). From this kinetic constant, it was possible to obtain the value of k_2_ from the concentration of catalyst and the uncatalyzed kinetic constant (k_1_). Table 2 summarizes the values of the rate constants obtained in this way. The fittings results confirmed the validity of the proposed kinetic model (see, for instance, Figure S2, Supplementary Materials).

The k_2_/k_1_ coefficient facilitates an easy comparison of the different catalysts. Thus, the most active one was catalyst **3**, displaying a kinetic constant for the catalyzed reaction almost 100 times higher than the one for the uncatalyzed process (entries 1 and 2, Table 2). Data also show that the substitution of the Cbz group for the Piv group increases the reaction rate of the catalyzed reduction (entries 3 and 4, Table 2).

It must be borne in mind that a higher activity of the catalyst can significantly enhance the asymmetric induction, as the uncatalyzed reaction, affording a racemic mixture, will reduce its contribution. Thus, Equation (1) shows that it is required that k_2_[D] >> k_1_ for fast and highly enantioselective processes, and the concentration of the catalyst ([D]) can play a key role. Accordingly, two additional sets of experiments were performed. Initially, different concentrations of catalyst **6** (from 0.50 to 0.01 M) were analyzed, but keeping constant the substrate–catalyst ratio (40 mol%) by simultaneously reducing the concentration of imine **1** (Table S3, Supplementary Materials). For catalyst concentrations above 0.2 M, yield and enantioselectivity were high and almost identical (yield > 90%, 81% *ee*, entries 1–2, Table S3). A decrease of the concentration to 0.1 M led to some activity reduction (65% yield, entry 3, Table S3), although enantioselectivity was maintained (84% *ee*). However, a further tenfold reduction of catalyst concentration produced a drastic reduction of both activity and asymmetric induction (9% yield, 40% *ee*, entry 4, Table S3). In an additional study, the concentration of catalyst **6** was kept constant at 0.205 M, but its molar percentage was varied from 5 to 40 mol% (Table S4, Supplementary Materials). The reduction in catalyst loading was accompanied by a decrease from 94 to 70% in yield and from 81 to 65% in enantiomeric excess. Therefore, the activity and enantioselectivity of catalyst **6** is influenced by both its concentration and the catalyst–substrate ratio (Figure S3, Supplementary Materials). Interestingly, when the catalyst molar concentration was kept high enough, an appreciable efficiency was observed even for low molar loadings. As suggested by data in Figure 2, this dependence is very sensitive to catalyst structure. Thus, catalyst **8** maintained the enantioselectivity for the reduction of **1** even at low concentrations and for small catalyst loadings (5 mol%) (Cat **8** = 0.026 M, 85% *ee*; Cat **8** = 0.205 M, 82% *ee*, Table S5, Supplementary Materials). These differences can be explained by taking into account Equation (1). For catalyst **6**, k_2_ is relatively close to k_1_, and when its concentration decreases, the product k_2_[A][B][D] becomes rather small, with the uncatalyzed reaction increasing its contribution and leading to an important reduction in enantioselectivity. In contrast, for catalyst **8,** k_2_ is almost 90 times larger than k_1_. Thus, a much larger reduction in the concentration of **8** is needed to observe a significant increase in the relative importance of the uncatalyzed reaction. Formation of intermolecular aggregates with the increase of the concentration of catalyst could also contribute to this phenomenon, but ^1^H-NMR experiments with catalysts **6** and **8** at different concentrations and temperatures discarded a significant involvement of them (Figure S4, Supplementary Materials).

### 2.3. Structural Catalyst Optimization

**Figure 4:**
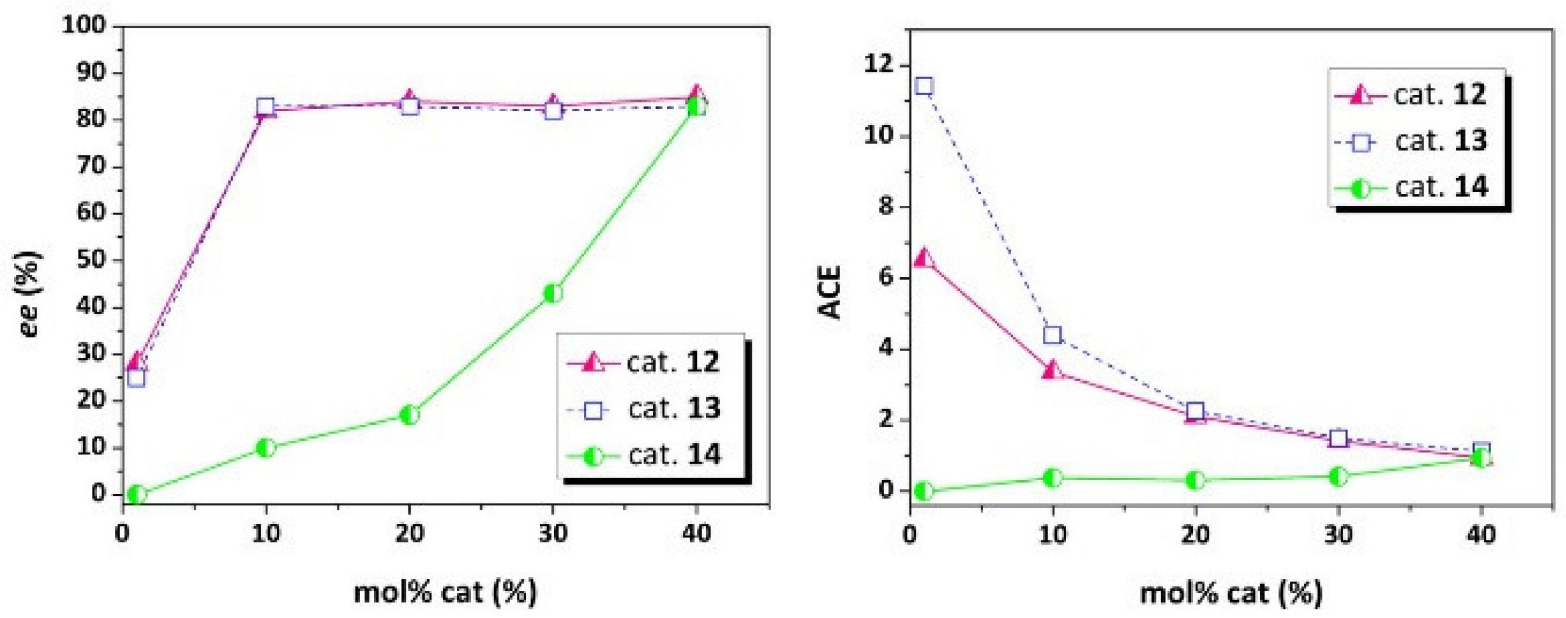
Variation of enantioselectivity and ACE as a function of the molar percentage of catalysts **12**, **13,** and **14**. Reaction conditions: Concentration **1** = 0.512 M, 1.5 equiv. HSiCl_3_, CH_2_Cl_2_, 0 °C, 16 h.

The former catalyst screening and computational calculations run in parallel (see below), provided two key elements for catalyst optimization: the use of *N*-protected prolinamides derived from aromatic amines and the use of the pivaloyl protecting group instead of Cbz. Besides, computational studies suggested that aromatic substitution could have important effect. Thus, Cbz-protected compounds **10**–**14** (second-generation catalysts) were synthetized, and their behavior against the molar percentage, at a constant imine concentration (0.512 M in CH_2_Cl_2_), was also evaluated (Table S6, Supplementary Materials). Catalyst **10** showed a good activity but no enantioselectivity at 40 mol% (91% yield, 4% *ee*, entry 1, Table S6, Supplementary Materials), while catalyst **11** presented moderate-low activity and selectivity under the same reaction conditions (78% yield, 50% *ee*, entry 2, Table S6), highlighting again the importance of the amide NH fragment and its environment, with groups providing significant steric hindrance in its surroundings, contributing to a decrease in the level of enantioselectivity afforded. Interestingly, catalysts **12** and **13** maintained their efficiency up to 10 mol% loading (83% *ee* in both cases, Figure 4 and Table S6), but with an ACE (asymmetric catalyst efficiency [60]: a formula that takes into account the amount of catalyst employed and the relative size of the catalyst to the product, as well as the *ee* and yield of the product, in order to measure the catalyst efficiency) slightly higher at this loading for **13** (4.39 vs. 3.35). These results were comparable with those for catalyst **3** (83% *ee* for 10% catalyst loading, ACE = 4.44, entry 2, Table S2). Finally, catalyst **14** only afforded an enantiomeric excess above 80% when a 40 mol% loading was used.

Kinetic constants (in CDCl_3_) for catalysts **12–14** are presented in Table 3 (see also Figure S5, Supplementary Materials). For catalysts displaying electron donor groups on the aromatic ring (**12**, R = *tert*-butyl; **13**, R = -OCH_3_), k_2_/k_1_ values were approximately 58 and 106. However, for the catalyst with an electron acceptor group on the aromatic ring (**14**, R = -NO_2_), both constants were relatively similar, and therefore the enantioselectivity was low (10% *ee*). For this last catalyst (**14**), it must be noted, however, that a complex reaction crude was observed, which can be associated with the potential reduction of the NO_2_ group, not only consuming part of the HSiCl_3_ but also potentially producing a different catalytic system [15,16].

**Figure 5:**
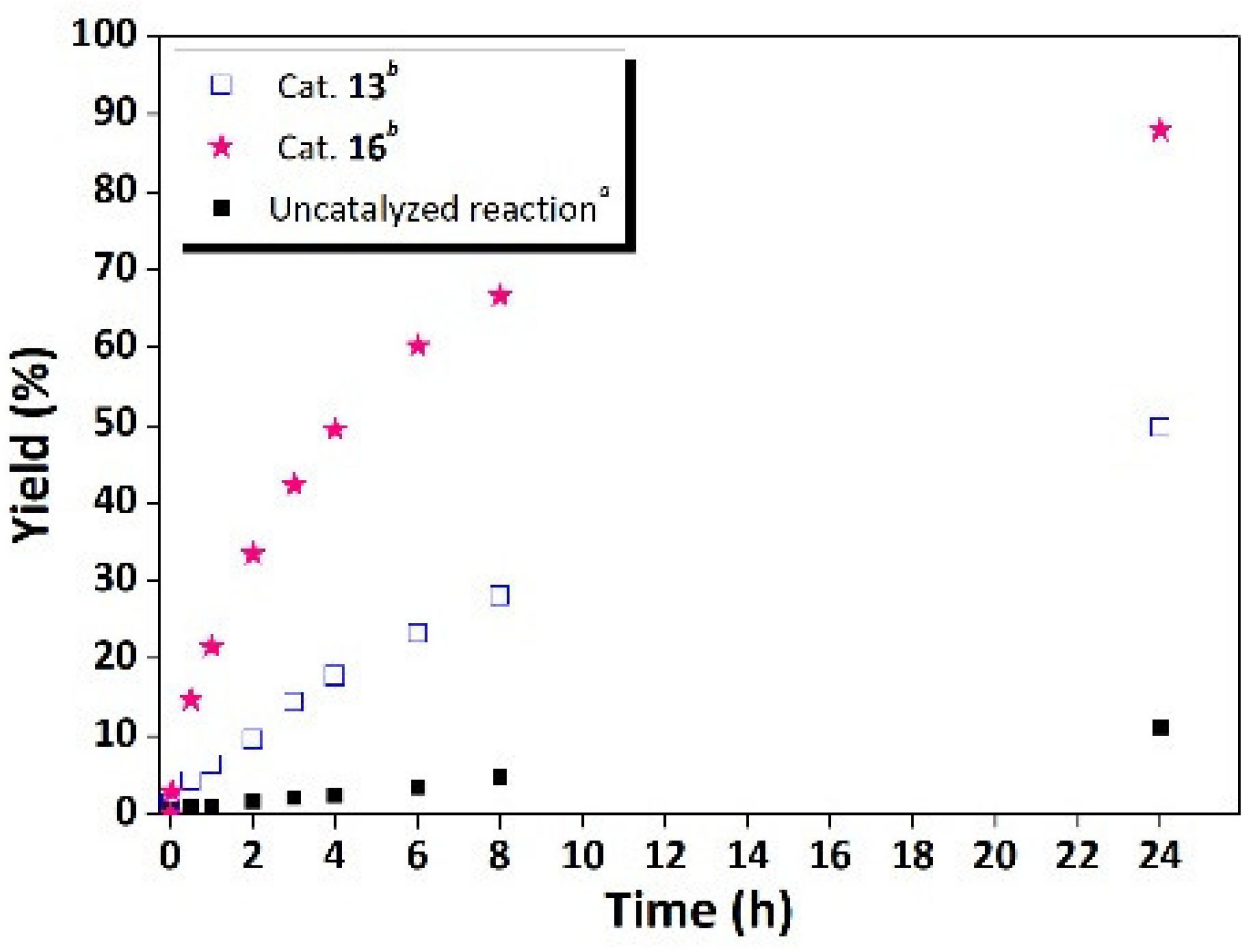
Comparison of kinetic curves for catalysts **13** and **16** with different *N*-protecting groups. See Supplementary Materials (pages S27 and S29). *^a^* Concentration **1** = 0.66 M, 1.5 equiv. HSiCl_3_, 0 °C; *^b^* Concentration **1** = 0.66 M, (catalyst) = 0.066 M, 1.5 equiv. HSiCl_3_, 0 °C.

A second catalyst optimization step was performed by changing the *N*-protecting group from Cbz to Piv (third-generation catalysts). Thus, catalysts **15** and **16** were evaluated, and the results (Table 4) were compared with those for related **12** and **13**. Both catalysts allowed the obtainment of the chiral amine with good enantioselectivity using only 1 mol% of catalyst (entries 1 and 4, Table 4), increasing the ACE values to 47.87 for **15** and to 49.05 for **16**. The effect of the substitution of Cbz by Piv was also analyzed by monitoring the kinetic profile by ^1^H-NMR (in CDCl_3_) for the most efficient catalyst **16** (Figure 5 and Supplementary Materials, pages S27 and S29), revealing an increase in the observed kinetic constant (k) by almost one order of magnitude when compared with the related catalyst **13** (0.21395 and 0.04326 for **16** and **13**, respectively, k_2_ = 3.1594 for **16**). In fact, the k_2_/k_1_ ratio for **16** was 582, confirming this compound as the best catalyst in this study, leading to the highest enantioselectivity (86% *ee*) with the lowest amount of catalyst (1 mol%, ACE = 49.05).

### 2.4. Continuous Flow Reduction

Thus, mechanistic studies and experimental screening allowed the selection of prolinamide **16** as a simple and cheap catalyst with enhanced activity and enantioselectivity for the process considered. In batch, with just a 1 mol% catalyst loading of **16** and three hours of reaction, the chiral amine **2** was obtained in 98% yield and 82% *ee*. This facilitated the performance of the imine enantioselective reduction under continuous flow conditions. For this purpose, two flows, one containing HSiCl_3_ and the second the catalyst **16** and imine **1**, were combined in a mixing valve and were pumped through a tubular reactor. Both flows were adjusted to reach a 10 mol% catalyst loading at the exit of the mixing valve, and samples were taken at different times after the reactor. Once the steady state was reached, a constant yield of 90% with an enantiomeric excess of 87% was obtained for more than nine hours (Figure S6, Supplementary Materials).

### 2.5. Computational Studies with Simplified Model Compounds

**Figure 6:**
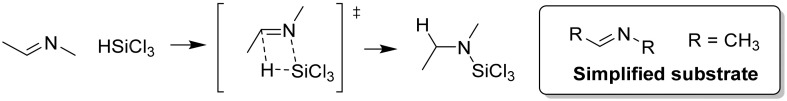
Uncatalyzed reduction of an imine.

In order to better understand the mechanism for this process and rationalize catalyst optimization results, computational studies were carried out in parallel, following the sequential approach described before for experimental studies. The results will be discussed here in an integrated way for simplicity. Besides some tentative mechanistic models, there are only two reports providing detailed theoretical calculations based on DFT studies, although the mechanism considered in the more recent one cannot be applied in our case [40,43]. The original DFT-based mechanism, using simplified models of the imine and catalyst proposed, involved the formation of a catalyst–HSiCl_3_ complex capable of acting in a formal H^+^/H^−^ transfer to the C=N bond [40]. Based on this antecedent, and trying to identify the role of the different structural factors, the faster PM6 semiempirical method was chosen, in order to be able to work with structures closer to the real ones, and calculations were performed with the Gaussian 09 program [61]. To validate this method, the uncatalyzed reaction pathway was initially studied with the simplified imine model used by Schreiner. In this process (Figure 6), the reactants interaction complex (RIC) formed by the imine and HSiCl_3_ evolves to a transition state (TS) of four centers, transferring the hydrogen of trichlorosilane to the imine carbon atom and giving rise to the products interaction complex (PIC). The calculated energy barrier was 40 kcal/mol, as in the DFT calculations [40], and, therefore, this method of calculation, facilitating the analysis of the effects of structural changes in the catalyst, was considered valid.

**Figure 7:**
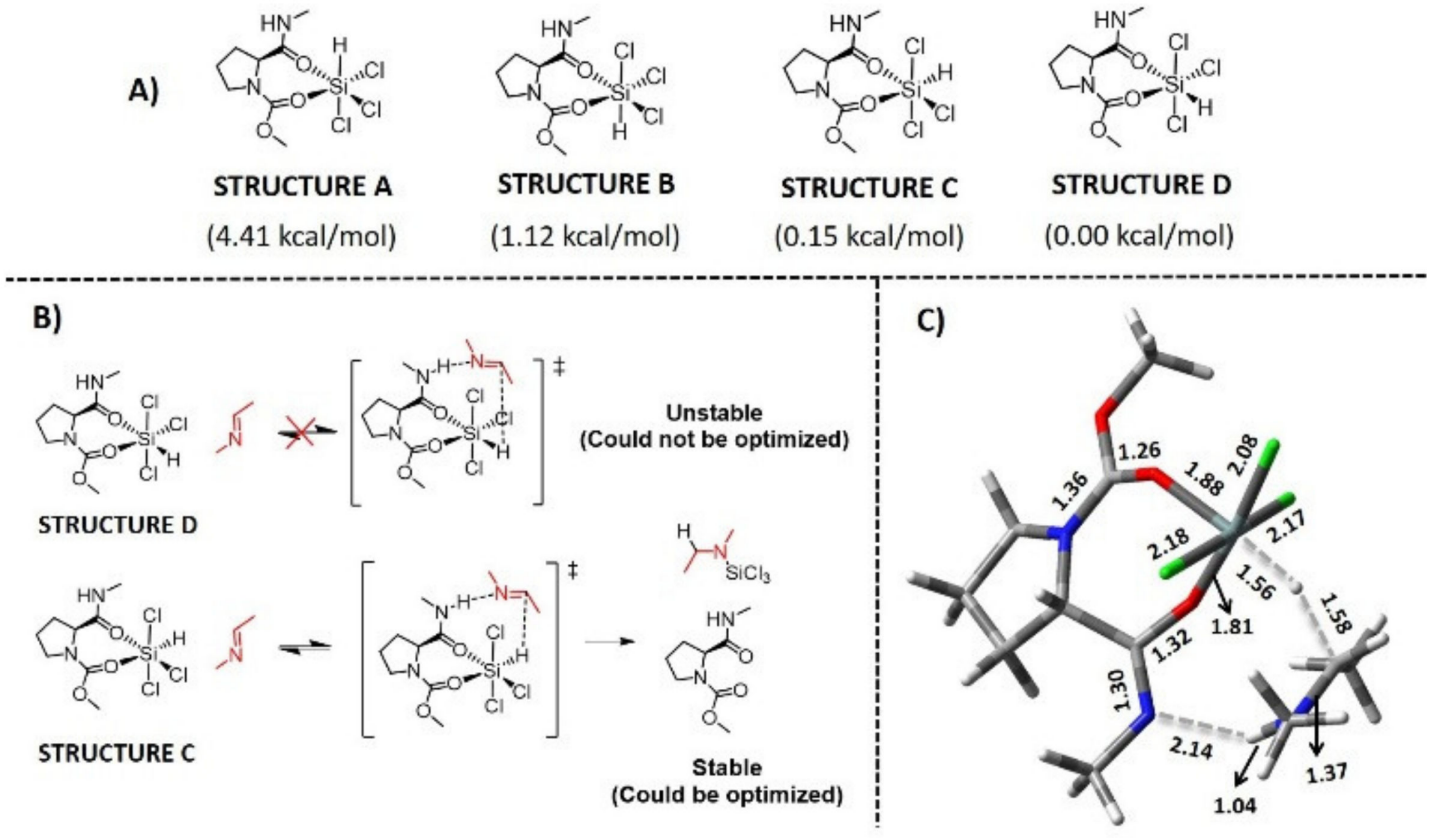
(**A**) Calculated structures and energies for the catalyst –HSiCl_3_ complex. (**B**) Reaction scheme from structures C and D. (**C**) Optimized geometry for the TS calculated from structure C.

The conformational space of a model for catalyst **6**, where butyl and benzyl groups were replaced with methyl groups, was then studied using the same imine model presented in Figure 6. In agreement with its ^1^H-NMR spectrum displaying two amide NH signals of similar intensity (Figure S4A, Supplementary Materials), the two possible intramolecular amide NH hydrogen bonds in Cbz-protected compounds were considered (Figures S7 and S8A, Supplementary Materials). Both of them seem capable of interacting with HSiCl_3_ to form an hexacoordinate complex (Figure S8B, Supplementary Materials) in which the silicon atom is bound to the two carbonyl oxygens of the catalyst, requiring the breaking of the previous intramolecular hydrogen bonds. This complex can present different structures depending on the distribution around the silicon atom (Figure 7A). Since the structures with the hydrogen in equatorial position showed lower energy than the apical position ones, the transition state was evaluated exclusively with structures C and D. Only with structure C (Figure 7B) was it possible to obtain an optimized geometry of the TS (Figure 7C).

**Figure 8:**
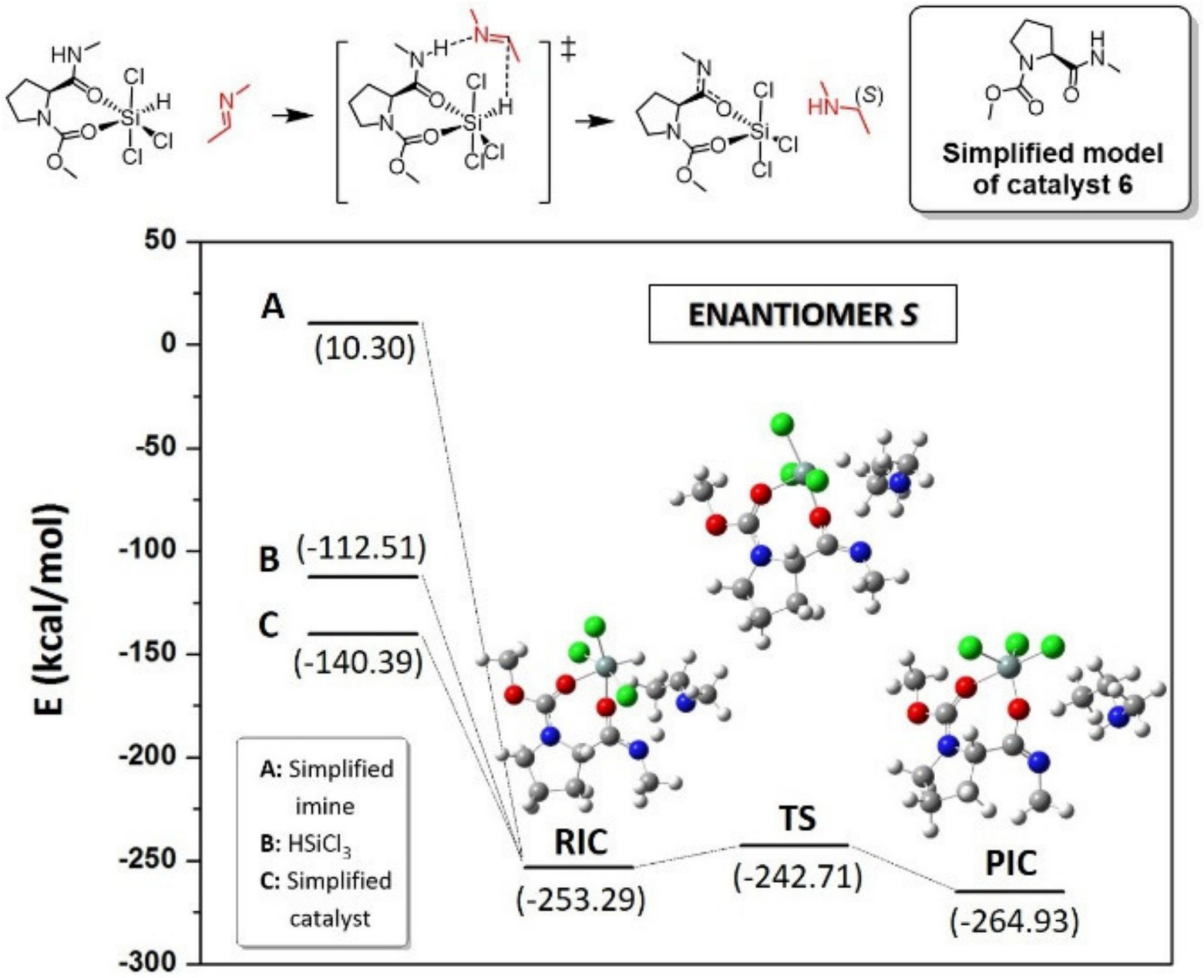
Calculated energy profile for the simplified model reaction.

Figure 8 shows the energy profile for this simplified model of catalyst **6** and the calculated energies for the optimized geometries of reagents and the corresponding RIC, TS, and PIC. The energy barrier from RIC to TS decreases for the catalyzed process from 40 to 10.59 kcal/mol. This barrier is lower than the one calculated by Schreiner (19.1 kcal/mol) [40], highlighting the relevance of the substitution pattern at the two carbonyls coordinated to silicon.

**Figure 9:**
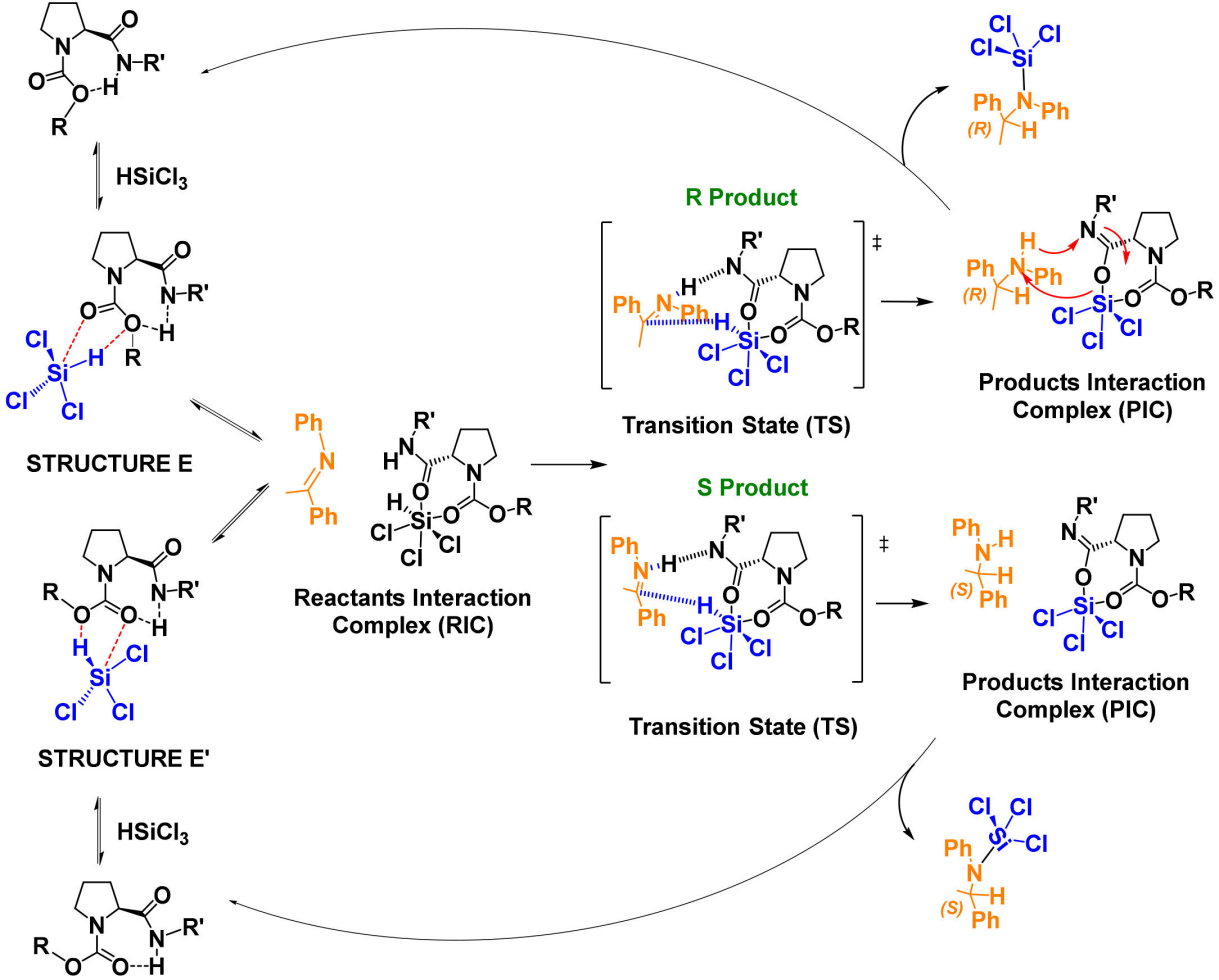
Reaction mechanism considered for the hydrogenation of ketimine **1** with the corresponding *N*-carbamate-amide in the presence of HSiCl_3_.

Thus, the overall mechanism to be considered for *N*-carbamate-proline catalysts (Figure 9) involves the initial interaction of HSiCl_3_ with the two possible conformations of the *N*-carbamate-amide to form weak complexes that evolve towards a common RIC in the presence of imine. The resulting TS presents a N_amide_···H···N_imine_ interaction that results in the new amine N-H bond, while, simultaneously, the Si···H···C_imine_ interaction leads to the formation of the C-H bond at the new stereogenic carbon atom of the amine. Additional supramolecular interactions, which have been suggested to be relevant for this and related processes, in particular those involving the aromatic rings, can also contribute to stabilizing the TS and decreasing the energy barrier [24,25,26,42,62,63,64]. The subsequent PIC affords the final product (chiral amine–SiCl_3_) by a formal proton transfer of the amine to the catalyst–SiCl_3_ complex, which is recovered for a new catalytic cycle. This mechanism is in line with the dual activation model described for imidazole-derived compounds and other organocatalysts [41,65].

### 2.6. Mechanistic Studies with Real Compounds

In a second step, the energy profiles of a series of *N*-carbamate-amides differing in the amide substituents (compounds **3**, **5**, **6**, **12**, **13**, and **14**) and for the Piv derivative **16** were calculated (Table S7, Supplementary Materials). The real structures of the catalysts and imine **1** were used to calculate the energy of the interaction complexes (for both enantiomers) and the energy barrier between RIC and TS. Calculations revealed that in the optimized structure of the catalyst–HSiCl_3_ chelate complex, the seven-member ring can acquire two boat conformations, depending on the position of the amide NH-R group (Figure S9, Supplementary Materials, *up*- and *down*-conformation), and both conformations were considered for the calculations [66,67]. The reaction profile obtained for the pyrrolidine-derived catalyst **5** confirmed the crucial role of the N_amide_···H···N_imine_ interaction. Lacking the amide hydrogen, the value of ΔE_RIC-TS_ was very high (48.87 kcal/mol, entry 3, Table S7, Supplementary Materials) for the *R* product, and it was not possible to obtain the energies of the interaction complexes for the *S* product. For the other catalysts, the energy barrier ΔE_RIC-TS_ was always lower than for the uncatalyzed reaction. However, based on the different ΔE_RIC-TS_ obtained, calculations predicted that the reaction rates for those compounds should follow the trend v**_14_** > v**_3_** > v**_12_** > v**_13_** > v_16_ > v**_6_** > v**_uncat_**, while the observed experimental order was v**_16_** > v**_13_** > v**_3_** > v**_12_** > v**_6_** > v**_14_** > v**_uncat_**. Although the order for catalyst **14** can be rationalized in terms of the side reactions observed, it seems clear that important discrepancies are present in both trends. According to Equation (3), a linear relationship between ln k and the energy barrier should be expected, but when the ΔE_RIC-TS_ energy barrier was compared with the experimental rate constant obtained for the catalyzed process (k_2_), since computational studies only considered the catalyzed reaction, this relationship was not observed (Figure S10, Supplementary Materials).

(3) $$k\;=\;\frac{k_{B}\cdot T}{h}\times e^{-\frac{\Delta G‡}{\mathrm{RT}}}$$

(4) $$\frac{P_{S}}{P_{R}}=\frac{k_{S}}{k_{R}}=e^{-\delta \Delta E_{\mathrm{RIC}-\mathrm{TS}}/\mathrm{RT}}\;\delta \Delta E_{\mathrm{RIC}-\mathrm{TS}}=\Delta E_{\mathrm{RIC}-\mathrm{TS}}\left(S\right)-\Delta E_{\mathrm{RIC}-\mathrm{TS}}\left(R\right)\;ee\;\left(\%\right)=\frac{e^{\left(\Delta E_{\mathrm{RIC}-\mathrm{TS}}\left(S\right)-\Delta E_{\mathrm{RIC}-\mathrm{TS}}\left(R\right)\right)/\mathrm{RT}}-1}{e^{\left(\Delta E_{\mathrm{RIC}-\mathrm{TS}}\left(S\right)-\Delta E_{\mathrm{RIC}-\mathrm{TS}}\left(R\right)\right)/\mathrm{RT}}+1}\times 100$$

The barrier for the formation of the *S* enantiomer was always lower than for the *R* (Figure S11, Supplementary Materials), in excellent agreement with the preferential formation of the *S*-amine in the corresponding experiments. In principle, the predicted enantiomeric excesses could be estimated by comparing the energy barriers differences for the formation of both enantiomers (Equation (4)) [43]. Thus, *ee*_calcd._ values were obtained using ΔE_RIC-TS_ for each enantiomer, as the average of the values for the *up* and *down* conformations (*ee*_calcd._) of the catalyst–HSiCl_3_ complex. However, some significant discrepancies were observed between *ee*_calcd._ and *ee*_exp._ values (Table S8, Supplementary Materials). Thus, the predicted enantiomeric excess was lower than the experimental one for **3** when considering a 10 mol% catalyst loading (32% *ee*_calcd._ < 76% *ee*_exp._, entry 1, Table S8), while it was more comparable for **6** (70% *ee*_calcd._ ≈ 63% *ee*_exp._, entry 2, Table S8).

All the discrepancies observed in the former calculations could be explained because these calculations were performed considering only the energy barrier between RIC and TS (ΔE_RIC-TS_), and RIC formation was not taken into account. According to the calculations, the observed rate should be dependent on the value of ΔE_RIC-TS_ and on (RIC). For a situation in which the RIC is quantitatively formed from the substrates and the catalyst, (RIC) would directly reflect their concentrations, and a direct comparison with the experimental rates is possible. However, if the RIC involves the formation of a weak complex, then its concentration, for a given concentration of substrates and catalyst, can significantly differ according to the structure of the catalyst. Overall, this suggests, along with the observed structure-sensitive dependence of the enantioselectivity from catalyst loading and concentration, that the formation of the catalyst–HSiCl_3_ complex (i.e., structures E and E’ in Figure 9), requiring the breaking of intramolecular hydrogen bonds and modifying of the relative disposition of the carbonyl groups to adopt a *cis* conformation, can be an important step to define the relative contribution of the catalyzed/uncatalyzed reactions and accordingly the observed enantioselectivity. When a solution of catalyst **6** and trichlorosilane in CDCl_3_, at the same concentrations used for imine reduction, was studied by ^1^H- NMR, the resulting spectra showed one single signal for H-Si (the same for ^29^Si-NMR) and just some changes in the chemical shifts of the signals for the two intramolecularly hydrogen-bonded conformers present in **6** (Figure S12, Supplementary Materials). This ruled out the quantitative formation of the **6**–HSiCl_3_ complex contributing to the RIC and indicated the presence of weak **6**–HSiCl_3_ complexes in fast equilibria (i.e., structures E and E’ in Figure 9) as the major component. Spectra taken just after addition of imine **1** again showed a similar behavior, displaying some additional shifts for the signals. The substitution of the Z group by pivaloyl (catalyst **8**) afforded similar observations, although in this case, one single intramolecularly hydrogen-bonded structure is present (Figure S13, Supplementary Materials).

**Figure 10:**
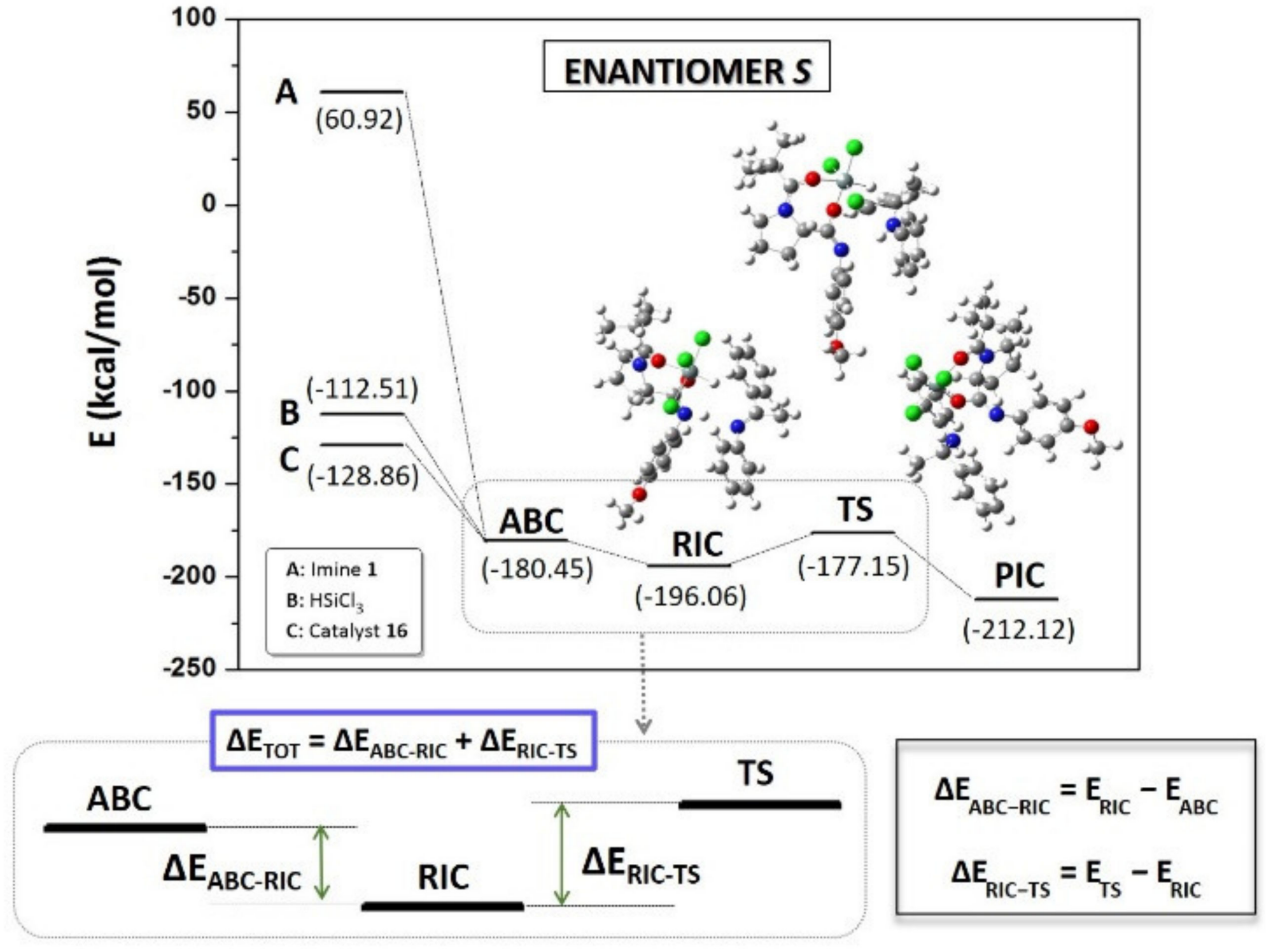
Energy profile calculated for the enantioselective reduction of imine **1** using catalyst **16**, obtaining the *S*-amine **2** as the product.

To take this issue into account, the energy of the initial conformations for the different catalysts was also calculated (Table S9, Supplementary Materials) and used to obtain an initial coordinate (ABC) that would be defined by the sum of reagents energies (E_ABC_) to be included in the corresponding energy profile (Figure 10) [43]. Thus, the contribution for the formation of the RIC (requiring hydrogen bond breaking and conformational rearrangements) can be associated to ΔE_ABC_-_RIC_ = E_RIC_ − E_ABC_, and a parameter (ΔE_TOT_) can be defined as ΔE_TOT_ = ΔE_ABC_-_RIC_ + ΔE_RIC_-_TS_. In this approach, theoretical *ee* was determined using this parameter (ΔE_TOT_, including both the energy barrier to the TS and a factor to consider the (RIC)) and Equation (5).

(5) $$\begin{array}{c} \frac{P_{S}}{P_{R}}=\frac{k_{S}}{k_{R}}=e^{-\delta \Delta E_{\mathrm{TOT}}/\mathrm{RT}}\\ \mathrm{where}\;\delta \Delta E_{\mathrm{TOT}}=\Delta E_{\mathrm{TOT}}\left(S\right)-\Delta E_{\mathrm{TOT}}\left(R\right)\\ e\;(\%)=\frac{e^{-{\delta \Delta E}_{\mathrm{TOT}}/\mathrm{RT}}-1}{e^{-{\delta \Delta E}_{\mathrm{TOT}}/\mathrm{RT}}+1}\;\times \;100 \end{array}$$

**Figure 11:**
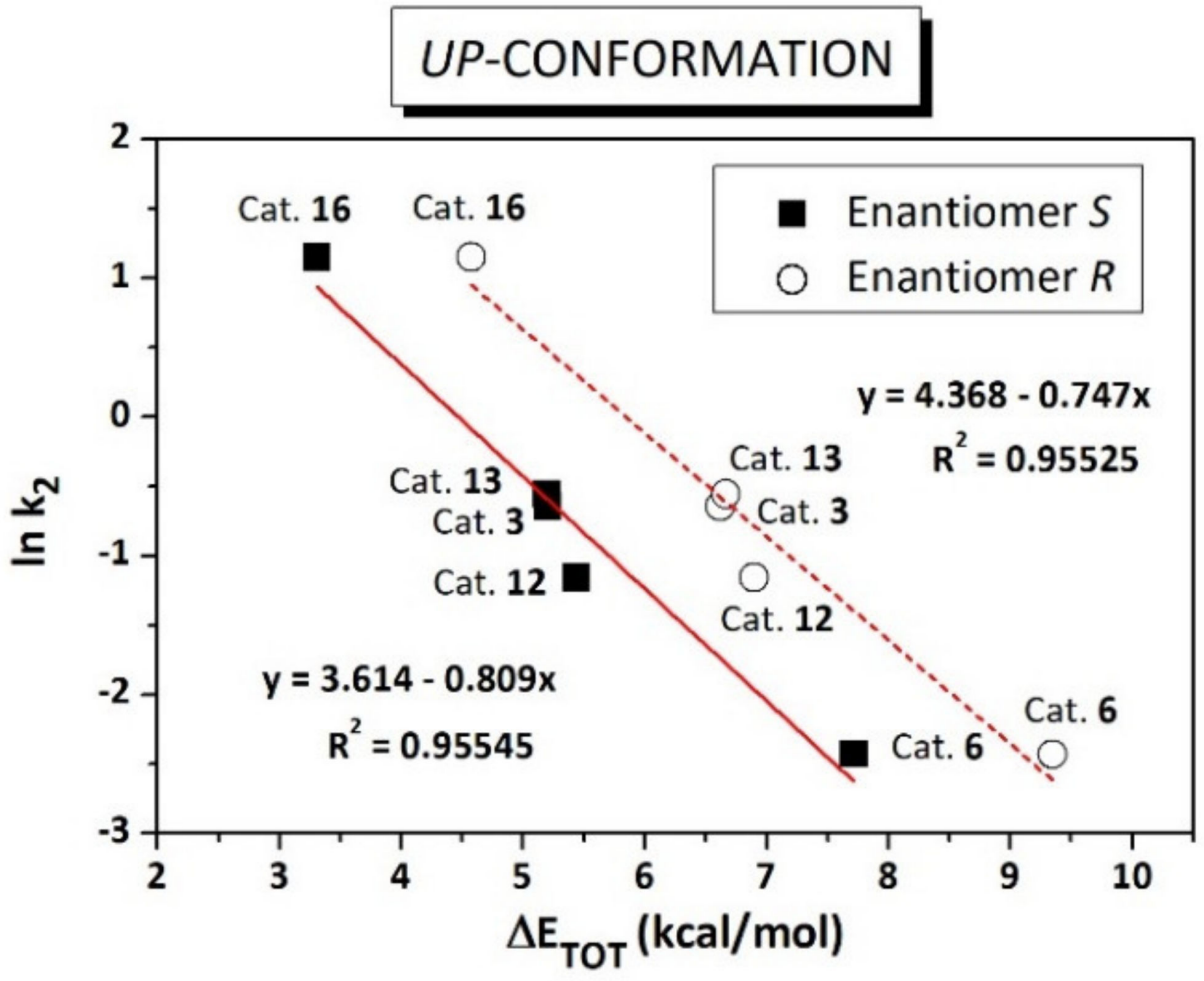
Representation of ln k_2_ (experimental) versus ΔE_TOT_ (theoretical), and linear fitting (not including catalyst **14**) for *R* and *S* enantiomers in the *up* conformation.

The values of ΔE_TOT_ obtained using this approximation, as well as the energy difference for the *R* or *S* enantiomer (δΔE_TOT_), allowed us to calculate a new set of expected *ee* values for each conformer (*up* and *down*) and the corresponding average value (Table S10, Supplementary Materials). The deviation between calculated and experimental values of *ee* was less than 6% for most of the catalysts. Only **6** displayed a deviation of ca. 17%. Therefore, a better fit was obtained in this case between theoretical and experimental enantioselectivity after discarding catalyst **14** according to the former discussion (Figure S14, Supplementary Materials). Figure 11 shows the linear relationship between the experimental rate constants and ΔE_TOT_ for the *up* conformation (see also *down* conformation in Figure S15, Supplementary Materials). Thus, the theoretical model developed in this work is able to reproduce in a reasonable way the results observed experimentally.

## 3. Materials and Methods

### 3.1. Experimental Section

All reagents and solvents were obtained from commercial sources (Aldrich, Fluka, Scharlab or Iris-Biotech) and used without further purification unless otherwise noted. Air and/or moisture-sensitive reactions were carried out under an inert atmosphere of nitrogen, using glass material previously dried in the oven and dry solvents supplied by a Pure Solv model-solvent-dispensing system from Innovative Technology. Moisture-sensitive reagents were handled using syringes under inert atmosphere. Reactions whose procedure required low temperature for extended time were carried out with the aid of a Neslab model CC-100 II Cryocool. Purification of synthesized products was generally performed by crystallization. In cases where it was not possible, column chromatography was performed using a silica gel 60 stationary phase with a particle size of 0.06–0.2 mm. For each occasion, the eluent used has been indicated, as well as the proportions of the solvents in volume–volume. After obtaining synthesized products, they were dried in a Binder vacuum oven at a temperature of 45 °C and then stored in a desiccator or refrigerator according to the needs of the product. Enantiomeric excess of the imine reduction was determined by high-performance liquid chromatography with a Merck HITACHI LaChrom D-7000 chromatograph and a Chiralcel OD-H chiral filler column (4.6 mm φ × 250 mm L). HPLC conditions: *n*-hexane/MTBE (98/2), 1 mL/min, 30 °C, 254 nm (UV/Vis detection), t_R_ (*S*-amine): 26 min, t_R_ (*R*-amine): 27.5 min. ^1^H-NMR and ^13^C-NMR spectra were recorded on a Varian model INOVA 500 spectrometer (^1^H-NMR at 500 MHz and ^13^C-NMR at 125 MHz) in the indicated deuterated solvent. In the kinetic studies monitored by ^1^H-NMR, threaded and septum tubes were used to perform the reaction in a closed system with an inert atmosphere. For the product characterization, the spectra were recorded at 30 °C. However, for the mechanism study of imine reduction, spectra were acquired at temperatures below 30 °C.

### 3.2. General Procedure for the Synthesis of Imine ***1***

In an oven-dried two-neck flask, a mixture of activated 4 Å molecular sieve (35 g), acetophenone (10 mL, 85.73 mmol), and aniline (10.16 mL, 111.44 mmol) in dry CH_2_Cl_2_ (40 mL) was gently stirred at room temperature for 24 h under nitrogen atmosphere. The reaction mixture was filtered through paper filter. The filtrate was concentrated in vacuo, and the residue was distillated by distillation under reduced pressure. The product was obtained in the distillated fraction when the temperature was 175–180 °C, and it was solidified after cooling, giving a yellow solid [68].

### 3.3. General Procedure for the Synthesis of Piv-l-Pro

A solution of l-proline (10 g, 86.86 mmol) in 2 M NaOH (50 mL) was cooled to 0 °C in an ice-water bath and stirred magnetically. Pivaloyl chloride (10.7 mL, 86.86 mmol) and 2 M NaOH (40 mL) were added in several alternating portions over the course of 1 h. The reaction mixture was stirred at 0 °C during this time, and the pH was checked periodically in order to confirm that the solution remained strongly alkaline. After addition was finished, the solution was stirred at room temperature for 1 h and extracted with CH_2_Cl_2_. Then, the aqueous phase was acidified to pH 1–2 with 6 M HCl and extracted with CH_2_Cl_2_. The organic phase was dried over anhydrous MgSO_4_, filtered, and concentrated in vacuo to obtain the product as a white solid. The product was used without further purification [69].

### 3.4. General Procedure for the l-Proline-Organocatalysts Synthesis *(**3**–**16**)*

Ethyl chloroformate (1 equiv.) was added to a solution of protected-l-proline (1 equiv.) and triethylamine (1 equiv.) in dry THF at 0 °C under nitrogen atmosphere. The reaction mixture was stirred at 0 °C for 30 min. Then, a solution of amine (1 equiv.) in dry THF was added, and the reaction mixture was stirred at 0 °C for 3 h, and afterwards, at room temperature overnight. The mixture was filtered, and the volatiles were removed under reduced pressure. The residual reagents were removed with acid-base extraction, and the product was purified by crystallization or column chromatography [53].

### 3.5. General Procedure for the Asymmetric Reduction with HSiCl_3_

To a stirred solution of imine **1** (0.1 g, 0.512 mmol) and the corresponding catalyst (1–40 mol%, depending of the study) in dry CH_2_Cl_2_ (1 mL) at 0 °C and under nitrogen atmosphere, trichlorosilane (77 μL, 0.768 mmol) was added. The reaction mixture was stirred at 0 °C for 16 h. After, saturated NaHCO_3_ was added, and the product was extracted with CH_2_Cl_2_. The organic phase was washed with brine, dried over MgSO_4_, and concentrated in vacuo. Yield and enantiomeric excess were determined using the crude product. In all reactions of this work, (*S*)-enantiomer was the one obtained in excess [52].

### 3.6. General Procedure for Kinetic Studies

A solution of 0.66 M imine **1** and 0.066 M corresponding catalyst in CDCl_3_ (dried over anhydrous MgSO_4_) was prepared under nitrogen atmosphere in an NMR tube with septum. The solution was cooled at 0 °C, and trichlorosilane (1.5 equiv.) was added. ^1^H-NMR spectra were recorded at 30 °C for different times (5 min, 0.5 h, 1 h, 2 h, 3 h, 4 h, 6 h, 8 h, and 24 h). During the reaction time, the NMR tube was cooled at 0 °C under nitrogen atmosphere. When the kinetic study was finished, saturated NaHCO_3_ was added, and the product was extracted with CH_2_Cl_2_. The organic phase was washed with brine, dried over MgSO_4_, and concentrated in vacuo. Yield and enantiomeric excess were determined using the crude product.

### 3.7. General Procedure for the Asymmetric Reduction with HSiCl_3_ in Flow Process

Two lines were connected to a PFA tubular flow reactor (length reactor: 3 m, internal diameter: 0.02 inch, residence time: 2.9 h). In the first line, a solution of 0.70 M imine **1** and 0.07 M catalyst **16** in dry CH_2_Cl_2_ under nitrogen atmosphere was pumped (with a syringe pump) into the reactor with a flow rate of 5 μL/min. In the second line, a solution of 2.63 M HSiCl_3_ in dry CH_2_Cl_2_ under nitrogen atmosphere was pumped with a flow rate of 2 μL/min. After the system was stabilized at 0 °C in a cryocool, various samples of outgoing solution were collected over saturated NaHCO_3_, using an automatic fraction collector. The sample collection was every 71 min, and the process was carried out for more than 11 h. When the fraction collection was finished, every sample was extracted with CH_2_Cl_2_, washed with brine, dried over MgSO_4_, and concentrated in vacuo. Yield and enantiomeric excess were determined using the crude product.

## 4. Conclusions

A series of experimental and computational studies have been combined to shed light on the mechanism of the HSiCl_3_ reduction of imine **1** catalyzed by *N*-protected prolinamides to facilitate catalyst optimization. It is important to note that in this process, the uncatalyzed process is always present, and achieving a process in which the catalyzed rate is much higher than the uncatalyzed one is essential to obtain an efficient and enantioselective catalytic system. Besides, the step for RIC formation is also an important factor, as it requires extensive hydrogen bond breaking and costly conformational changes, leading to the formation of weak complexes most likely at low concentrations that are sensitive to the structure of the catalyst. Regarding the structure of the prolinamides, two main structural elements can be used for the optimization of the catalytic activity: the synthesis of prolinamines from aromatic amines containing donor groups in the aromatic ring and the use of Piv instead of Cbz *N*-protection, most likely because only one intramolecularly hydrogen bonded conformer is possible in this case. From the systems studied, catalyst **16** caused the catalyzed reaction to be 581 times faster than the uncatalyzed reaction and allowed us to carry out the model reaction with 86% *ee* using a 1 mol% catalyst, obtaining good results even for low reaction times. This catalyst allowed the enantioselective reduction to be carried out under continuous flow conditions.

## Figures and Tables

**Table 1:** Enantioselective reduction of ketimine **1** catalyzed by 40 mol% of organocatalyts **3**–**9** ^a^.

Entry	Catalyst	Conv. ^b^ (%)	Yield ^b^ (%)	*ee*^c^ (%)
1 ^d^	-	-	23	0
2	3	91	91	85
3	4	98	83	85
4	5	99	95	0
5	6	99	94	81
6	7	98	97	82
7	8	99	99	88
8	9	93	91	0

^a^ Reaction conditions: Concentration **1** = 0.512 M, 1.5 equiv. HSiCl_3_, CH_2_Cl_2_, 0 °C, 16 h. ^b^ Conversions and yields determined by ^1^H-NMR on the crude reaction mixture. ^c^ Enantiomeric excess determined by chiral HPLC (*S* configuration for the major enantiomer). ^d^ See reference [46]. (Reaction conditions: Concentration **1** = 0.2 M, 3 equiv. HSiCl_3_, CH_2_Cl_2_, room temperature, 20 h).

**Table 2:** Experimental rate constants obtained for catalysts **3**, **6**, and **8** using Equations (1) and (2).

Entry	Catalyst	k (M^−1^ h^−1^)	k_2_ (M^−2^ h^−1^)	k_2_/k_1_ (M^−1^)
1	- ^a^	0.00543 ^c^	-	-
2	**3** ^b^	0.04000	0.5238	96.5
3	**6** ^b^	0.01124	0.0880	16.2
4	**8** ^b^	0.03647	0.4703	86.6

^a^ Reduction of ketimine **1** at Concentration **1** = 0.66 M, 1.5 equiv. HSiCl_3_, CDCl_3_, 0 °C, 24 h. ^b^ Reduction of ketimine **1** at concentration **1** = 0.66 M, [catalyst] = 0.066 M, 1.5 equiv. HSiCl_3_, CDCl_3_, 0 °C, 24 h. ^c^ The observed kinetic constant corresponds to k_1_ in this case.

**Table 3:** Experimental rate constants obtained for catalysts **12**–**14** using Equations (1) and (2) ^a^.

Entry	Cat.	k (M^−1^ h^−1^)	k_2_ (M^−2^ h^−1^)	k_2_/k_1_ (M^−1^)	*ee*_exp_^b^ (%)
1	**12**	0.02617	0.3142	57.8	77
2	**13**	0.04326	0.5732	105.5	79
3	**14**	0.00591	0.0073	1.3	10

^a^ Reduction of ketimine **1** in CDCl_3_ at concentration **1** = 0.66 M, catalyst concentration = 0.066 M, 1.5 equiv. HSiCl_3_, 0 °C, 24 h. ^b^ Enantiomeric excess determined by chiral HPLC (*S* configuration for the major enantiomer).

**Table 4:** Enantioselective reduction of ketimine **1** with HSiCl_3_ catalyzed by **15** and **16** ^a^.

Entry	Cat.	mol% Cat. (%)	[Cat] (M)	Conv. ^b^ (%)	Yield ^b^ (%)	*ee*^c^ (%)	ACE
1	**15**	1	0.005	99	99	81	47.87
2	**15**	5	0.026	99	99	82	9.69
3	**15**	40	0.205	99	99	82	1.21
4	**16**	1	0.005	96	88	86	49.05
5	**16**	5	0.026	100	100	87	11.28
6	**16**	40	0.205	99	99	86	1.38

^a^ Reaction conditions: 1.5 equiv. HSiCl_3_, CH_2_Cl_2_, 0 °C, 16 h. ^b^ Conversions and yields determined by ^1^H-NMR on the crude reaction mixture. ^c^ Enantiomeric excess determined by chiral HPLC (*S* configuration for the major enantiomer).

## Data Availability

Data is contained within the article and Supplementary Materials.

## Supplementary Materials

Additional information of this research is included in Figures S1–S15 and Tables S1–S10, which are available online. Kinetic fitting data, computational calculations, synthetic procedures, and characterization are also included.

## Sample Availability

Samples of the compounds are available from the authors.
