# Supplementary material for: Rational Design of Simple Organocatalysts for the HSiCl3 Enantioselective Reduction of (E)-N-(1-Phenylethylidene)aniline
Source: Molecules. 2021 Nov 18;26(22):6963. doi: 10.3390/molecules26226963 (PMC8625272; doi:10.3390/molecules26226963)
Supplement: Supplementary file 1 [file molecules-26-06963-s001.zip › molecules-1462841-supplementary-final.pdf]

molecules

MDPI

Supplementary Materials

Rational Design of Simple Organocatalysts for the $HSiCl_3$ Enantioselective Reduction of (E)-*N*-(1-phenylethylidene)aniline

María Maciá [1], Raúl Porcar [1,2], Vicente Martí-Centelles [1,3], Eduardo García-Verdugo [1,*], M. Isabel Burguete [1] and Santiago V. Luis [1,*]

[1] Department of Inorganic and Organic Chemistry, University Jaume I, Av. Vicent Sos Baynat s/n, 12071 Castellón, Spain; maciam@uji.es (M.M.), martiv@uji.es (V.M-C.), burguete@uji.es (M.I.B.)

[2] Department of Organic and Bio-Organic Chemistry, Faculty of Science, UNED, Avenida de Esparta s/n, 28232 Las Rozas-Madrid, Spain; rporcar@ccia.uned.es (R.P)

[3] Interuniversity Research Institute of Molecular Recognition and Technological Development (IDM), Polytechnic University of Valencia, University of Valencia, Valencia 46022, Spain

* Correspondence: cepeda@uji.es (E.G-V.), luiss@uji.es (S.V.L.)

Table of contents

1. Supporting figures and tables S2
2. Kinetic fitting data S21
3. Computational calculations S30
4. Synthetic procedures and characterization S52
5. NMR spectra S58
6. References S72

1. Supporting figures and tables

Table S1. Asymmetric reduction with different amino acids (Reaction conditions: [**1**] = 0.512 M)

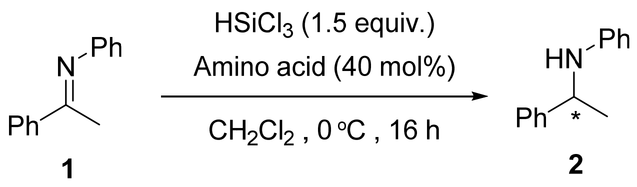

Entry	Amino acid	Conv.[a] (%)	Yield[a] (%)	*ee*[b] (%)
1[c]	**-**	-	23	0
2	**Cbz-L-Ala**	96	90	9
3	**Cbz-L-Phe**	99	91	4
4	**Cbz-L-His**	98	97	8
5	**Cbz-L-Trp**	97	91	6
6	**Cbz-L-Val**	98	85	0
7	**Cbz-L-Pro**	95	92	62
8	**L-Pro**	94	90	3
9	**Boc-L-Pro**	90	89	63
10	***N*-acetyl-L-Pro**	98	96	68
11	**Piv-L-Pro**	99	97	77

[a] Conversions and yields determined by ^{1}H-NMR on the crude of the reaction. [b] Enantiomeric excess determined by chiral HPLC (*S* configuration for the major enantiomer). [c] See Ref. [1]. (reaction conditions: [**1**] = 0.2 M, 3 equiv. $HSiCl_3$, CH_2Cl_2, room temperature, 20 h).

Table S2. Asymmetric reduction with different catalyst loadings (Reaction conditions: [**1**] = 0.512 M)

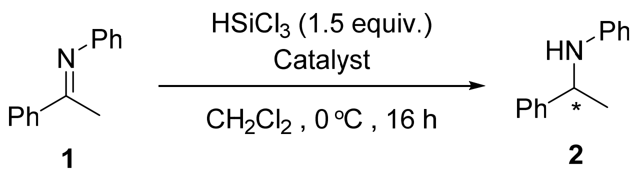

Entry	Cat.	mol% Cat. (%)	Conv.[a] (%)	Yield[a] (%)	*ee*[b] (%)	ACE
1	**3**	5	93	79	70	6.73
2	**3**	10	96	88	83	4.44
3	**3**	20	99	98	85	2.53
4	**3**	30	98	97	85	1.67
5	**3**	40	91	91	85	1.18
6	**4**	5	47	47	13	0.71
7	**4**	10	88	85	37	1.83
8	**4**	20	93	76	68	1.51
9	**4**	30	86	67	79	1.03
10	**4**	40	98	83	85	1.03
11	**6**	5	84	75	36	3.50
12	**6**	10	92	82	47	2.50
13	**6**	20	90	81	80	2.10
14	**6**	30	92	84	79	1.43
15	**6**	40	99	94	81	1.23
16	**8**	5	95	94	85	12.39
17	**8**	10	95	95	91	6.70
18	**8**	20	99	99	89	3.41
19	**8**	30	99	99	90	2.30
20	**8**	40	99	99	88	1.69

[a] Conversions and yields determined by ^{1}H-NMR on the crude of the reaction. [b] Enantiomeric excess determined by chiral HPLC (*S* configuration for the major enantiomer).

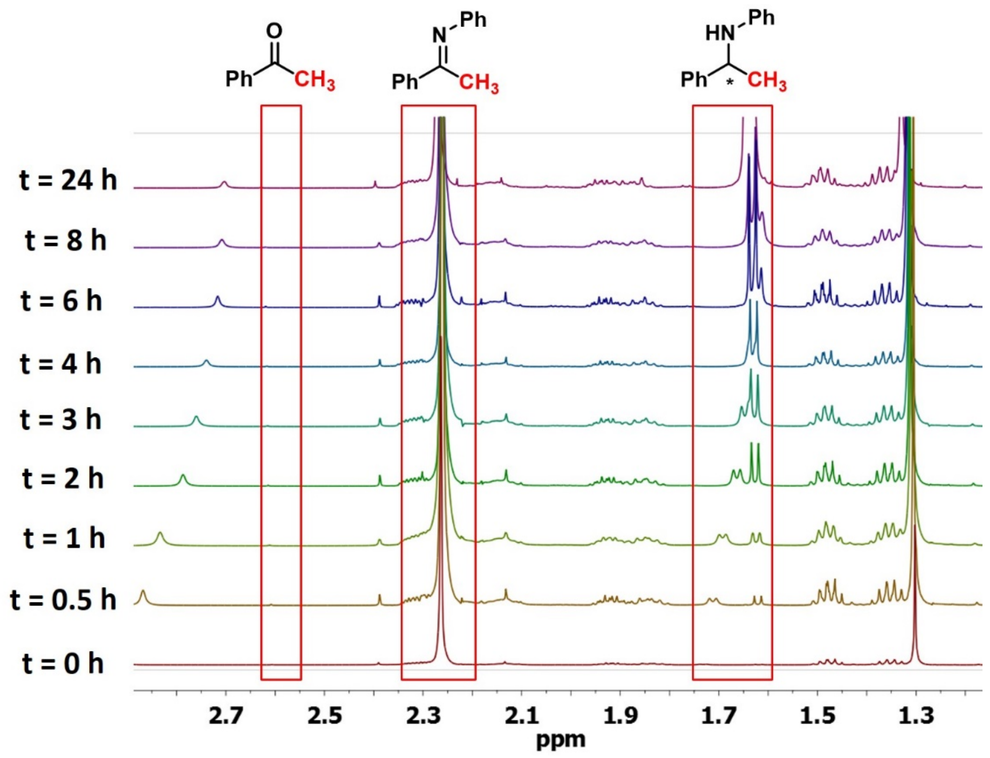

Figure S1. ^{1}H-NMR spectra obtained for the *in situ* reduction of ketimine **1** with $HSiCl_3$ (1.5 equiv.) in $CDCl_3$ at 0 °C, catalyzed by **8** (10 mol%). Spectra were acquired at 30 °C and 500 MHz.

$$[C]_{theoretical} = \frac{[A]_0[B]_0(1 - e^{([B]_0 - [A]_0)kt})}{[A]_0 - [B]_0 \cdot e^{([B]_0 - [A]_0)kt}}$$

Theoretical concentration of product according to the kinetic model.

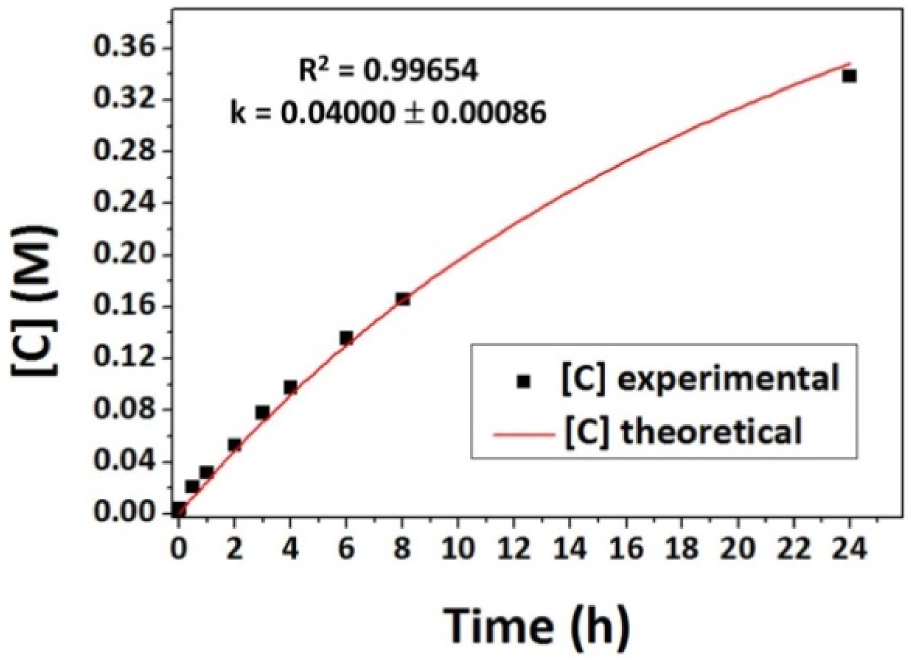

Figure S2. Fitting of the experimental data to the kinetic model for the reduction of the ketimine **1** with $HSiCl_3$ (1.5 equiv.) catalyzed by compound **3** (10 mol%) (^1H-NMR study in $CDCl_3$).

Table S3. Enantioselective reduction of the ketimine **1** at different concentrations of the catalyst **6** while maintaining constant the catalyst molar percentage (40 mol%)

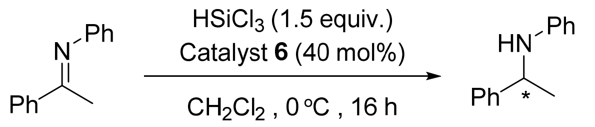

1 **2**

Entry	[Cat 6] (M)	[1] (M)	Conv.[a] (%)	Yield[a] (%)	*ee*[b] (%)
1	0.500	1.250	99	91	81
2	0.205	0.512	99	94	81
3	0.100	0.250	69	65	84
4	0.010	0.025	13	9	40

[a] Conversions and yields determined by ^{1}H-NMR on the crude of the reaction. [b] Enantiomeric excess determined by chiral HPLC (*S* configuration for the major enantiomer).

Table S4. Enantioselective reduction of the ketimine **1** at different substrate-catalyst ratios while maintaining constant the concentration of the catalyst **6** (0.205 M)

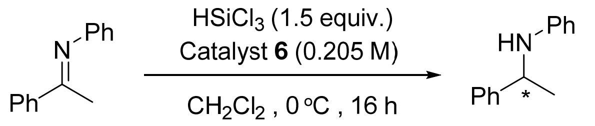

1 **2**

Entry	mol% (%)	[1] (M)	Conv.[a] (%)	Yield[a] (%)	*ee*[b] (%)
1	5	4.000	99	70	65
2	10	2.000	95	76	77
3	20	1.000	91	83	86
4	40	0.512	99	94	81

[a] Conversions and yields determined by ^{1}H-NMR on the crude of the reaction. [b] Enantiomeric excess determined by chiral HPLC (*S* configuration for the major enantiomer).

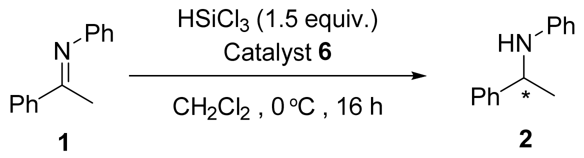

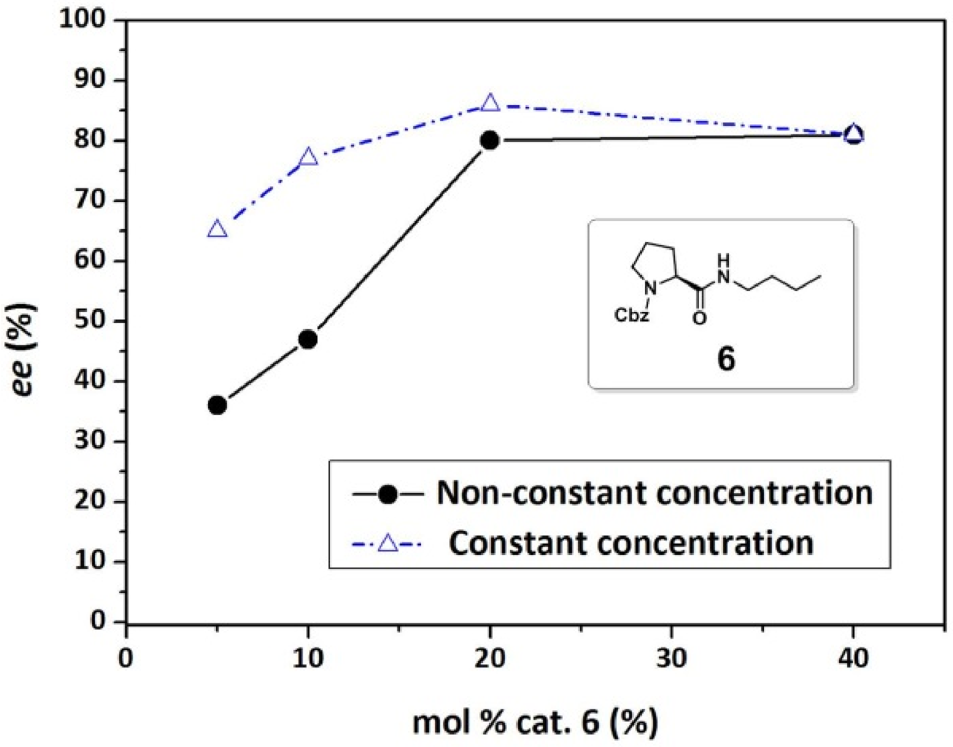

Figure S3. Variation of *ee* as a function of the molar percentage of catalyst **6** in the reduction of imine **1**. Black dots: non-constant concentration of catalyst; triangles: constant concentration of catalyst (0.205 M).

Table S5. Enantioselective reduction of the ketimine **1** with different concentrations of catalyst **8**, but keeping constant the catalyst : substrate ratio (5 mol%)

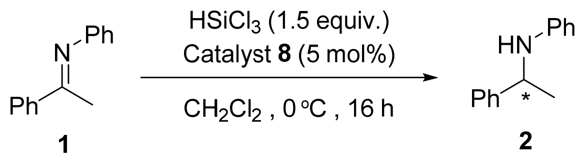

Entry	[Cat 8] (M)	[1] (M)	Conv.[a] (%)	Yield[a] (%)	*ee*[b] (%)
1	0.026	0.512	95	94	85
2	0.205	4.000	90	80	82

[a] Conversions and yields determined by ^{1}H-NMR on the crude of the reaction. [b] Enantiomeric excess determined by chiral HPLC (*S* configuration for the major enantiomer).

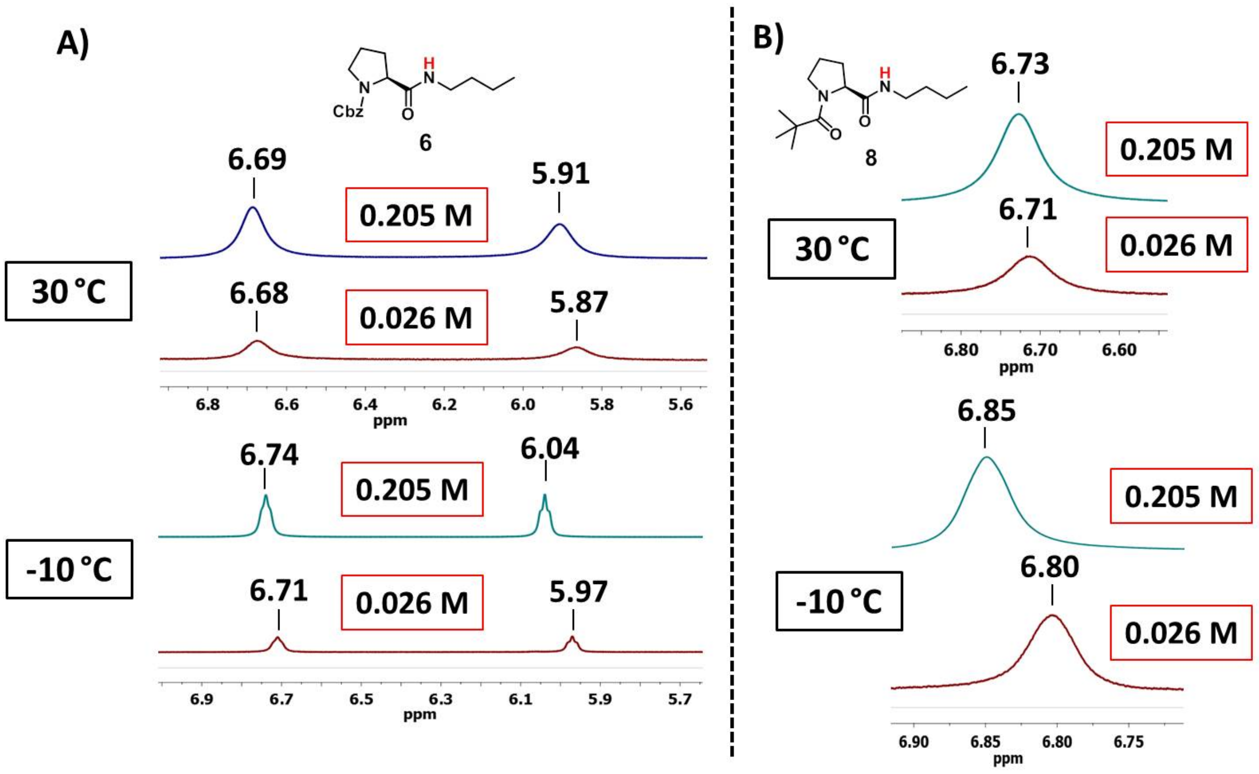

Figure S4. ^{1}H-RMN spectra for the amide signals at different concentrations and temperatures. A) Proton amide signals of catalyst **6**. B) Proton amide signals of catalyst **8**.

Table S6. Enantioselective reduction using catalysts **10-14**

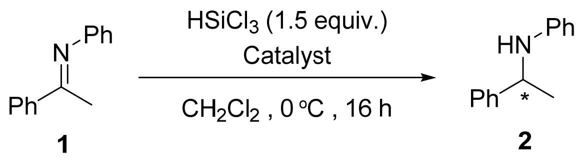

Entry	Cat	mol% Cat	[Cat] (M)	Conv.[a] (%)	Yield[a] (%)	*ee*[b] (%)	ACE
1	**10**	40	0.205	95	91	4	0.04
2	**11**	40	0.205	96	78	50	0.44
3	**12**	1	0.005	81	45	28	6.53
4	**12**	10	0.051	88	79	82	3.36
5	**12**	20	0.102	98	97	84	2.11
6	**12**	30	0.154	99	99	83	1.42
7	**12**	40	0.205	93	85	85	0.94
8	**13**	1	0.005	95	82	25	11.41
9	**13**	10	0.051	97	95	83	4.39
10	**13**	20	0.102	99	98	83	2.26
11	**13**	30	0.154	99	99	82	1.50
12	**13**	40	0.205	99	98	83	1.13
13	**14**	1	0.005	85	71	0	0.00
14	**14**	10	0.051	86	71	10	0.38
15	**14**	20	0.102	88	68	17	0.31
16	**14**	30	0.154	88	55	43	0.42
17	**14**	40	0.205	92	85	83	0.94

[a] Conversions and yields determined by ^{1}H-NMR on the crude reaction mixture. [b] Enantiomeric excess determined by chiral HPLC (*S* configuration for the major enantiomer).

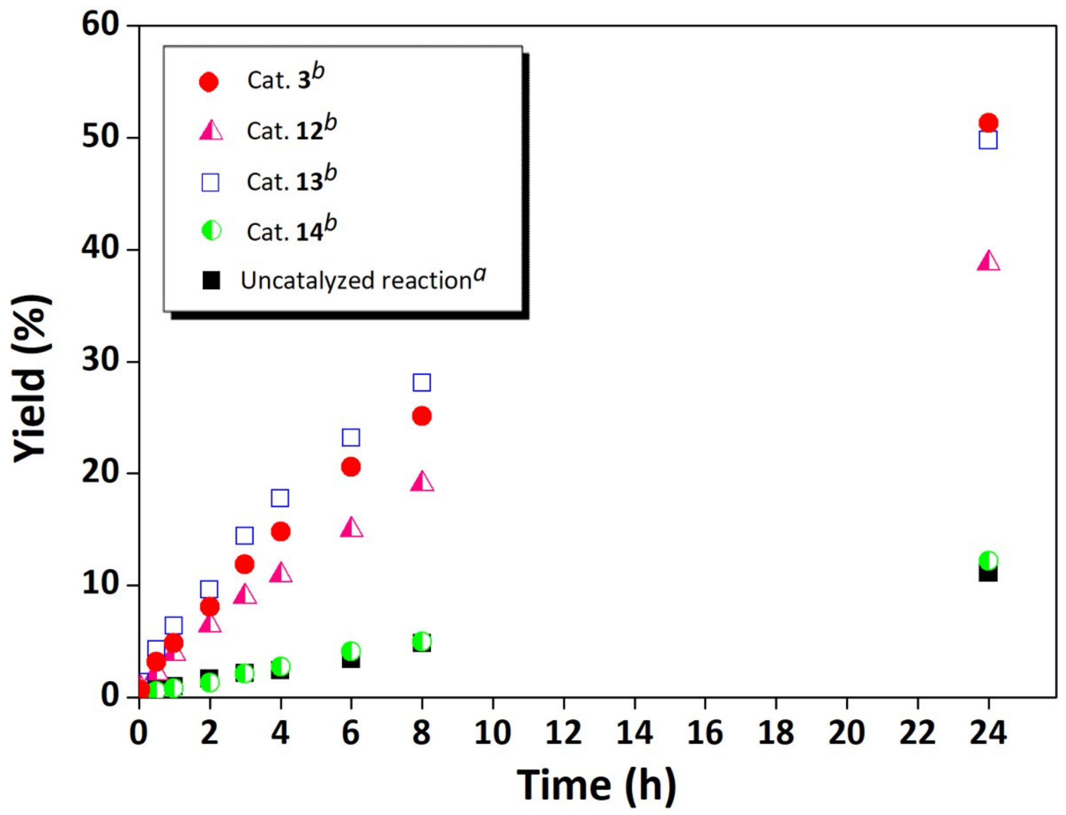

Figure S5. Kinetic curves obtained in the reduction of ketimine **1** in $CDCl_3$ using 10 mol% of aromatic prolinamides (**12-14**) with different aromatic substitutions. [a] [**1**] = 0.66 M, 1.5 equiv. $HSiCl_3$, 0 °C; [b] [**1**] = 0.66 M, [catalyst] = 0.066 M, 1.5 equiv. $HSiCl_3$, 0 °C.

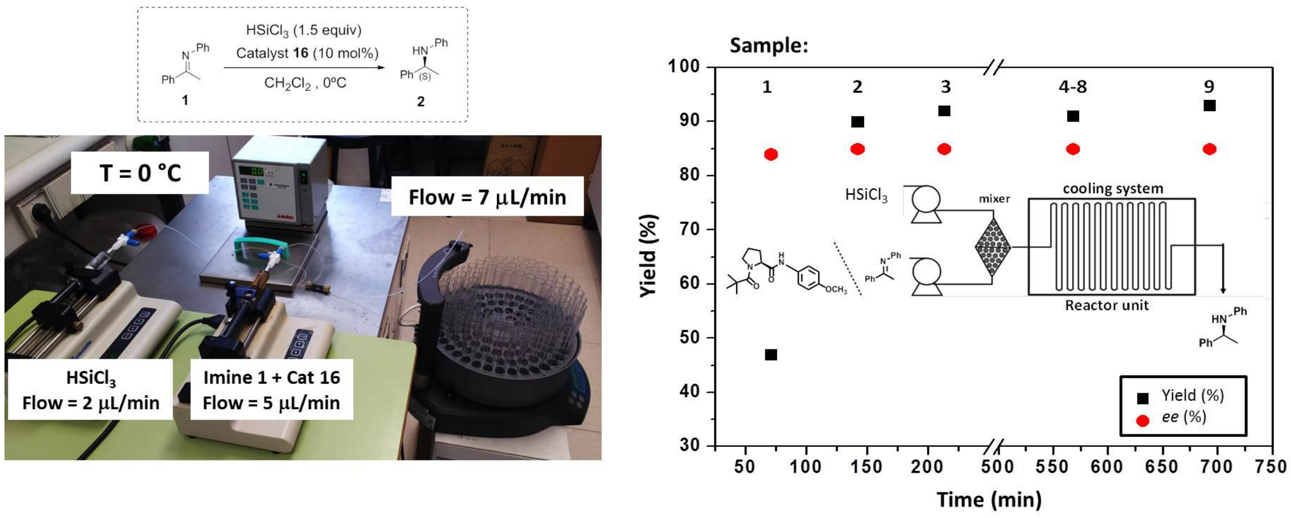

Figure S6. Set up of the system for the reduction of imine **1** under continuous flow and results of the analyzed samples during the flow process.

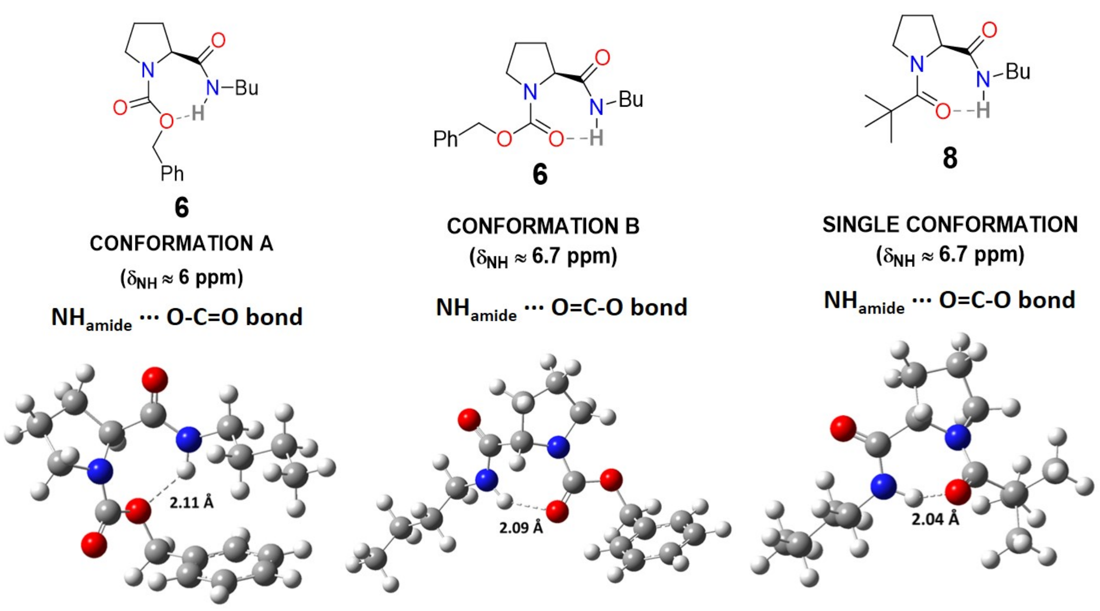

Figure S7. Possible conformations of catalysts **6** and **8**.

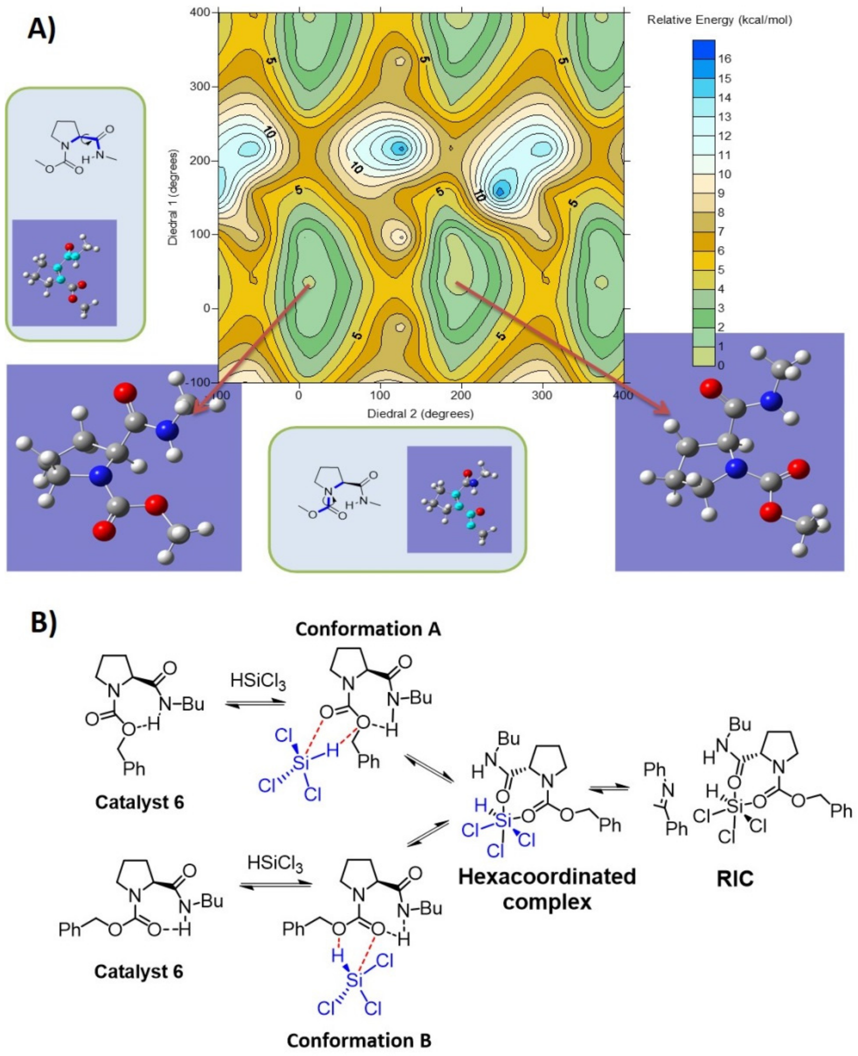

Figure S8. A) Conformational space for the simplified model of catalyst **6**. B) Evolution of catalyst **6** in the presence of $HSiCl_3$ and ketimine to form the RIC.

Table S7. Energies calculated for the most relevant states corresponding to a series of catalysts studied in this work, including *down* and *up* conformations.

Entry	Catalyst	Conf.	Energy of interaction complexes (Eh)						$\Delta E_{RIC\text{-}TS}$[a] (kcal/mol)		$ee_{calcd.}$[b] (%)	$\bar{ee}_{calcd.}$ (%)
			Enantiomer *R*			Enantiomer *S*						
			RIC	TS	PIC	RIC	TS	PIC	(*R*)	(*S*)		
1	Uncata-lyzed	-	-0.097	-0.051	-0.139	-0.097	-0.051	-0.139	29.38	29.38	0	0
2	**3**	*down*	-0.241	-0.212	-0.262	-0.241	-0.214	-0.267	17.75	17.23	44	32
		up	-0.240	-0.211	-0.262	-0.241	-0.213	-0.267	17.80	17.58	20	
3	**5**	*down*	-0.291	-0.213	-0.298	n.c	n.c	n.c	48.87	n.c	n.c	n.c
		up	n.c	n.c	n.c	n.c	n.c	n.c	n.c	n.c	n.c	
4	**6**	*down*	-0.315	-0.279	-0.330	-0.315	-0.279	-0.336	22.68	22.19	42	70
		up	-0.315	-0.278	-0.330	-0.314	-0.281	-0.336	22.85	20.57	97	
5	**12**	*down*	-0.280	-0.250	-0.300	-0.281	-0.252	-0.300	18.54	18.17	33	57
		up	-0.280	-0.249	n.c	-0.280	-0.251	n.c	19.17	17.99	80	
6	**13**	*down*	-0.306	-0.275	-0.325	-0.305	-0.277	-0.330	19.04	17.80	82	66
		up	-0.305	-0.274	-0.325	-0.306	-0.276	-0.330	19.30	18.70	50	
7	**14**	*down*	-0.246	-0.224	-0.269	-0.245	-0.226	-0.278	13.67	11.97	92	80
		up	-0.245	-0.223	-0.272	-0.245	-0.225	-0.267	13.33	12.44	68	

8	**16**	*down*	-0.310	-0.279	-0.331	-0.309	-0.281	-0.338	19.34	18.10	81	46
		up	-0.311	-0.280	n.c.	-0.312	-0.282	n.c.	19.04	18.91	11	

[a] Eh units were converted in kcal/mol units multiplying the ΔE_{RIC-TS} by 627.509.

[b] Enantiomeric excess calculated using eq 4.

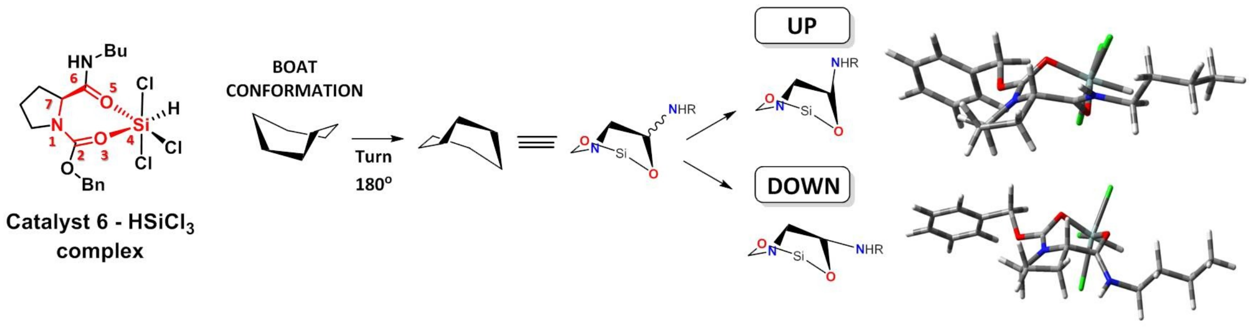

Figure S9. Representation of the two possible boat conformations (*up* and *down*) for the catalyst **6**-$HSiCl_3$ complex.

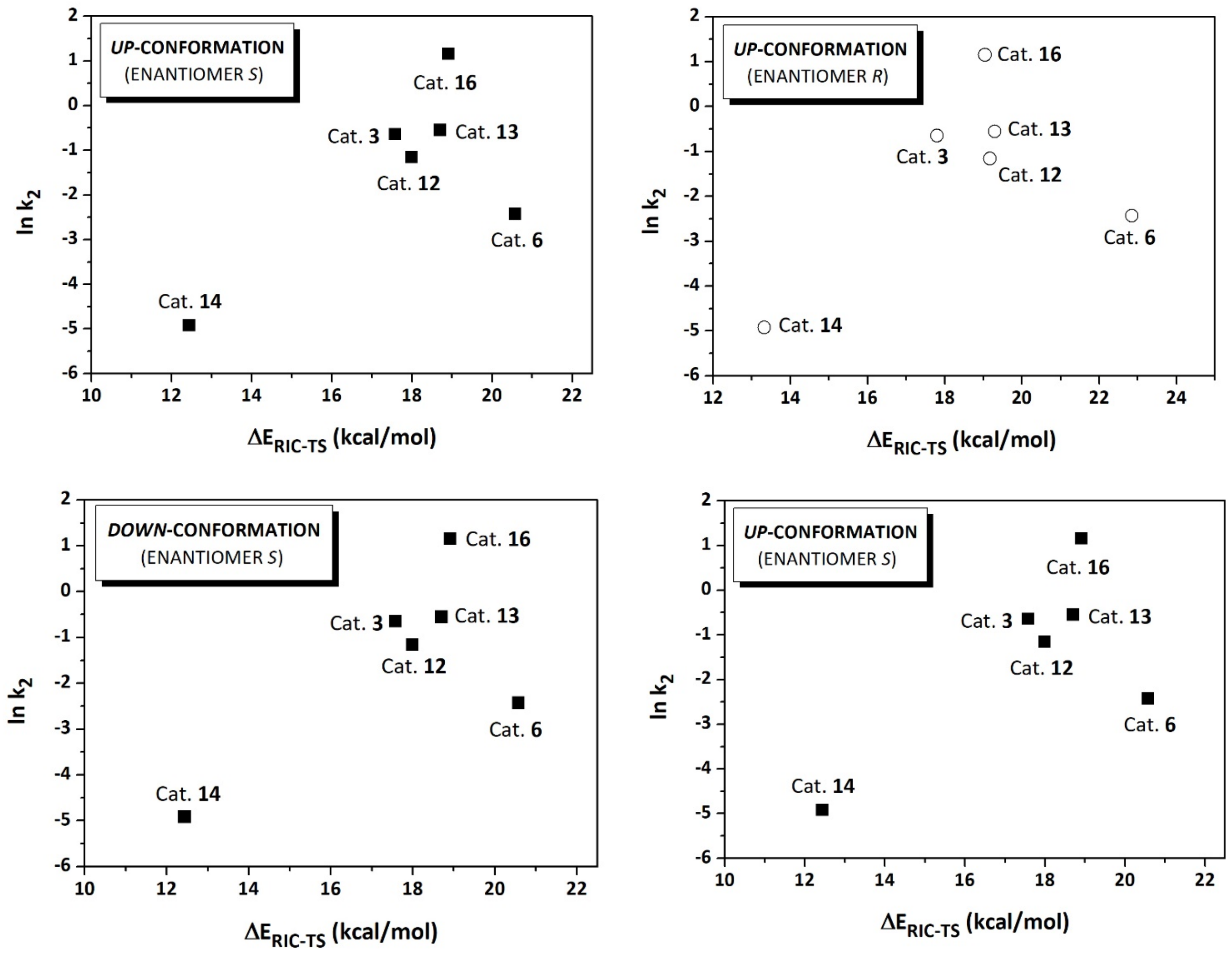

Figure S10. Ln k_2 (experimental) *vs.* $\Delta E_{RIC\text{-}TS}$ (theoretical) for the *S* and *R* enantiomers, both for the *up* and *down* conformations of the catalysts studied.

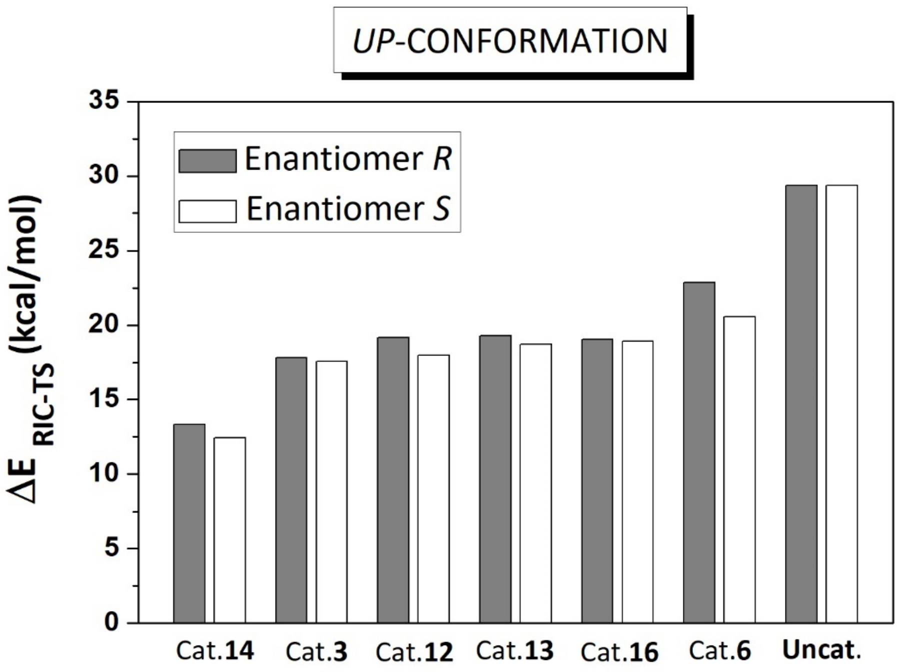
UP-CONFORMATION
Enantiomer *R*
Enantiomer *S*
ΔE RIC-TS (kcal/mol)
0
5
10
15
20
25
30
35
Cat.**14**
Cat.**3**
Cat.**12**
Cat.**13**
Cat.**16**
Cat.**6**
Uncat.

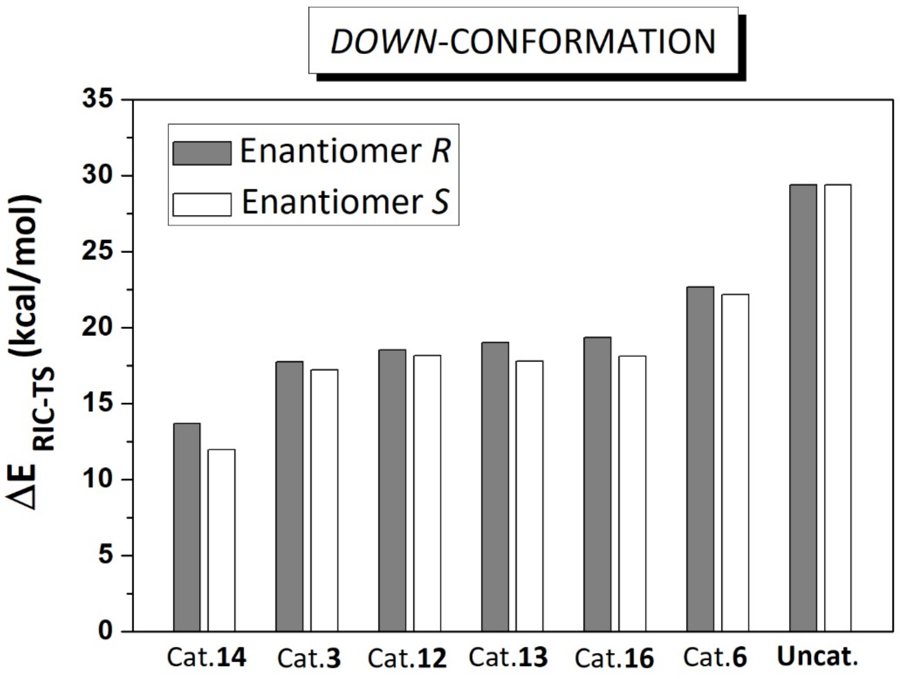

Figure S11. Energy difference between RIC and TS calculated for the different systems studied.

Table S8. Comparison of the enantioselectivity calculated by computational methods with the experimental values

Entry	Cat.	*ee*calcd.[a] (%) (*down*)	*ee*calcd.[a] (%) (*up*)	$\overline{ee}$ calcd.[b] (%)	*ee*exp.[c] (%)
1	**3**	44	20	32	76
2	**6**	42	97	70	63
3	**12**	33	80	57	77
4	**13**	82	50	66	79
5	**14**	92	68	80	10
6	**16**	81	11	46	88

[a] Values obtained by eq 4 using $\Delta E_{RIC\text{-}TS}$ (*R*) and $\Delta E_{RIC\text{-}TS}$ (*S*).

[b] Mean value obtained from enantioselectivity values for *up* and *down* conformations.

[c] Experimental enantioselectivity from the kinetic studies using 10 mol% of catalyst ([cat] = 0.205 M).

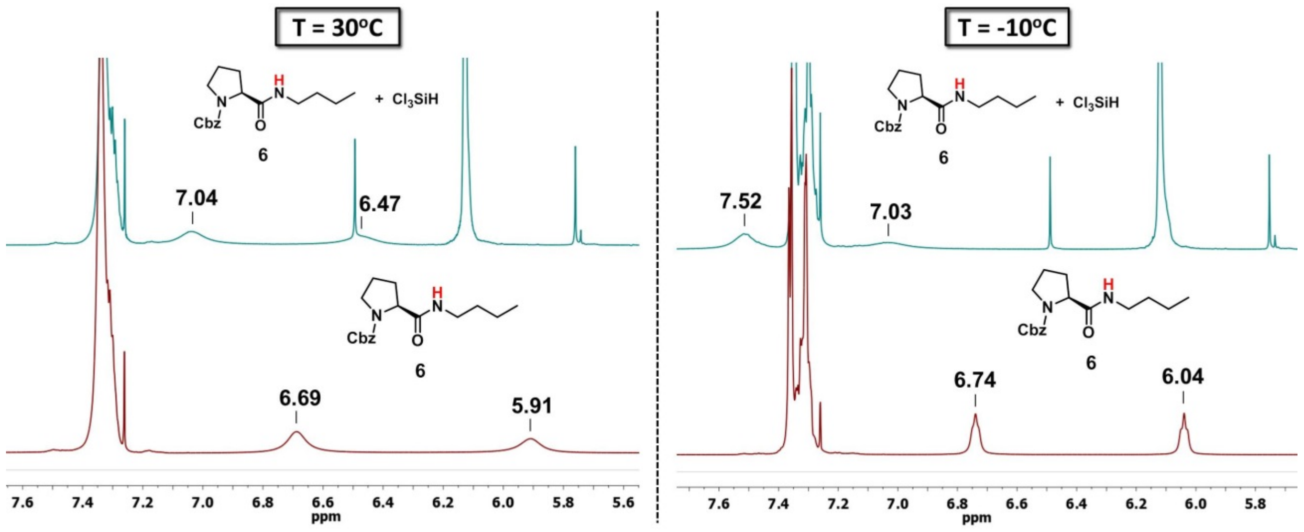

Figure S12. Comparison of ^{1}H-NMR spectra for catalyst **6** in the presence and absence of $HSiCl_3$ at different temperatures ([**6**] = 0.205 M, 1.5 eq $HSiCl_3$, $CDCl_3$, 500 MHz).

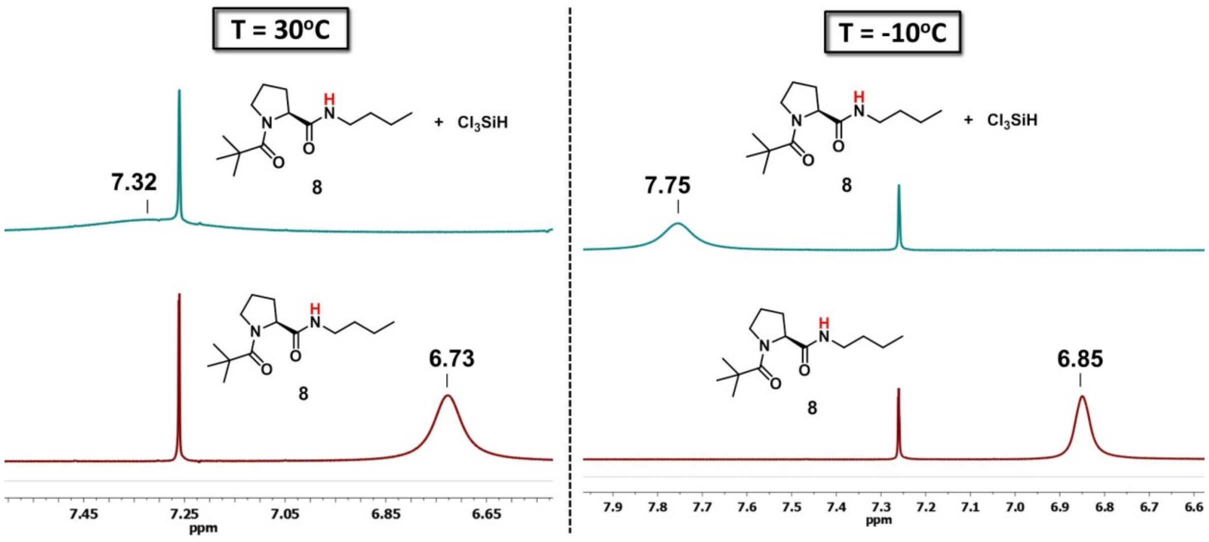

Figure S13. Comparison of ^{1}H-NMR spectra for catalyst **8** in the presence and absence of $HSiCl_3$ at different temperatures ([**8**] = 0.205 M, 1.5 eq $HSiCl_3$, $CDCl_3$, 500 MHz).

Table S9. Hydrogen bond distances and energies calculated for the optimized structures of the two most favorable conformations of the catalysts studied.

		**Structure with NH ··· O=C-O bond** 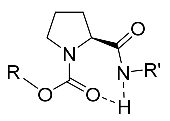		**Structure with NH ··· O-C=O bond** 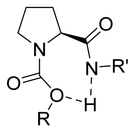	
**Entry**	**Cat.**	**d_{bond} (Å)**	**E (kcal/mol)**	**d_{bond} (Å)**	**E (kcal/mol)**
1	**3**	2.03	-87.38	2.14	-87.74
2	**6**	2.09	-131.93	2.11	-132.85
3	**12**	2.03	-111.69	2.14	-111.47
4	**13**	2.03	-126.75	2.13	-127.25
5	**14**	1.98	-95.07	2.09	-95.50
6	**16**	1.93	-128.86	no bond	--

Table S10. Calculated values for ΔE_{TOT}, δΔE_{TOT} and the expected enantiomeric excesses and comparison with the experimental enantioselectivity.

**Catalyst**	**Conf.**	**ΔETOT (kcal/mol)**		**δΔETOT (kcal/mol)**	***ee*calcd.[a] (%)**	**$\overline{ee}$ calcd.[b] (%)**	***ee* exp[c] (%)**
		**(*R*)**	**(*S*)**				
**3**	*down*	5.95	4.90	-1.05	75	81	76
	up	6.62	5.20	-1.42	86		
**6**	*down*	9.14	8.80	-0.34	31	61	78
	up	9.35	7.71	-1.63	91		
**12**	*down*	6.22	5.16	-1.06	75	81	77
	up	6.89	5.44	-1.46	87		
**13**	*down*	5.84	4.82	-1.02	74	81	79
	up	6.66	5.19	-1.47	88		
**14**	*down*	6.05	4.99	-1.06	75	77	10
	up	6.64	5.48	-1.16	79		
**16**	*down*	5.36	4.40	-- [d]	-- [d]	82	88
	up	4.57	3.31	-1.27	82		

[a] Values obtained by applying eq 5 using δΔE_{TOT}.

[b] Mean obtained with enantioselectivity values for *up* and *down* conformations.

[c] Experimental enantioselectivity of kinetic study using a 10 mol% catalyst ([cat] = 0.205 M).

[d] *Down*-conformation was discarded because ^{1}H-NMR spectra of catalyst **16** showed only one conformation and this conformation was higher in energy than *up*-conformation.

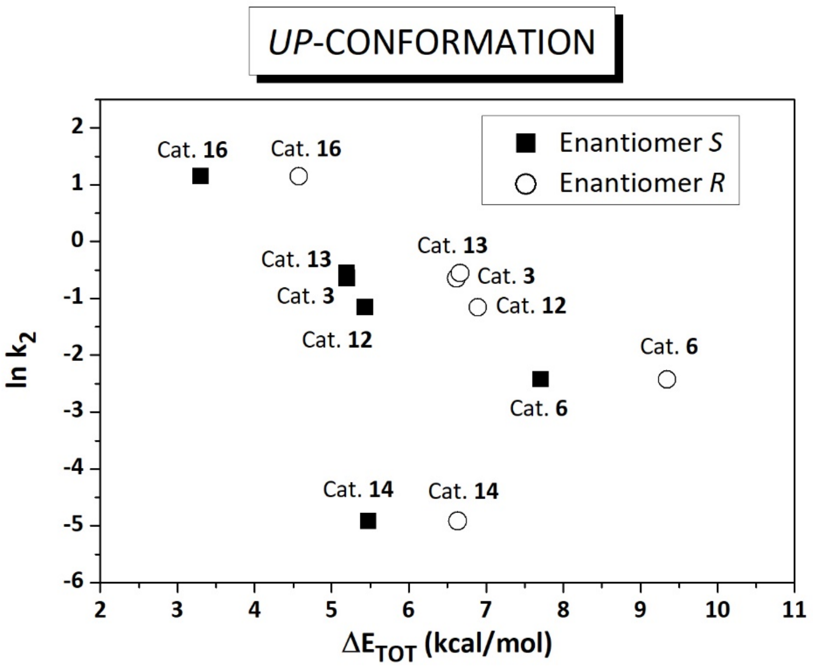
UP-CONFORMATION
Enantiomer S
Enantiomer R
Cat. 16
Cat. 16
Cat. 13
Cat. 3
Cat. 12
Cat. 13
Cat. 3
Cat. 12
Cat. 6
Cat. 6
Cat. 14
Cat. 14
ln k2
ΔETOT (kcal/mol)

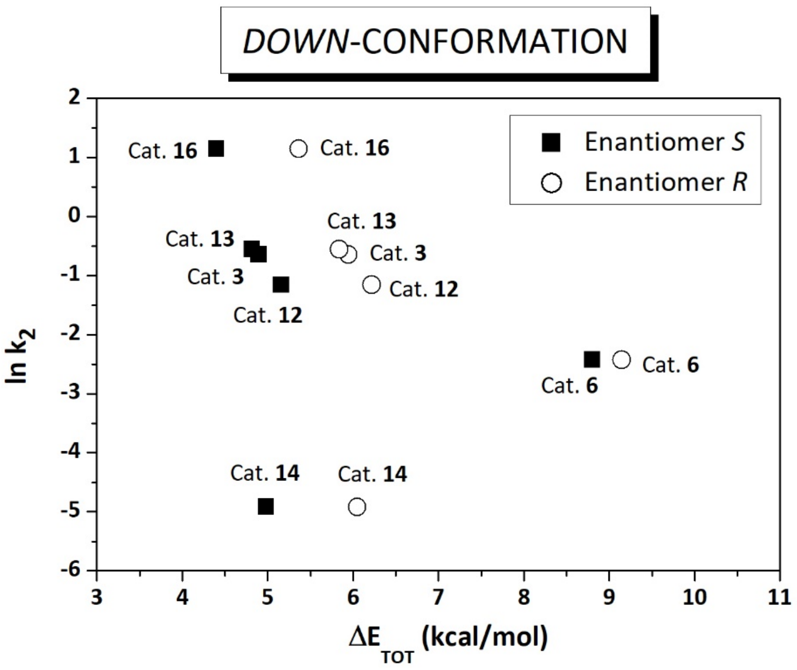

Figure S14. Representation of ln k_2 (experimental data) *vs*. ΔE_{TOT} (calculated data) for the *up* and *down* conformations.

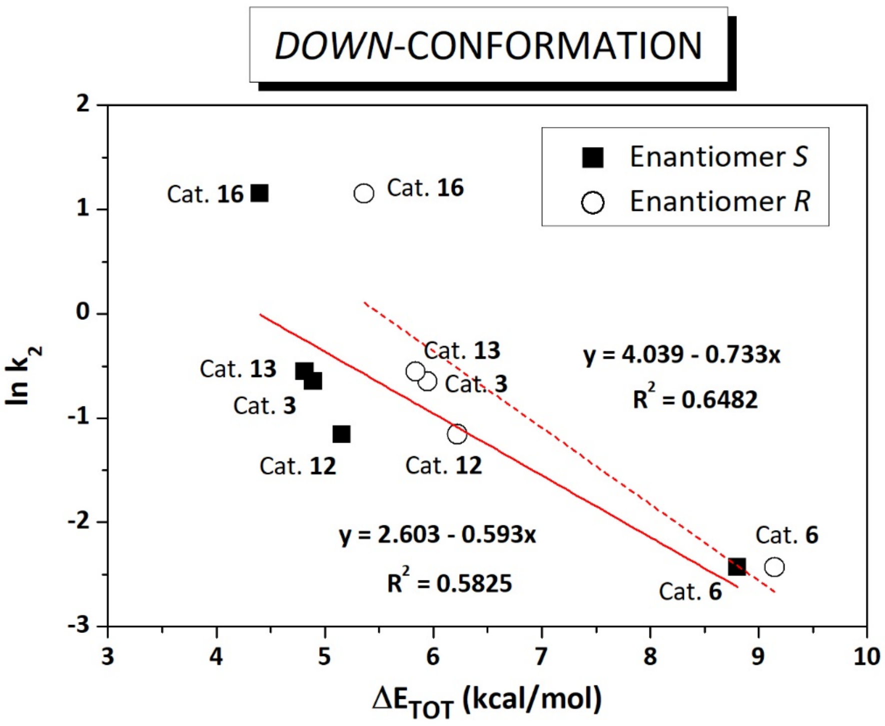

Figure S15. Representation of ln k2 (experimental data) *vs*. ΔETOT (theoretical data) for the *down* conformation.

1. Kinetic fitting data

Uncatalyzed reaction: $A + B \xrightarrow{k_1} C$
(Second-order reaction)

Catalyzed reaction: $A + B + D \xrightarrow{k_2} C + D$
(Third-order reaction)

A: Imine
B: $HSiCl_3$
C: Amine
D: Catalyst

Equations used in the calculations of ^{1}H-NMR kinetic studies:

Experimental concentrations: $[C]_{experimental} = \frac{[A]_0 \cdot \text{Yield (\%)}}{100}$ $[A]_0 = [A] + [C] \rightarrow [A] = [A]_0 - [C]$ $[B]_0 = [B] + [C] \rightarrow [B] = [B]_0 - [C]$ $[D]_0 = [D] = \text{constant}$	*Calculated concentration*: $[C]_{calculated} = \frac{[A]_0[B]_0(1 - e^{([B]_0 - [A]_0)kt})}{[A]_0 - [B]_0 \cdot e^{([B]_0 - [A]_0)kt}}$

$$\text{Yield (\%)} = \frac{\text{Integration } CH_3 \text{ amine}}{\text{Integration } CH_3 \text{ amine} + \text{Integration } CH_3 \text{ imine} + \text{Integration } CH_3 \text{ acetophenone}} \cdot 100$$

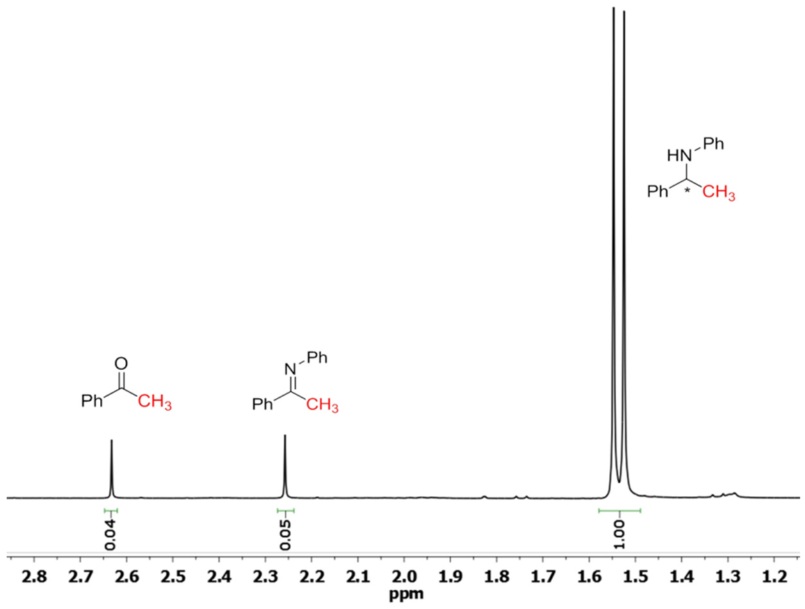

<u>Uncatalyzed reaction</u>:

Experimental concentrations for each reaction time, based on ^{1}H-NMR data:

t (h)	Yield (%)	[A] (M)	[B] (M)	[C] (M)
0	0.00	0.660	0.990	0.000
0.033	0.39	0.657	0.987	0.003
0.5	0.76	0.655	0.985	0.005
1	0.97	0.654	0.984	0.006
2	1.62	0.649	0.979	0.011
3	2.10	0.646	0.976	0.014
4	2.39	0.644	0.974	0.016
6	3.37	0.638	0.968	0.022
8	4.80	0.628	0.958	0.032
24	11.11	0.587	0.917	0.073

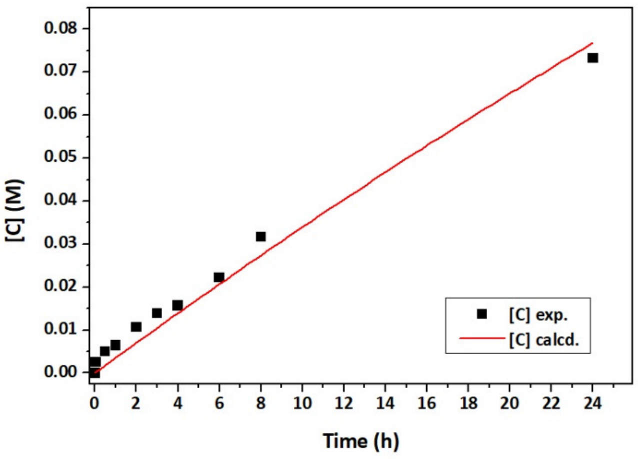

Equation	c=a0*b0*(1-exp((b0-a0)*k*t))/(a0-b0*exp((b0-a0)*k*t))		
Adj. R-Squar	0.97962		
		Value	Standard Error
D	a0	0.66	0
D	b0	0.99	0
D	**k**	**0.00543**	**2.11541E-4**

k_1 = 0.00543

Catalyzed reaction by 3:

Experimental concentrations for each reaction time, based on ^{1}H-NMR data:

t (h)	Yield (%)	[A] (M)	[B] (M)	[C] (M)	[D] (M)
0	0.00	0.660	0.990	0.000	0.066
0.033	0.63	0.656	0.986	0.004	0.066
0.5	3.16	0.639	0.969	0.021	0.066
1	4.80	0.628	0.958	0.032	0.066
2	8.05	0.607	0.937	0.053	0.066
3	11.84	0.582	0.912	0.078	0.066
4	14.78	0.562	0.892	0.098	0.066
6	20.55	0.524	0.854	0.136	0.066
8	25.13	0.494	0.824	0.166	0.066
24	51.28	0.322	0.652	0.338	0.066

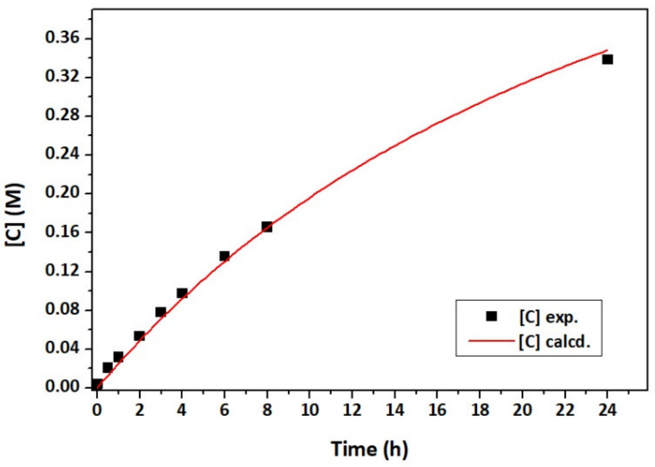

Equation	c=a0*b0*(1-exp((b0-a0)*k*t))/(a0-b0*exp((b0-a0)*k*t))		
Adj. R-Squar	0.99654		
		Value	Standard Error
D	a0	0.66	0
D	b0	0.99	0
D	**k**	**0.04**	**8.56996E-4**

Calculation of k_2:

$$\left.\begin{array}{c} k = 0.04 \\ k_1 = 0.00543 \\ [D] = [D]_0 = 0.066 \end{array}\right\} \quad k_2 = \frac{k - k_1}{[D]} = 0.5238$$

Catalyzed reaction by 6:

Experimental concentrations for each reaction time, based on ^{1}H-NMR data:

t (h)	Yield (%)	[A] (M)	[B] (M)	[C] (M)	[D] (M)
0	0.00	0.660	0.990	0.000	0.066
0.033	0.79	0.655	0.985	0.005	0.066
0.5	1.73	0.649	0.979	0.011	0.066
1	2.60	0.643	0.973	0.017	0.066
2	3.76	0.635	0.965	0.025	0.066
3	4.79	0.628	0.958	0.032	0.066
4	5.62	0.623	0.953	0.037	0.066
6	7.39	0.611	0.941	0.049	0.066
8	8.95	0.601	0.931	0.059	0.066
24	20.29	0.526	0.856	0.134	0.066

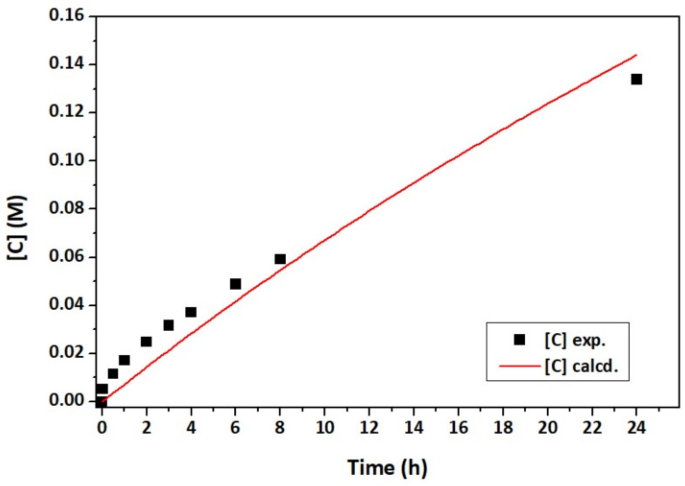

Equation	c=a0*b0*(1-exp((b0-a0)*k*t))/(a0-b0*exp((b0-a0)*k*t))		
Adj. R-Sq	0.9528		
		Value	Standard Error
D	a0	0.66	0
D	b0	0.99	0
D	**k**	**0.01124**	**6.81478E-4**

Calculation of k2:

$$\left.\begin{array}{l} k = 0.01124 \\ k_1 = 0.00543 \\ [D] = [D]_0 = 0.066 \end{array}\right\} \quad k_2 = \frac{k - k_1}{[D]} = 0.0880$$

Catalyzed reaction by 8:

Experimental concentrations for each reaction time, based on ^{1}H-NMR data:

t (h)	Yield (%)	[A] (M)	[B] (M)	[C] (M)	[D] (M)
0	0.00	0.660	0.990	0.000	0.066
0.033	1.28	0.652	0.982	0.008	0.066
0.5	2.89	0.641	0.971	0.019	0.066
1	5.71	0.622	0.952	0.038	0.066
2	8.28	0.605	0.935	0.055	0.066
3	11.35	0.585	0.915	0.075	0.066
4	13.89	0.568	0.898	0.092	0.066
6	18.58	0.537	0.867	0.123	0.066
8	23.92	0.502	0.832	0.158	0.066
24	48.05	0.343	0.673	0.317	0.066

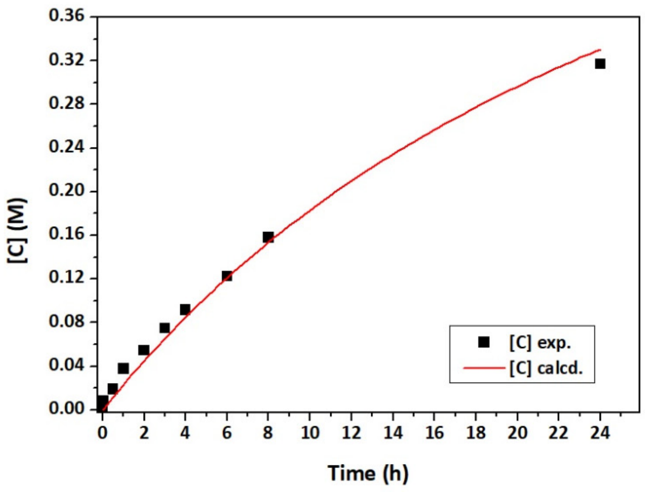

Equation	c=a0*b0*(1-exp((b0-a0)*k*t))/(a0-b0*exp((b0-a0)*k*t))		
Adj. R-Squ	0.9907		
		Value	Standard Error
D	a0	0.66	0
D	b0	0.99	0
D	**k**	**0.03647**	**0.00123**

Calculation of k_2:

$$\left.\begin{array}{r} k = 0.03647 \\ k_1 = 0.00543 \\ [D] = [D]_0 = 0.066 \end{array}\right\} \quad k_2 = \frac{k - k_1}{[D]} = 0.4703$$

<u>Catalyzed reaction by 12</u>:

Experimental concentrations for each reaction time, based on ^{1}H-NMR data:

t (h)	Yield (%)	[A] (M)	[B] (M)	[C] (M)	[D] (M)
0	0.00	0.660	0.990	0.000	0.066
0.033	0.74	0.655	0.985	0.005	0.066
0.5	2.16	0.646	0.976	0.014	0.066
1	3.87	0.634	0.964	0.026	0.066
2	6.44	0.617	0.947	0.043	0.066
3	9.00	0.601	0.931	0.059	0.066
4	10.93	0.588	0.918	0.072	0.066
6	14.98	0.561	0.891	0.099	0.066
8	19.04	0.534	0.864	0.126	0.066
24	38.78	0.404	0.734	0.256	0.066

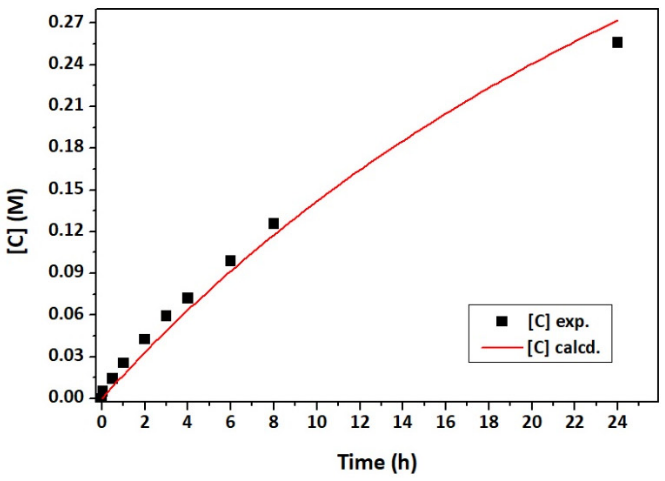

Equation	c=a0*b0*(1-exp((b0-a0)*k*t))/(a0-b0*exp((b0-a0)*k*t))		
Adj. R-Sq	0.98548		
		Value	Standard Error
D	a0	0.6666	0
D	b0	0.99	0
D	**k**	**0.02617**	**0.00104**

Calculation of k_2:

$$\left.\begin{array}{r} k = 0.02617 \\ k_1 = 0.00543 \\ [D] = [D]_0 = 0.066 \end{array}\right\} \quad k_2 = \frac{k - k_1}{[D]} = 0.3142$$

Catalyzed reaction by 13:

Experimental concentrations for each reaction time, based on ^{1}H-NMR data:

t (h)	Yield (%)	[A] (M)	[B] (M)	[C] (M)	[D] (M)
0	0.00	0.660	0.990	0.000	0.066
0.033	1.33	0.651	0.981	0.009	0.066
0.5	4.24	0.632	0.962	0.028	0.066
1	6.37	0.618	0.948	0.042	0.066
2	9.58	0.597	0.927	0.063	0.066
3	14.35	0.565	0.895	0.095	0.066
4	17.73	0.543	0.873	0.117	0.066
6	23.17	0.507	0.837	0.153	0.066
8	28.09	0.475	0.805	0.185	0.066
24	49.75	0.332	0.662	0.328	0.066

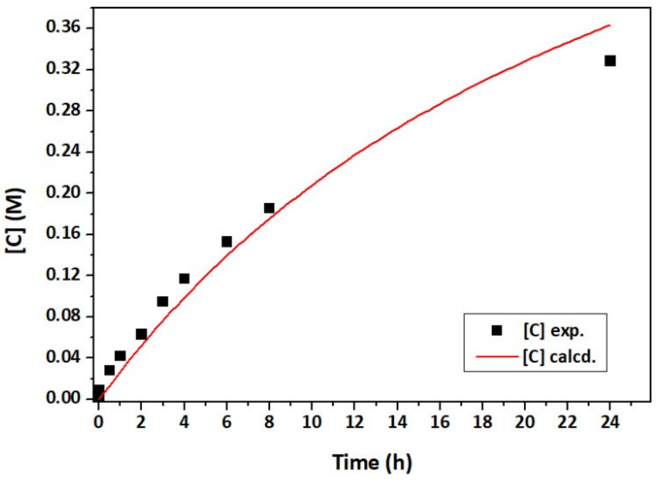

Equation	c=a0*b0*(1-exp((b0-a0)*k*t))/(a0-b0*exp((b0-a0)*k*t))		
Adj. R-Sq	0.96974		
		Value	Standard Error
D	a0	0.66	0
D	b0	0.99	0
D	**k**	**0.04326**	**0.0026**

Calculation of k_2:

$$\left.\begin{array}{r} k = 0.04326 \\ k_1 = 0.00543 \\ [D] = [D]_0 = 0.066 \end{array}\right\} \quad k_2 = \frac{k - k_1}{[D]} = 0.5732$$

Catalyzed reaction by 14:

Experimental concentrations for each reaction time, based on ^{1}H-NMR data:

t (h)	Yield (%)	[A] (M)	[B] (M)	[C] (M)	[D] (M)
0	0.00	0.660	0.990	0.000	0.066
0.033	0.22	0.659	0.989	0.001	0.066
0.5	0.50	0.657	0.987	0.003	0.066
1	0.78	0.655	0.985	0.005	0.066
2	1.27	0.652	0.982	0.008	0.066
3	2.10	0.646	0.976	0.014	0.066
4	2.70	0.642	0.972	0.018	0.066
6	4.07	0.633	0.963	0.027	0.066
8	4.94	0.627	0.957	0.033	0.066
24	12.20	0.580	0.910	0.080	0.066

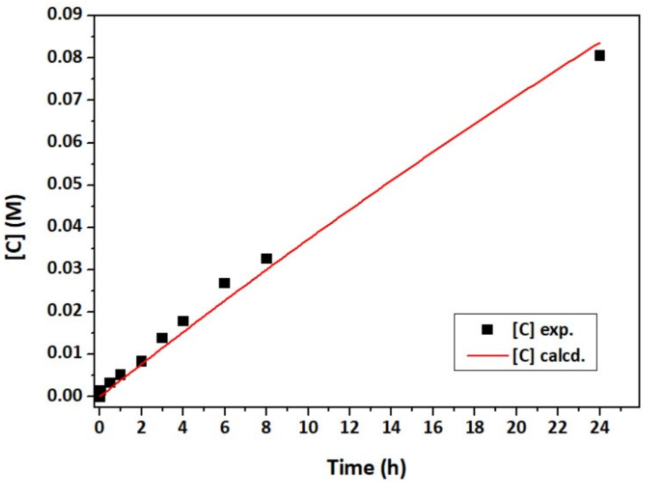

Equation	c=a0*b0*(1-exp((b0-a0)*k*t))/(a0-b0*exp((b0-a0)*k*t))		
Adj. R-Sq	0.9902		
		Value	Standard Error
D	a0	0.6666	0
D	b0	0.99	0
D	**k**	**0.00591**	**1.65068E-4**

Calculation of k_2:

$$\left.\begin{array}{r} k = 0.00591 \\ k_1 = 0.00543 \\ [D] = [D]_0 = 0.066 \end{array}\right\} \quad k_2 = \frac{k - k_1}{[D]} = 0.0073$$

Catalyzed reaction by 16:

Experimental concentrations for each reaction time, based on ^{1}H-NMR data:

t (h)	Rto (%)	[A] (M)	[B] (M)	[C] (M)	[D] (M)
0	0.00	0.660	0.990	0.000	0.066
0.033	2.88	0.641	0.971	0.019	0.066
0.5	14.72	0.563	0.893	0.097	0.066
1	21.53	0.518	0.848	0.142	0.066
2	33.44	0.439	0.769	0.221	0.066
3	42.37	0.380	0.710	0.280	0.066
4	49.50	0.333	0.663	0.327	0.066
6	60.24	0.262	0.592	0.398	0.066
8	66.67	0.220	0.550	0.440	0.066
24	87.95	0.080	0.410	0.580	0.066

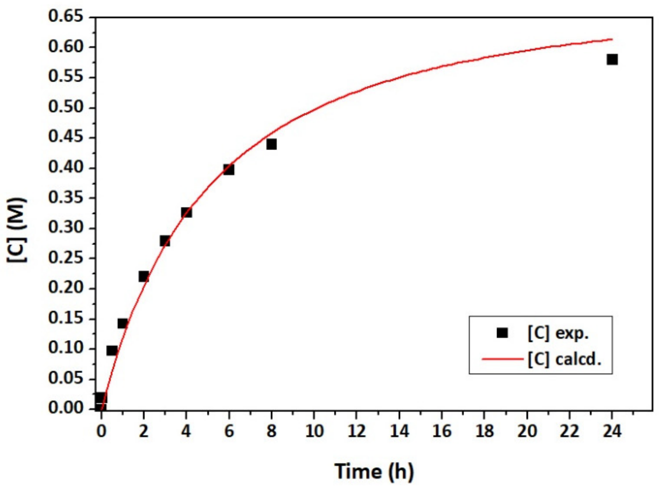

Equation	c=a0*b0*(1-exp((b0-a0)*k*t))/(a0-b0*exp((b0-a0)*k*t))		
Adj. R-Sq	0.98897		
		Value	Standard Error
D	a0	0.66	0
D	b0	0.99	0
D	**k**	**0.21395**	**0.01002**

Calculation of k_2:

$$\left.\begin{array}{c} k = 0.21395 \\ k_1 = 0.00543 \\ [D] = [D]_0 = 0.066 \end{array}\right\} \quad k_2 = \frac{k - k_1}{[D]} = 3.1594$$

2. Computational calculations

Computational modelling:

All structures where minimized by using the Gaussian 09 software [2] at the semiempirical PM6 level of theory in the gas phase. Normal mode of vibration analysis showed all positive values except for transition state structures, were one single negative eigenvalue was found, associated to the reaction path involving the breaking and forming of bonds. An intrinsic reaction coordinate calculation was performed for the transition state structures (TS) to confirm that the reactant interaction complexes (RIC) and the product interaction complexes (PIC). Further details of the different calculation performed are described in the manuscript main text.

The results of the most representative calculations in this work are shown below.

- Enantioselective reduction of the simplified imine using the simplified organocatalyst, obtaining the *R*-amine as product.

Reactants interaction complex (RIC):

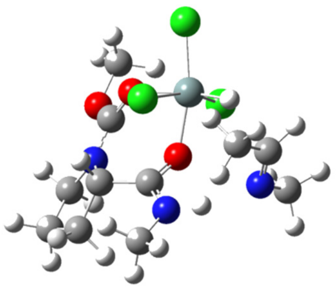

Transition state (TS):

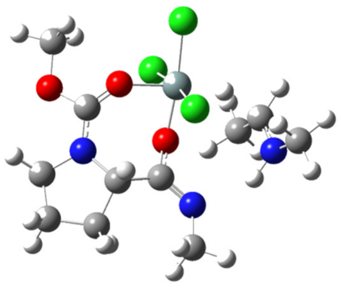

Products interaction complex (PIC):

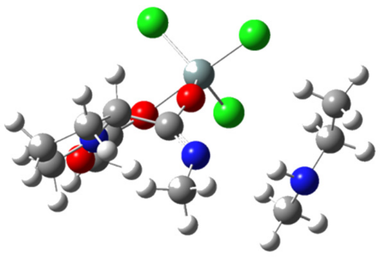

- Enantioselective reduction of the simplified imine using the simplified organocatalyst, obtaining the *S*-amine as product.

Reactants interaction complex (RIC):

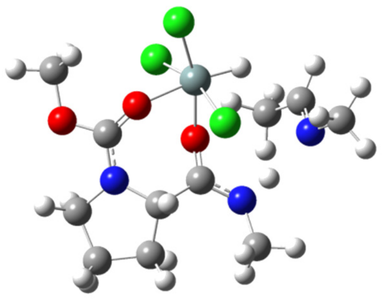

Transition state (TS):

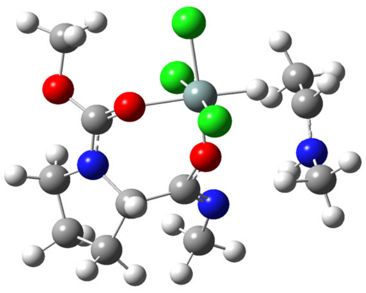

Products interaction complex (PIC):

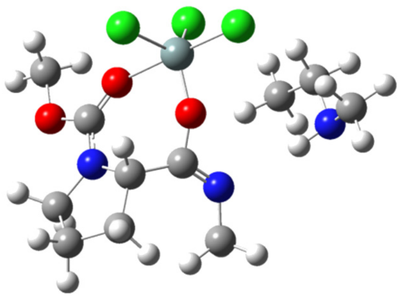

- Enantioselective reduction of the imine **1** using the organocatalyst **16**, obtaining the *R*-amine **2** as product.

Reactants interaction complex (RIC):

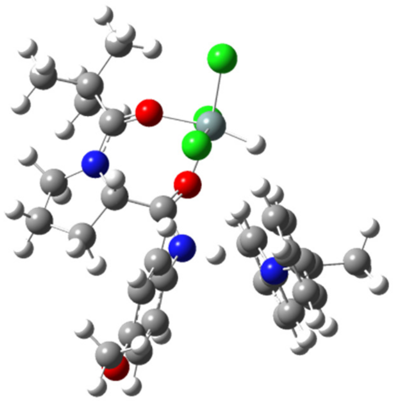

Transition state (TS):

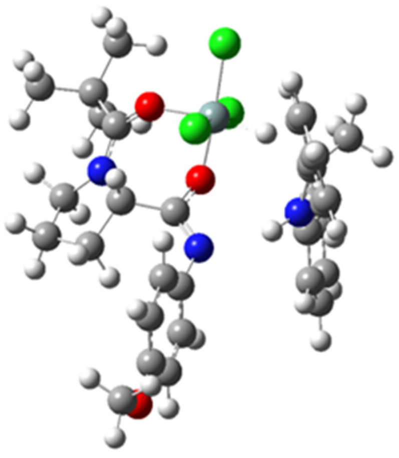

Products interaction complex (PIC):

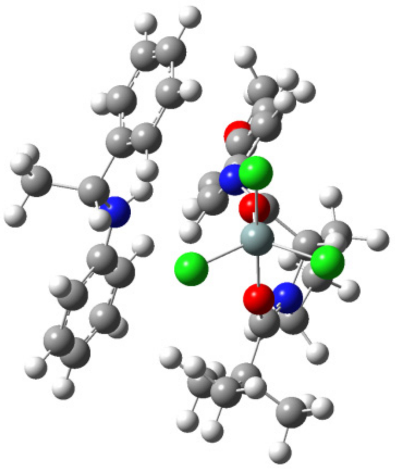

- Enantioselective reduction of the imine **1** using the organocatalyst **16**, obtaining the *S*-amine **2** as product.

Reactants interaction complex
(RIC):

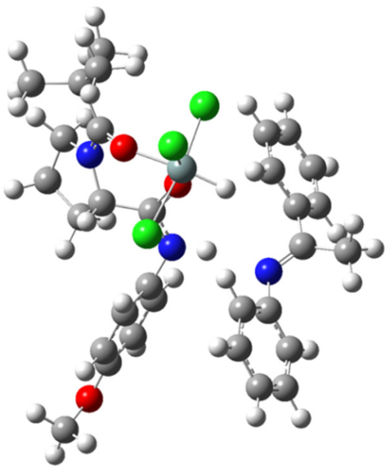

Transition state
(TS):

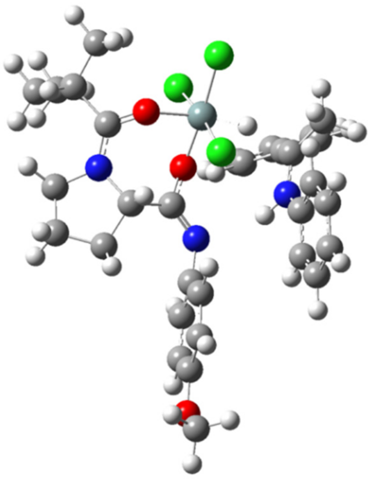

Products interaction complex
(PIC):

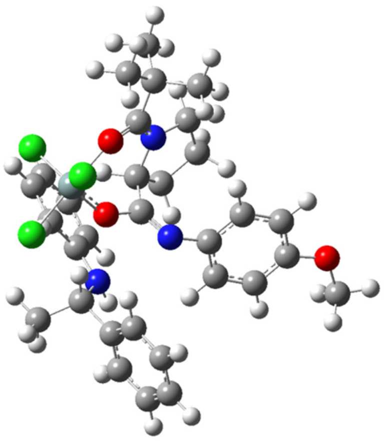

Cartesian coordinates calculations

The Cartesian coordinates of the most representative calculations in this work are shown below.

- Enantioselective reduction of the simplified imine using the simplified organocatalyst, obtaining the *R*-amine as product.

Reactants interaction complex (RIC):

43

Si	0.69200	1.28600	-0.18300
N	-2.18700	-0.96800	-0.79500
C	-1.67200	-1.51800	0.49200
C	-0.13900	-1.41100	0.60300
O	0.48800	-0.62900	-0.17600
C	-3.04200	-1.93800	-1.54500
C	-2.21200	-2.96500	0.52400
H	-1.41300	-3.69500	0.29000
C	-3.32500	-3.05800	-0.53600
H	-2.60100	-3.21900	1.52600
H	-4.32100	-2.92900	-0.07200
H	-3.33500	-4.04900	-1.02300
H	-2.49600	-2.30400	-2.44300
H	-3.96500	-1.42800	-1.89700
H	-2.08900	-0.89000	1.33800
C	-1.87200	0.27200	-1.29500
O	-1.15300	1.13000	-0.75400
Cl	-0.21200	1.10500	1.86200
N	0.61800	-2.13500	1.45800
Cl	1.19500	0.90100	-2.25700
N	3.36400	-1.19000	1.28600
C	3.69800	-0.15200	1.98300
Cl	0.41000	3.34100	-0.35500

H	2.05300	1.19700	0.32400
H	1.67300	-1.92100	1.48400
C	3.07900	0.06300	3.32600
H	3.77800	0.52800	4.03200
H	2.21000	0.74700	3.22400
H	2.69800	-0.85800	3.78300
C	3.87900	-1.35900	-0.07900
H	4.76600	-0.76100	-0.33700
H	4.11800	-2.42300	-0.23900
H	3.07500	-1.08000	-0.79900
C	0.11600	-3.04800	2.49700
H	-0.09100	-4.05300	2.08900
H	0.89100	-3.16400	3.28100
H	-0.78900	-2.66200	2.99700
C	-2.16900	1.83700	-3.07300
H	-2.22100	2.64900	-2.33600
H	-1.14700	1.74300	-3.46700
H	-2.92400	1.92400	-3.85800
O	-2.54600	0.56000	-2.44700
H	4.40300	0.63500	1.67000

Transition state (TS):

43

Si	0.57900	1.16000	-0.01400
N	-2.05900	-1.08300	-0.89600
C	-1.61300	-1.66700	0.40900
C	-0.08700	-1.57400	0.66200
O	0.51700	-0.64400	-0.05200
C	-2.83800	-2.04900	-1.73200
C	-2.13800	-3.11800	0.35600

H	-1.31400	-3.82700	0.14000
C	-3.18800	-3.18800	-0.76800
H	-2.57200	-3.41600	1.32600
H	-4.20800	-3.06300	-0.36000
H	-3.17100	-4.16900	-1.27400
H	-2.21100	-2.39500	-2.58400
H	-3.73500	-1.54700	-2.15500
H	-2.10300	-1.07600	1.23700
C	-1.78000	0.17500	-1.33300
O	-1.16300	1.08800	-0.72300
Cl	-0.32900	1.06600	1.97300
N	0.66500	-2.26200	1.47200
Cl	1.33800	0.98800	-2.04000
N	3.03400	-0.42500	2.15200
C	3.31100	0.27400	1.00500
Cl	0.47100	3.24000	-0.08300
H	2.02000	1.08000	0.58300
H	2.48700	-1.30800	2.06500
C	-2.04200	1.79000	-3.07800
H	-2.29000	2.57100	-2.34900
H	-0.97000	1.81500	-3.31900
H	-2.67700	1.82000	-3.96800
H	3.82200	1.25000	1.10300
C	3.60100	-0.53000	-0.22000
H	3.79400	0.10500	-1.10200
H	4.46800	-1.19100	-0.08200
H	2.74000	-1.17300	-0.51500
C	2.83700	0.30500	3.42100
H	1.87900	0.87100	3.43200
H	2.80800	-0.41300	4.25800
H	3.65600	1.01800	3.61200
C	0.17100	-3.35200	2.31800

H	0.08300	-4.30000	1.76200
H	0.93200	-3.50900	3.10700
H	-0.78400	-3.14100	2.82500
O	-2.37700	0.47000	-2.52900

Products interaction complex (PIC):

43

Si	0.04300	1.49200	0.49100
N	-2.21900	-1.19500	-0.79100
C	-2.08300	-1.29200	0.69600
C	-0.58300	-1.30800	1.04700
O	-0.16400	-0.02200	1.09900
C	-3.13500	-2.22900	-1.35800
C	-2.90400	-2.53300	1.08400
H	-2.25300	-3.39200	1.35500
C	-3.78300	-2.88900	-0.13100
H	-3.52100	-2.32800	1.97900
H	-4.81400	-2.51600	0.01500
H	-3.86400	-3.98200	-0.25900
H	-2.54400	-2.95200	-1.96300
H	-3.87800	-1.75400	-2.03400
H	-2.53500	-0.36900	1.16800
C	-1.63800	-0.20100	-1.53000
O	-0.86000	0.68900	-1.14600
Cl	0.98100	2.26300	2.11300
N	0.33200	-2.17300	1.28000
Cl	1.49300	1.81500	-0.89400
N	3.68700	-1.27500	1.06000
C	3.98700	0.07100	1.64900
Cl	-1.65600	2.61900	0.53600

H	3.48700	0.90500	1.10400
H	2.73500	-1.55900	1.33800
C	3.57000	0.10100	3.11900
H	4.00600	-0.73600	3.67500
H	3.89100	1.03100	3.59800
H	2.47800	0.03900	3.22500
C	3.75600	-1.23700	-0.42900
H	4.77700	-0.96900	-0.74400
H	3.54700	-2.24700	-0.81300
H	3.05500	-0.53400	-0.90500
C	0.17900	-3.62200	1.29300
H	0.78000	-4.04300	0.46200
H	0.63100	-4.00000	2.23300
H	-0.85100	-4.01600	1.21500
C	-1.35900	0.75200	-3.70600
H	-1.77900	1.72800	-3.44100
H	-0.27000	0.74900	-3.57300
H	-1.66100	0.42000	-4.70500
O	-1.96900	-0.27700	-2.86000
H	5.08300	0.23600	1.55700

- Enantioselective reduction of the simplified imine using the simplified organocatalyst, obtaining the *S*-amine as product.

Reactants interaction complex (RIC):

43

Si	0.45500	1.46800	0.04000
N	-2.08400	-1.05300	-0.93500
C	-1.63200	-1.61400	0.37100
C	-0.14500	-1.33200	0.65400
O	0.46500	-0.45800	-0.03700
C	-2.72100	-2.07300	-1.82300

C	-1.98500	-3.11500	0.27700
H	-1.08000	-3.72900	0.10700
C	-2.95500	-3.28200	-0.90800
H	-2.44700	-3.47200	1.21500
H	-4.00300	-3.31200	-0.55500
H	-2.77800	-4.23300	-1.44100
H	-2.04100	-2.30900	-2.67200
H	-3.66200	-1.66200	-2.24900
H	-2.20700	-1.09200	1.19600
C	-1.87200	0.24300	-1.33500
O	-1.32100	1.13800	-0.66700
Cl	-0.54900	1.10700	2.00200
N	0.59200	-1.99400	1.57400
Cl	1.12900	1.21800	-2.01600
N	3.05300	-0.60900	2.11000
C	3.80300	-0.22800	1.12600
Cl	-0.04000	3.48700	-0.10100
H	1.78600	1.51200	0.62600
H	1.59400	-1.63000	1.76200
C	-2.17600	1.87700	-3.05000
H	-2.29800	2.62800	-2.25700
H	-1.14500	1.88600	-3.42800
H	-2.92400	1.96900	-3.84200
H	4.46100	0.65500	1.12300
C	3.78000	-0.99600	-0.15500
H	3.00100	-0.56700	-0.82500
H	4.73400	-0.93700	-0.69100
H	3.52600	-2.05500	-0.02400
C	2.98000	0.18400	3.34300
H	1.99000	0.69200	3.37500
H	3.03600	-0.49600	4.20900
H	3.75100	0.96000	3.46300

C	0.07600	-2.98300	2.53600
H	0.02900	-3.99200	2.09200
H	0.76500	-3.03400	3.40300
H	-0.91700	-2.71000	2.93300
O	-2.46100	0.53000	-2.53200

Transition state (TS):

43

Si	0.59388	2.48946	1.93652
N	-1.86778	0.32298	0.51854
C	-1.28985	-0.52674	1.60619
C	0.25615	-0.41603	1.62874
O	0.61234	0.73063	2.19004
C	-2.45309	-0.47381	-0.60003
C	-1.89227	-1.91291	1.32884
H	-1.18904	-2.73330	1.57515
C	-2.29910	-1.93891	-0.15785
H	-2.77904	-2.07006	1.97312
H	-3.23674	-2.50271	-0.30354
H	-1.52857	-2.44900	-0.76484
H	-1.91310	-0.25532	-1.54760
H	-3.51597	-0.18105	-0.74178
H	-1.64255	-0.12522	2.60150
C	-1.73109	1.67942	0.50377
O	-1.22761	2.35877	1.44639
Cl	0.03269	2.71275	3.95986
N	1.23808	-1.21239	1.34054
Cl	0.98307	2.04877	-0.24039
Cl	0.50992	4.54566	1.48005
H	2.12282	2.47627	2.21528

H	3.04432	-0.24457	2.19979
N	3.40880	0.51278	2.81602
C	3.54817	1.76389	2.27523
H	3.85136	2.59094	2.94427
C	-2.06739	3.73767	-0.65625
H	-2.24539	4.23800	0.30433
H	-1.01723	3.86280	-0.96167
H	-2.77774	4.03196	-1.43281
O	-2.34428	2.29617	-0.54031
C	2.97508	0.38749	4.22436
H	3.15694	-0.64194	4.57489
H	3.51462	1.07998	4.88875
H	1.88200	0.59619	4.32415
C	3.98302	1.84763	0.84943
H	3.50609	1.08626	0.20073
H	3.70603	2.81264	0.38385
H	5.07250	1.73309	0.75379
C	1.09576	-2.53611	0.73544
H	2.07639	-3.03976	0.83297
H	0.33606	-3.18198	1.21367
H	0.86841	-2.46405	-0.34274

Products interaction complex (PIC):

43

Si	-0.06600	1.48900	0.51700
N	-2.11800	-1.29200	-0.92100
C	-2.02400	-1.43000	0.56600
C	-0.53800	-1.36000	0.96700
O	-0.20800	-0.05200	1.07700
C	-2.93900	-2.36800	-1.55300

C	-2.77200	-2.73400	0.88700
H	-2.07500	-3.55600	1.15900
C	-3.58000	-3.11100	-0.37000
H	-3.43400	-2.59700	1.76200
H	-4.64000	-2.81800	-0.25200
H	-3.57800	-4.20300	-0.53400
H	-2.27900	-3.02700	-2.15800
H	-3.69100	-1.92200	-2.24000
H	-2.55000	-0.55500	1.05100
C	-1.58100	-0.23800	-1.60800
O	-0.88100	0.69000	-1.16700
Cl	0.78000	2.25800	2.19500
N	0.42500	-2.17100	1.20200
Cl	1.40200	1.92800	-0.80900
N	3.51600	-0.90600	2.27400
C	3.89500	0.13500	1.26400
Cl	-1.82200	2.52600	0.54400
H	3.29600	1.07000	1.35800
H	2.65200	-1.37700	1.96200
C	-1.29000	0.80300	-3.74100
H	-1.78500	1.74000	-3.46400
H	-0.20900	0.86800	-3.56600
H	-1.53200	0.48300	-4.76100
H	4.95500	0.41100	1.45700
C	3.74500	-0.42400	-0.15100
H	4.03800	0.32100	-0.89800
H	4.36300	-1.31700	-0.29400
H	2.70300	-0.70400	-0.36000
C	3.26100	-0.29000	3.60800
H	2.44700	0.45300	3.62200
H	3.00900	-1.09100	4.32000
H	4.17800	0.19800	3.97200

C	0.36800	-3.62700	1.15800
H	-0.63300	-4.08400	1.05500
H	1.00400	-3.97600	0.31900
H	0.83600	-4.00800	2.08900
O	-1.86100	-0.29100	-2.95000

- Enantioselective reduction of the imine **1** using the organocatalyst **16**, obtaining the *R*-amine **2** as product.

Reactants interaction complex (RIC):

79

Si	-0.05000	2.11100	-1.60500
N	-2.74300	-0.37200	-1.82500
C	-2.26300	-0.52500	-0.41300
C	-0.73800	-0.37800	-0.27300
O	-0.05600	0.23000	-1.14900
C	-3.50200	-1.58000	-2.26600
C	-2.79500	-1.90100	0.04500
H	-1.98700	-2.65800	0.08000
C	-3.86100	-2.32500	-0.97400
H	-3.20800	-1.84300	1.06900
H	-4.87400	-2.05100	-0.62400
H	-3.86900	-3.41900	-1.12300
H	-2.85600	-2.19700	-2.93000
H	-4.41100	-1.28400	-2.84400
H	-2.71600	0.31500	0.20000
C	-2.54300	0.76500	-2.57800
O	-1.82800	1.66500	-2.09000
Cl	-0.96600	2.32900	0.45500
N	-0.02600	-0.92800	0.75000
Cl	0.53000	1.32000	-3.54600

N	3.00300	-0.79700	0.58200
C	3.66500	0.05900	1.29100
Cl	-0.45500	4.06100	-2.21400
H	1.30500	2.21700	-1.07600
C	2.95000	0.66600	2.45400
C	1.59300	1.81300	4.60800
C	3.28800	0.29800	3.76300
C	1.93700	1.60400	2.22000
C	1.25700	2.17400	3.30000
C	2.60700	0.87700	4.83800
H	4.07000	-0.43600	3.94200
H	1.67100	1.89400	1.19800
H	0.46200	2.89800	3.10500
H	2.86700	0.59600	5.85700
H	1.06700	2.26200	5.44900
C	3.49500	-1.45700	-0.58800
C	4.25200	-2.85500	-2.88200
C	2.87800	-1.16100	-1.81800
C	4.48500	-2.45200	-0.50100
C	4.85300	-3.14900	-1.65500
C	3.26700	-1.86100	-2.95900
H	2.10100	-0.38700	-1.88000
H	4.95000	-2.68300	0.45300
H	5.61700	-3.92300	-1.59600
H	2.80000	-1.62500	-3.91600
H	4.54900	-3.39400	-3.77800
C	5.06100	0.54800	1.06600
H	5.58300	0.00800	0.26200
H	5.67400	0.47000	1.97400
H	5.05300	1.61400	0.78000
H	1.02800	-0.79400	0.75100
C	-0.59000	-1.46000	1.96700

C	-0.33800	-2.80500	2.30700
C	-1.31200	-0.61600	2.82400
C	-0.84200	-3.31400	3.49500
H	0.25900	-3.43500	1.64400
C	-1.82800	-1.12100	4.01800
H	-1.45600	0.44000	2.56300
C	-1.58900	-2.46400	4.34200
H	-0.66700	-4.34600	3.79400
H	-2.38700	-0.46600	4.68100
O	-2.03500	-3.10600	5.47100
C	-2.76100	-2.32800	6.45500
H	-2.13200	-1.52100	6.84400
H	-3.69500	-1.95100	6.02800
H	-2.95800	-3.08000	7.23100
C	-3.20600	1.03500	-3.96300
C	-2.81600	2.45500	-4.39000
H	-1.72600	2.55000	-4.54900
H	-3.31100	2.75300	-5.31700
H	-3.04800	3.20200	-3.61600
C	-4.73000	0.94500	-3.82700
H	-5.21400	1.20300	-4.78000
H	-5.08200	-0.05900	-3.55200
H	-5.11100	1.65500	-3.08100
C	-2.64400	0.01700	-4.95800
H	-2.92200	0.28900	-5.98600
H	-1.54000	0.01100	-4.93400
H	-2.99100	-1.00400	-4.79100

Transition state (TS):

79			
Si	0.73300	2.71600	0.70300
N	-2.14900	0.71600	0.23000
C	-1.79400	0.48600	1.67500
C	-0.27500	0.33900	1.94100
O	0.49600	0.96400	1.08500
C	-3.03400	-0.37900	-0.27200
C	-2.60100	-0.76100	2.09200
H	-1.94700	-1.65000	2.20000
C	-3.63700	-1.01500	0.98700
H	-3.07600	-0.61100	3.07800
H	-4.60900	-0.55300	1.24200
H	-3.82800	-2.09300	0.85000
H	-2.42000	-1.11000	-0.84400
H	-3.82100	0.02200	-0.95300
H	-2.12500	1.39700	2.25600
C	-1.75600	1.80000	-0.49900
O	-0.98700	2.65400	0.03000
Cl	-0.13000	3.21200	2.65000
N	0.34000	-0.31200	2.89400
Cl	1.49300	2.01600	-1.21200
N	3.18300	0.61400	2.23000
C	3.38600	1.97800	2.20500
Cl	0.89800	4.73400	0.15400
H	2.14700	2.58100	1.33900
C	3.11500	2.68800	3.50100
C	2.71300	4.01700	5.93900
C	3.39300	2.03800	4.71700
C	2.63700	4.00800	3.51600
C	2.43800	4.66700	4.73400

C	3.19300	2.70200	5.92900
H	3.77000	1.01500	4.72700
H	2.40000	4.53100	2.58200
H	2.05000	5.68500	4.73400
H	3.40700	2.19300	6.86700
H	2.54900	4.53000	6.88600
C	3.66500	-0.35700	1.27800
C	4.62100	-2.38100	-0.39800
C	3.97500	-0.06600	-0.05700
C	3.81300	-1.67000	1.77500
C	4.29400	-2.67000	0.93200
C	4.45400	-1.08400	-0.88800
H	3.81200	0.93600	-0.46700
H	3.54400	-1.90400	2.80500
H	4.41300	-3.68500	1.31200
H	4.68600	-0.85700	-1.92900
H	4.99700	-3.16700	-1.05000
C	4.47500	2.57200	1.36200
H	5.45700	2.13800	1.61100
H	4.55700	3.66300	1.49800
H	4.31300	2.41400	0.27700
H	2.37000	0.26600	2.80800
C	-0.27500	-1.02500	3.95700
C	-0.07200	-2.42200	4.04100
C	-0.95300	-0.34600	4.98300
C	-0.55500	-3.12800	5.13400
H	0.47200	-2.93500	3.24700
C	-1.44100	-1.04700	6.08900
H	-1.08000	0.73700	4.92500
C	-1.24000	-2.43000	6.15100
H	-0.41400	-4.20300	5.22700
H	-1.95800	-0.51200	6.87900

O	-1.67000	-3.26300	7.16600
C	-2.33800	-2.65500	8.29600
H	-1.67100	-1.94900	8.79900
H	-3.27000	-2.18000	7.97700
H	-2.53900	-3.52700	8.93200
C	-2.24700	2.13400	-1.94300
C	-1.64800	3.48900	-2.34000
H	-0.54400	3.45500	-2.37000
H	-1.99700	3.81600	-3.32200
H	-1.88300	4.27500	-1.60700
C	-3.77600	2.23700	-1.95900
H	-4.12800	2.55000	-2.95200
H	-4.27700	1.28700	-1.72900
H	-4.13600	2.99100	-1.24600
C	-1.72100	1.04000	-2.87600
H	-1.86300	1.32500	-3.92600
H	-0.63300	0.89800	-2.74000
H	-2.20400	0.07100	-2.73800

Products interaction complex (PIC):

79

Si	-0.63800	2.89600	-0.70000
N	-2.57200	-0.15000	-1.25000
C	-2.79300	0.42100	0.11700
C	-1.42000	0.54400	0.82100
O	-0.82500	1.68000	0.45000
C	-3.21000	-1.49400	-1.39100
C	-3.81500	-0.50900	0.78700
H	-3.58500	-0.69300	1.85200
C	-3.82700	-1.81700	-0.02200

H	-4.81600	-0.03500	0.77700
H	-4.85200	-2.21400	-0.12500
H	-3.24300	-2.60100	0.50000
H	-2.44300	-2.24600	-1.69300
H	-3.98000	-1.45400	-2.19800
H	-3.22400	1.46100	0.01800
C	-1.84300	0.49700	-2.21500
O	-1.28500	1.58300	-1.93600
Cl	0.03000	4.25600	0.66800
N	-0.75700	-0.19000	1.64800
Cl	1.04000	2.85500	-1.86700
N	2.39400	-0.21900	0.12600
C	3.37200	0.89700	0.24300
Cl	-2.32900	3.92900	-1.24300
H	3.29200	1.53600	-0.68200
C	3.04000	1.73000	1.47100
C	2.47900	3.31000	3.71800
C	3.01100	1.15300	2.75000
C	2.78500	3.10000	1.32300
C	2.50500	3.88600	2.44500
C	2.73100	1.94200	3.86800
H	3.20300	0.08800	2.87200
H	2.78800	3.55700	0.33100
H	2.29400	4.94700	2.32100
H	2.70400	1.48900	4.85700
H	2.25500	3.92100	4.58900
C	2.32300	-1.06300	-0.97100
C	2.20100	-2.90600	-3.10700
C	3.08100	-0.84900	-2.15500
C	1.47000	-2.20000	-0.90200
C	1.43700	-3.10900	-1.95300
C	3.01400	-1.76800	-3.19500

H	3.70800	0.03200	-2.25000
H	0.87000	-2.37200	-0.00900
H	0.80800	-3.99500	-1.87500
H	3.60500	-1.60000	-4.09500
H	2.17600	-3.62400	-3.91700
C	4.80800	0.35900	0.35500
H	4.92100	-0.32000	1.20900
H	5.52000	1.18000	0.49100
H	5.10300	-0.20300	-0.53800
H	1.71100	-0.31400	0.88200
C	-1.20700	-1.38000	2.28400
C	-0.96400	-2.63800	1.69400
C	-1.75600	-1.29800	3.57700
C	-1.29500	-3.80200	2.37400
H	-0.48000	-2.69000	0.71000
C	-2.09600	-2.46200	4.27000
H	-1.90100	-0.32300	4.04500
C	-1.86800	-3.70200	3.65900
H	-1.11000	-4.78600	1.94600
H	-2.51700	-2.39000	5.26800
O	-2.16800	-4.93400	4.20200
C	-2.66900	-4.96800	5.56000
H	-1.93600	-4.53800	6.24900
H	-3.63600	-4.46100	5.62200
H	-2.77900	-6.04700	5.73000
C	-1.68800	-0.01900	-3.67800
C	-0.87800	1.02600	-4.45600
H	0.11400	1.19000	-4.00200
H	-0.72100	0.72100	-5.49500
H	-1.36800	2.00900	-4.45900
C	-3.08200	-0.16000	-4.29900
H	-3.00200	-0.46800	-5.35100

H	-3.70700	-0.90800	-3.79500
H	-3.62700	0.79300	-4.29100
C	-0.92700	-1.34400	-3.64300
H	-0.74900	-1.72200	-4.65800
H	0.07300	-1.22700	-3.18600
H	-1.44400	-2.13600	-3.08900

- Enantioselective reduction of the imine **1** using the organocatalyst **16**, obtaining the *S*-amine **2** as product.

Reactants interaction complex (RIC):

79

Si	-0.65900	2.40400	-0.91900
N	-2.56100	-0.53000	-2.03700
C	-2.10800	-0.93100	-0.66500
C	-0.68700	-0.44500	-0.32900
O	-0.16500	0.53000	-0.94200
C	-2.95500	-1.72600	-2.84100
C	-2.26300	-2.46700	-0.62300
H	-1.28200	-2.97700	-0.69800
C	-3.14300	-2.85600	-1.82000
H	-2.70800	-2.79300	0.33500
H	-4.20300	-2.94800	-1.51700
H	-2.85200	-3.83600	-2.23700
H	-2.14600	-1.96100	-3.56900
H	-3.89100	-1.52300	-3.41500
H	-2.79200	-0.42800	0.08700
C	-2.65500	0.78000	-2.45500
O	-2.22900	1.66400	-1.68300
Cl	-1.65600	1.83400	1.01700
N	0.11200	-1.05700	0.59100

Cl	0.18100	2.30900	-2.93600
N	2.76700	0.09000	1.53300
C	3.76600	0.44200	0.79000
Cl	-1.55100	4.27700	-1.09100
H	0.60300	2.71200	-0.25700
C	3.83000	-0.12200	-0.59100
C	3.97700	-1.12500	-3.19500
C	2.91400	0.31100	-1.55800
C	4.81400	-1.06500	-0.92000
C	4.88300	-1.56600	-2.22300
C	2.99400	-0.18900	-2.86200
H	2.13000	1.03600	-1.30500
H	5.51800	-1.40900	-0.16500
H	5.64500	-2.29700	-2.48300
H	2.28200	0.16700	-3.60900
H	4.04100	-1.51000	-4.21100
C	2.54100	0.54300	2.87100
C	1.90400	1.27400	5.49000
C	1.43400	1.37900	3.11100
C	3.32900	0.07300	3.93700
C	2.99900	0.44200	5.24400
C	1.12600	1.74000	4.42200
H	0.82200	1.74300	2.28000
H	4.17600	-0.58100	3.74600
H	3.60300	0.07800	6.07400
H	0.27100	2.39100	4.61200
H	1.65600	1.56200	6.50800
C	4.87000	1.38900	1.14400
H	4.77500	2.32000	0.56100
H	5.86000	0.97300	0.91200
H	4.86900	1.67700	2.20500
H	1.07200	-0.64300	0.77000

C	-0.34000	-2.01500	1.57300
C	0.23500	-3.30200	1.58800
C	-1.28200	-1.62700	2.53800
C	-0.16300	-4.21500	2.55400
H	0.99400	-3.57100	0.85100
C	-1.69100	-2.54100	3.51100
H	-1.68300	-0.60700	2.53700
C	-1.12800	-3.82500	3.51100
H	-2.41800	-2.23800	4.26000
H	0.26000	-5.21800	2.60200
O	-1.43200	-4.83200	4.39300
C	-2.35100	-4.53400	5.47400
H	-1.95000	-3.73700	6.10600
H	-3.33800	-4.28600	5.07600
H	-2.37300	-5.49000	6.01500
C	-3.32300	1.23600	-3.78700
C	-2.48000	0.69600	-4.94500
H	-2.54500	-0.38500	-5.07400
H	-2.79100	1.15400	-5.89400
H	-1.41600	0.96100	-4.81500
C	-4.77100	0.73400	-3.83100
H	-5.35000	1.09900	-2.97200
H	-5.27700	1.10800	-4.73200
H	-4.84900	-0.36200	-3.85200
C	-3.31400	2.76800	-3.80500
H	-3.76200	3.19900	-2.89600
H	-2.28500	3.17200	-3.82400
H	-3.84200	3.17000	-4.67200

Transition state (TS):

79

Si	0.35100	2.56800	1.59900
N	-2.43200	0.42400	1.10500
C	-1.90600	0.00800	2.45300
C	-0.36200	-0.06600	2.53700
O	0.27200	0.75600	1.73600
C	-3.30900	-0.64200	0.53200
C	-2.59500	-1.34100	2.74900
H	-1.88800	-2.18900	2.64700
C	-3.73500	-1.49600	1.73300
H	-2.96200	-1.37500	3.79100
H	-4.69300	-1.14300	2.15500
H	-3.88700	-2.55200	1.45100
H	-2.72500	-1.23700	-0.20600
H	-4.18700	-0.20000	0.00400
H	-2.22100	0.79600	3.20000
C	-2.19400	1.63600	0.52400
O	-1.42900	2.45600	1.10800
Cl	-0.33000	2.67500	3.68600
N	0.39700	-0.82500	3.28300
Cl	0.94000	2.19200	-0.44800
N	2.67400	1.16100	3.93100
C	3.13300	2.00500	2.94200
Cl	0.30300	4.64800	1.37600
H	1.82300	2.46400	2.08500
C	3.86100	1.29300	1.83700
C	5.29600	-0.03700	-0.17300
C	3.22600	0.33800	1.03100
C	5.22500	1.57500	1.63400
C	5.93700	0.91100	0.63500

C	3.94500	-0.32200	0.02800
H	2.15400	0.11100	1.16300
H	5.73800	2.30800	2.25600
H	6.99100	1.13400	0.48100
H	3.43600	-1.05000	-0.60300
H	5.85300	-0.54600	-0.95900
C	2.20200	1.49300	5.25300
C	1.38800	1.91300	7.90000
C	1.86300	2.78400	5.68200
C	2.10900	0.40200	6.14600
C	1.70700	0.62200	7.46200
C	1.45800	2.98400	7.00600
H	1.86600	3.63300	4.99100
H	2.33200	-0.61100	5.80800
H	1.63900	-0.21900	8.15100
H	1.18500	3.98800	7.33300
H	1.07600	2.07900	8.92800
C	3.57300	3.40200	3.26100
H	2.75700	4.01700	3.69600
H	3.89000	3.94800	2.35500
H	4.40600	3.41700	3.98000
H	2.40200	0.19400	3.62800
C	-0.05000	-1.76200	4.25300
C	0.26000	-3.12900	4.06500
C	-0.66000	-1.33300	5.44500
C	-0.05300	-4.05300	5.05200
H	0.75300	-3.44600	3.14700
C	-0.97800	-2.25500	6.44600
H	-0.86600	-0.27200	5.59800
C	-0.67500	-3.60500	6.23700
H	-1.44500	-1.91200	7.36300
H	0.17400	-5.11100	4.93600

O	-0.93400	-4.63600	7.11900
C	-1.54300	-4.29800	8.38700
H	-0.89100	-3.63200	8.95900
H	-2.53400	-3.86300	8.23000
H	-1.61800	-5.28200	8.86900
C	-2.85700	2.14500	-0.79400
C	-2.37400	1.24400	-1.93400
H	-2.77900	0.23200	-1.90100
H	-2.64900	1.67100	-2.90800
H	-1.27100	1.16900	-1.93400
C	-4.38200	2.13700	-0.64300
H	-4.70500	2.74800	0.21100
H	-4.85700	2.56600	-1.53500
H	-4.80000	1.12800	-0.51400
C	-2.38200	3.58400	-1.03200
H	-2.57600	4.23200	-0.16500
H	-1.28900	3.63400	-1.18200
H	-2.85800	4.03000	-1.90900

Products interaction complex (PIC):

79

Si	-0.03900	2.21200	-2.44100
N	-2.54200	0.12300	-0.75900
C	-1.30000	0.30700	0.06100
C	-0.09700	-0.14300	-0.79200
O	0.41700	0.91300	-1.45400
C	-3.51100	-0.78900	-0.07800
C	-1.52200	-0.48300	1.35600
H	-0.64000	-1.09700	1.62400
C	-2.77800	-1.34500	1.15300

H	-1.64900	0.22000	2.20600
H	-3.42400	-1.32400	2.04800
H	-2.49700	-2.40600	0.99700
H	-3.82800	-1.59900	-0.77600
H	-4.42100	-0.21100	0.20800
H	-1.17500	1.40700	0.30000
C	-2.70100	0.70800	-1.98400
O	-1.76800	1.39400	-2.46700
Cl	1.83900	3.03400	-2.30500
N	0.49000	-1.26800	-1.00200
Cl	-0.09600	1.93700	-4.45100
N	2.02800	0.63500	1.96900
C	2.96400	0.95900	0.84700
Cl	-1.01900	3.77600	-1.52900
H	2.32800	1.22300	-0.05200
C	3.77600	-0.29100	0.54600
C	5.26200	-2.59000	-0.06400
C	3.62300	-0.92000	-0.69800
C	4.68000	-0.81600	1.48100
C	5.41900	-1.96300	1.17600
C	4.36400	-2.06600	-0.99900
H	2.91100	-0.52400	-1.43200
H	4.82100	-0.32700	2.44200
H	6.12100	-2.36500	1.90400
H	4.23500	-2.55100	-1.96600
H	5.83800	-3.48200	-0.30100
C	1.06600	1.58300	2.39000
C	-1.03300	3.26200	3.21700
C	0.61300	2.62300	1.55000
C	0.46000	1.40000	3.65900
C	-0.57900	2.23900	4.05500
C	-0.42700	3.45000	1.97000

H	1.07100	2.78900	0.57200
H	0.80600	0.61600	4.32400
H	-1.03800	2.09800	5.03300
H	-0.76800	4.25200	1.31200
H	-1.83900	3.91500	3.53600
C	3.88900	2.14100	1.16400
H	3.31900	3.06200	1.33700
H	4.56400	2.33600	0.32000
H	4.51100	1.96700	2.04600
H	2.47900	0.12200	2.73000
C	0.18200	-2.50900	-0.37900
C	-0.87900	-3.30600	-0.85800
C	1.04300	-2.99200	0.62500
C	-1.09000	-4.57100	-0.32100
H	-1.51400	-2.93700	-1.66000
C	0.83500	-4.25800	1.17200
H	1.88100	-2.37400	0.96100
C	-0.23100	-5.03400	0.69500
H	1.50400	-4.62600	1.94400
H	-1.89100	-5.21500	-0.67700
O	-0.57400	-6.29300	1.14700
C	0.28900	-6.91400	2.12800
H	1.30000	-7.03300	1.72600
H	0.28500	-6.33800	3.05700
H	-0.19500	-7.88900	2.26700
C	-3.98900	0.57700	-2.85100
C	-4.16400	-0.89600	-3.23400
H	-4.30200	-1.55500	-2.36700
H	-5.04300	-1.02600	-3.87900
H	-3.30100	-1.27000	-3.80400
C	-5.17900	1.11300	-2.05000
H	-5.01600	2.15200	-1.73100

H	-6.08900	1.11500	-2.66600
H	-5.40300	0.52300	-1.15400
C	-3.79000	1.42500	-4.11400
H	-3.64200	2.48800	-3.87300
H	-2.88800	1.12400	-4.67100
H	-4.64500	1.34800	-4.79100

Mechanistic studies with real compounds

Results for the former calculations, where only the energy barrier between RIC and TS ($\Delta E_{RIC\text{-}TS}$) was taking account:

***UP*-conformation**	Uncatalyzed reaction		Catalyst **3**		Catalyst **5**		Catalyst **6**		Catalyst **12**		Catalyst **13**		Catalyst **14**		Catalyst **16**	
	R	***S***	***R***	***S***	***R***	***S***	***R***	***S***	***R***	***S***	***R***	***S***	***R***	***S***	***R***	***S***
**E (RIC) (Eh)**	-0.097484	-0.097484	-0.239568	-0.241484	n.c	n.c	-0.314707	-0.313685	-0.279601	-0.280049	-0.304736	-0.306128	-0.244725	-0.245157	-0.310621	-0.312443
**E (TS) (Eh)**	-0.050661	-0.050661	-0.211205	-0.213466	n.c	n.c	-0.278295	-0.280899	-0.249050	-0.251373	-0.273985	-0.276328	-0.223486	-0.225336	-0.280279	-0.282300
**E (PIC) (Eh)**	-0.138578	-0.138578	-0.261901	-0.266794	n.c	n.c	-0.330338	-0.335696	-	-	-0.324978	-0.330173	-0.271745	-0.266589	-	-
**E (RIC) - E (TS) (Eh)**	0.046823	0.046823	0.028363	0.028018	-	-	0.036413	0.032786	0.030551	0.028675	0.030751	0.029800	0.021239	0.019821	0.030341	0.030143
**E (RIC) - E (TS) (kcal)**	29.382036	29.382036	17.798239	17.581271	-	-	22.849404	20.573610	19.171304	17.993996	19.296623	18.699693	13.327463	12.437755	19.039533	18.914954
**E (Enant. *S*) - E(Enant. *R*) (kcal)**		0.000000		-0.216968		-		-2.275793		-1.177307		-0.596930		-0.889707		-0.124579
**S/R=Exp(-(E enant S - E enant R)/(RT))**		1.000000		1.491729		-		66.351551		8.759416		3.005132		5.155152		1.258143
***ee* (%)**		0		20		-		97		80		50		68		11

***DOWN*-conformation**	Uncatalyzed reaction		Catalyst **3**		Catalyst **5**		Catalyst **6**		Catalyst **12**		Catalyst **13**		Catalyst **14**		Catalyst **16**	
	R	***S***	***R***	***S***	***R***	***S***	***R***	***S***	***R***	***S***	***R***	***S***	***R***	***S***	***R***	***S***
**E (RIC) (Eh)**	-0.097484	-0.097484	-0.240563	-0.241412	-0.291363	n.c	-0.314765	-0.314527	-0.279668	-0.280770	-0.305645	-0.305294	-0.246208	-0.245187	-0.309844	-0.309401
**E (TS) (Eh)**	-0.050661	-0.050661	-0.212279	-0.213950	-0.213477	n.c	-0.278620	-0.279165	-0.250119	-0.251812	-0.275304	-0.276931	-0.224423	-0.226119	-0.279021	-0.280549
**E (PIC) (Eh)**	-0.138578	-0.138578	-0.261901	-0.266899	-0.298274	n.c	-0.330338	-0.335628	-0.300139	-0.300139	-0.324979	-0.330173	-0.269371	-0.278239	-0.330763	-0.338027
**E (RIC) - E (TS) (Eh)**	0.046823	0.046823	0.028284	0.027462	-	-	0.036145	0.035361	0.029549	0.028958	0.030341	0.028363	0.021785	0.019068	0.030824	0.028852
**E (RIC) - E (TS) (kcal)**	29.382036	29.382036	17.748722	17.232828	-	-	22.681325	22.189440	18.542257	18.171199	19.039439	17.798057	13.670422	11.965204	19.342143	18.104965
**E (Enant. *S*) - E(Enant. *R*) (kcal)**		0.000000		-0.515893974		-		-0.49188548		-0.371058622		-1.241382229		-1.705218057		-1.237177919
**S/R=Exp(-(E enant S - E enant R)/(RT))**		1.000000		2.588159		-		2.476118		1.981733		9.857559		23.178535		9.781460
***ee* (%)**		0		44		-		42		33		82		92		81

	Uncatalyzed reaction	Catalyst **3**	Catalyst **5**	Catalyst **6**	Catalyst **12**	Catalyst **13**	Catalyst **14**	Catalyst **16**
***ee* average (%)**	0	32	-	70	56	66	80	46

Results for the second calculations, where the energy barrier ΔE_{TOT} was taking account:

E (imine **1**) (Eh) = 0.097076

E ($HSiCl_3$) (Eh) = -0.179294

	Uncatalyzed reaction	Catalyst **3**	Catalyst **6**	Catalyst **12**	Catalyst **13**	Catalyst **14**	Catalyst **16**
**E catalyst (Conf 1) (Eh)**	-	-0.139256	-0.210245	-0.177990	-0.201985	-0.151496	-0.205350
**E catalyst (Conf 2) (Eh)**	-	-0.139819	-0.211703	-0.177644	-0.202790	-0.152195	Not exist

***UP*-conformation**	Uncatalyzed reaction		Catalyst **3**		Catalyst **6**		Catalyst **12**		Catalyst **13**		Catalyst **14**		Catalyst **16**	
	R	***S***	***R***	***S***	***R***	***S***	***R***	***S***	***R***	***S***	***R***	***S***	***R***	***S***
**E ABC (Conf 1) (Eh)**	-0.082218	-0.082218	-0.221474	-0.221474	-0.292463	-0.292463	-0.260208	-0.260208	-0.284203	-0.284203	-0.233714	-0.233714	-0.287569	-0.287569
**E ABC (conf 2) (Eh)**	-0.082218	-0.082218	-0.222037	-0.222037	-0.293921	-0.293921	-0.259862	-0.259862	-0.285008	-0.285008	-0.234413	-0.234413	-	-
**E (RIC) (Eh)**	-0.097484	-0.097484	-0.239568	-0.241484	-0.314707	-0.313685	-0.279601	-0.280049	-0.304736	-0.306128	-0.244725	-0.245157	-0.310621	-0.312443
**E (TS) (Eh)**	-0.050661	-0.050661	-0.211205	-0.213466	-0.278295	-0.280899	-0.249050	-0.251373	-0.273985	-0.276328	-0.223486	-0.225336	-0.280279	-0.282300
**E (PIC) (Eh)**	-0.138578	-0.138578	-0.261901	-0.266794	-0.330338	-0.335696	-	-	-0.324978	-0.330173	-0.271745	-0.266589	-	-
**E (ABC) - E (RIC) (conf 1) (Eh)**	-0.015266	-0.015266	-0.018094	-0.020010	-0.022244	-0.021222	-0.019393	-0.019841	-0.020533	-0.021925	-0.011011	-0.011443	-0.023052	-0.024875
**E (ABC) - E (RIC) (conf 2) (Eh)**	-0.015266	-0.015266	-0.017531	-0.019447	-0.020786	-0.019763	-0.019739	-0.020186	-0.019728	-0.021120	-0.010312	-0.010744	-	-
**E (ABC) - E (RIC) (conf 1) (kcal)**	-9.579515	-9.579515	-11.354248	-12.556455	-13.958574	-13.316833	-12.169307	-12.450130	-12.884881	-13.757978	-6.909213	-7.180730	-14.465281	-15.609086
**E (ABC) - E (RIC) (conf 2) (kcal)**	-9.579515	-9.579515	-11.000816	-12.203023	-13.043433	-12.401693	-12.386155	-12.666978	-12.379717	-13.252814	-6.470678	-6.742195	-	-
**E (RIC) - E (TS) (Eh)**	0.046823	0.046823	0.028363	0.028018	0.036413	0.032786	0.030551	0.028675	0.030751	0.029800	0.021239	0.019821	0.030341	0.030143
**E (RIC) - E (TS) (kcal)**	29.382036	29.382036	17.798239	17.581271	22.849404	20.573610	19.171304	17.993996	19.296623	18.699693	13.327463	12.437755	19.039533	18.914954
**E TOT (conf 1) (kcal)**	19.802521	19.802521	6.443990	5.024816	8.890830	7.256778	7.001996	5.543866	6.411743	4.941715	6.418250	5.257026	4.574252	3.305868
**E TOT (conf 2) (kcal)**	19.802521	19.802521	6.797422	5.378248	9.805970	8.171918	6.785148	5.327018	6.916906	5.446879	6.856785	5.695560	-	-
**E TOT average (kcal)**	19.802521	19.802521	6.620706	5.201532	9.348400	7.714348	6.893572	5.435442	6.664324	5.194297	6.637517	5.476293	4.574252	3.305868
**E (Enant. *S*) - E(Enant. *R*) (kcal)**		0.000000		-1.419174		-1.634052		-1.458130		-1.470028		-1.161224		-1.268384
**S/R=Exp(-(E enant S - E enant R)/(RT))**		1.000000		13.680305		20.328841		14.698775		15.024691		8.503510		10.360557
***ee* (%)**		0		86		91		87		88		79		82

***DOWN*-conformation**	Uncatalyzed reaction		Catalyst **3**		Catalyst **6**		Catalyst **12**		Catalyst **13**		Catalyst **14**		Catalyst **16**	
	R	***S***	***R***	***S***	***R***	***S***	***R***	***S***	***R***	***S***	***R***	***S***	***R***	***S***
**E ABC (Conf 1) (Eh)**	-0.082218	-0.082218	-0.221474	-0.221474	-0.292463	-0.292463	-0.260208	-0.260208	-0.284203	-0.284203	-0.233714	-0.233714	-0.287569	-0.287569
**E ABC (conf 2) (Eh)**	-0.082218	-0.082218	-0.222037	-0.222037	-0.293921	-0.293921	-0.259862	-0.259862	-0.285008	-0.285008	-0.234413	-0.234413	-	-
**E (RIC) (Eh)**	-0.097484	-0.097484	-0.240563	-0.241412	-0.314765	-0.314527	-0.279668	-0.280770	-0.305645	-0.305294	-0.246208	-0.245187	-0.309844	-0.309401
**E (TS) (Eh)**	-0.050661	-0.050661	-0.212279	-0.213950	-0.278620	-0.279165	-0.250119	-0.251812	-0.275304	-0.276931	-0.224423	-0.226119	-0.279021	-0.280549
**E (PIC) (Eh)**	-0.138578	-0.138578	-0.261901	-0.266899	-0.330338	-0.335628	-0.300139	-0.300139	-0.324979	-0.330173	-0.269371	-0.278239	-0.330763	-0.338027
**E (ABC) - E (RIC) (conf 1) (Eh)**	-0.015266	-0.015266	-0.019089	-0.019938	-0.022302	-0.022063	-0.019459	-0.020562	-0.021442	-0.021091	-0.012494	-0.011473	-0.022276	-0.021832
**E (ABC) - E (RIC) (conf 2) (Eh)**	-0.015266	-0.015266	-0.018526	-0.019375	-0.020843	-0.020605	-0.019805	-0.020907	-0.020637	-0.020286	-0.011795	-0.010774	-	-
**E (ABC) - E (RIC) (conf 1) (kcal)**	-9.579515	-9.579515	-11.978714	-12.511463	-13.994411	-13.845001	-12.210993	-12.902765	-13.455274	-13.234679	-7.840010	-7.199166	-13.978127	-13.700071
**E (ABC) - E (RIC) (conf 2) (kcal)**	-9.579515	-9.579515	-11.625282	-12.158031	-13.079270	-12.929861	-12.427841	-13.119613	-12.950110	-12.729516	-7.401475	-6.760631	-	-
**E (RIC) - E (TS) (Eh)**	0.046823	0.046823	0.028284	0.027462	0.036145	0.035361	0.029549	0.028958	0.030341	0.028363	0.021785	0.019068	0.030824	0.028852
**E (RIC) - E (TS) (kcal)**	29.382036	29.382036	17.748722	17.232828	22.681325	22.189440	18.542257	18.171199	19.039439	17.798057	13.670422	11.965204	19.342143	18.104965
**E TOT (conf 1) (kcal)**	19.802521	19.802521	5.770008	4.721365	8.686915	8.344439	6.331265	5.268434	5.584165	4.563377	5.830412	4.766038	5.364016	4.404894
**E TOT (conf 2) (kcal)**	19.802521	19.802521	6.123440	5.074797	9.602055	9.259579	6.114416	5.051586	6.089329	5.068541	6.268947	5.204572	-	-
**E TOT average (kcal)**	19.802521	19.802521	5.946724	4.898081	9.144485	8.802009	6.222840	5.160010	5.836747	4.815959	6.049679	4.985305	5.364016	4.404894
**E (Enant. *S*) - E(Enant. *R*) (kcal)**		0.000000		-1.048643		-0.342476		-1.062831		-1.020788		-1.064374		-0.959122
**S/R=Exp(-(E enant S - E enant R)/(RT))**		1.000000		6.909921		1.880022		7.093018		6.564084		7.113229		5.858805
***ee* (%)**		0		75		31		75		74		75		71

	Uncatalyzed reaction	Catalyst **3**	Catalyst **6**	Catalyst **12**	Catalyst **13**	Catalyst **14**	Catalyst **16**
***ee* average (%)**	0	81	61	81	81	77	77

3. Synthetic procedures and characterization

Synthesis of imine 1

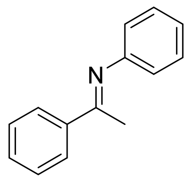

1

The product was synthesized according to the general procedure 3.2.

Yield: 70%.

Spectral data is in agreement with the reported literature.

^{1}H-NMR (500 MHz, $CDCl_3$): δ 2.25 (s, 3H), 6.78-6.84 (m, 2H), 7.06-7.12 (m, 1H), 7.33-7.38 (m, 2H), 7.42-7.50 (m, 3H), 7.96-8.01 (m, 2H).
^{13}C-NMR (125 MHz, $CDCl_3$): δ 17.5, 119.5, 123.3, 127.3, 128.5, 129.1, 130.6, 139.6, 151.8, 165.5.
Melting point: 41–42 °C.

Synthesis of pivaloyl-L-proline

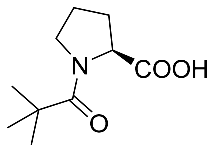

Piv-*L*-proline

The product was synthesized according to the general procedure 3.3.

Yield: 65%.

Spectral data is in agreement with the reported literature.

^{1}H-NMR (500 MHz, $CDCl_3$): δ 1.26 (s, 9H), 1.89-2.00 (m, 1H), 2.01-2.09 (m, 2H), 2.09-2.15 (m, 1H), 3.65-3.78 (m, 2H), 4.50-4.60 (m, 1H), 11.36 (bs, 1H).
^{13}C-NMR (125 MHz, $CDCl_3$): δ 26.0, 27.2, 27.3, 39.1, 48.5, 61.6, 175.7, 178.6.
Melting point: 128–130 °C.

Synthesis of catalyst 3

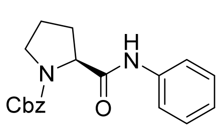

3

According to the general procedure 3.4., Cbz-L-proline (1 g, 4.012 mmol), triethylamine (0.562 mL, 4.012 mmol), ethyl chloroformate (0.395 mL, 4.012 mmol) and aniline (0.746 mL, 4.012 mmol) afforded **3** as a white solid (yield 62%). Purification by crystallization in CH_2Cl_2/*n*-hexane.

^{1}H-NMR (500 MHz, $CDCl_3$): δ 1.81-2.69 (m, 4H), 3.37-3.73 (m, 2H), 4.51 (s, 1H), 4.99-5.35 (m, 2H), 6.99-7.59 (m, 10H), 7.66 y 9.15 (s, 1H).
^{13}C-NMR (125 MHz, $CDCl_3$): δ 24.7, 27.7, 47.2, 61.2, 67.7, 119.9, 124.1, 128.0, 128.3, 128.7, 128.9, 136.4, 138.3, 156.8, 169.5.

IR (ATR): ν_{max} (cm^{-1}) 1425, 1441, 1466, 1494, 1550, 1602, 1665, 1698, 2882, 3088, 3136, 3199, 3240, 3276, 3310.
ESI-MS (CH_3OH): m/z calcd for $C_{19}H_{20}N_2O_3$ 325.1552 [M+H$^+$], found 325.1549.
Melting point: 134-138 °C.
[α]^{25}$_D$ (CH_2Cl_2): -118.66.

Synthesis of catalyst 4

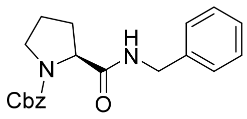

4

According to the general procedure 3.4., Cbz-L-proline (1 g, 4.012 mmol), triethylamine (0.562 mL, 4.012 mmol), ethyl chloroformate (0.395 mL, 4.012 mmol) and benzylamine (0.443 mL, 4.012 mmol) afforded **4** as a white solid (yield 62%). Purification by crystallization in IPA.

^{1}H-NMR (500 MHz, $CDCl_3$): δ 1.94 (d, J = 30.7 Hz, 2H), 2.21 (t, J = 26.2 Hz, 1H), 2.30-2.50 (m, 1H), 3.49 (d, J = 27.8 Hz, 2H), 4.39 (s, 3H), 5.13 (s, 2H), 6.24 and 7.04 (s, 1H), 7.08-7.49 (m, 10H).
^{13}C-NMR (125 MHz, $CDCl_3$): δ 24.8, 28.6, 43.6, 47.3, 60.9, 67.5, 127.5, 127.6, 128.1, 128.3, 128.7, 128.8, 136.5, 138.3, 156.4, 171.7.
IR (ATR): ν_{max} (cm^{-1}) 1415, 1440, 1452, 1497, 1534, 1651, 1693, 1714, 2871, 2907, 2958, 2993, 3030, 3287.
ESI-MS (CH_3OH): m/z calcd for $C_{20}H_{22}N_2O_3$ 339.17 [M+H$^+$], found 339.3.
Melting point: 90-95 °C.
[α]^{25}$_D$ (CH_2Cl_2): -82.48.

Synthesis of catalyst 5

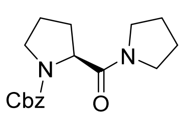

5

According to the general procedure 3.4., Cbz-L-proline (5 g, 20.06 mmol), triethylamine (2.809 g, 20.06 mmol), ethyl chloroformate (1.977 mL, 20.06 mmol) and pyrrolidine (1.659 mL, 20.06 mmol) afforded **5** as a white solid (yield 36%). Purification by crystallization in CH_2Cl_2/H_2O.

^{1}H-NMR (500 MHz, $CDCl_3$): δ 1.54-1.68 (m, 1H), 1.69-2.05 (m, 5H), 2.07-2.21 (m, 2H), 3.22-3.45 (m, 3H), 3.45-3.81 (m, 3H), 4.36-4.57 (m, 1H), 4.94-5.23 (m, 2H), 7.23-7.40 (m, 5H).
^{13}C-NMR (125 MHz, $CDCl_3$): δ 23.9, 26.4, 29.7, 46.1, 46.1, 58.3, 66.9, 127.9, 128.2, 128.5, 137.0, 155.0, 170.9.
IR (ATR): ν_{max} (cm^{-1}) 1415, 1439, 1469, 1639, 1700, 2877, 2952, 2973.
ESI-MS (CH_3OH): m/z calcd for $C_{17}H_{22}N_2O_3$ 303.1709 [M+H$^+$], found 303.1709.
Melting point: 133-134 °C.
[α]^{25}$_D$ (CH_2Cl_2): -2.18.

Synthesis of catalyst 6

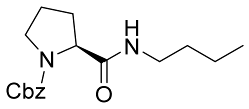

6

According to the general procedure 3.4., Cbz-L-proline (4 g, 16.047 mmol), triethylamine (2.247 mL, 16.047 mmol), ethyl chloroformate (1.582 mL, 16.047 mmol) and butylamine (1.586 mL, 16.047 mmol) afforded **6** as a white solid (yield 80%). Purification by crystallization in CH_2Cl_2/*n*-hexane.

^{1}H-NMR (500 MHz, $CDCl_3$): δ 0.77-0.95 (m, 3H), 1.12-1.36 (m, 3H), 1.36-1.50 (m, 1H), 1.78-2.02 (m, 2H), 2.04-2.23 (m, 1H), 2.25-2.43 (s, 1H), 3.07-3.28 (m, 2H), 3.33-3.60 (m, 2H), 4.29 (s, 1H), 4.99-5.24 (m, 2H), 5.94 y 6.70 (s, 1H), 7.27-7.45 (m, 5H).
^{13}C-NMR (125 MHz, $CDCl_3$): δ 13.75, 20.05, 24.62, 28.30, 31.60, 39.23, 47.11, 60.71, 67.35, 128.03, 128.19, 128.57, 136.49, 156.28, 171.57.
IR (ATR): ν_{max} (cm^{-1}) 1418, 1459, 1550, 1648, 1693, 1713, 2873, 2934, 2962, 3303.
ESI-MS (CH_3OH): m/z calcd for $C_{17}H_{24}N_2O_3$ 305.18 [M+H$^+$], found 305.2.
Melting point: 85-86 °C.
$[\alpha]^{25}_D$ (CH_2Cl_2): -78.50.

Synthesis of catalyst 7

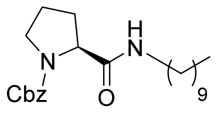

7

According to the general procedure 3.4., Cbz-L-proline (6 g, 24.071 mmol), triethylamine (3.370 mL, 24.071 mmol), ethyl chloroformate (2.373 mL, 24.071 mmol) and decylamine (5.064 mL, 24.071 mmol) afforded **7** as a white solid (yield 54%). Purification by crystallization in CH_2Cl_2/*n*-hexane.

^{1}H-NMR (500 MHz, $CDCl_3$): δ 0.88 (t, J = 6.8 Hz, 3H), 1.12-1.53 (m, 16H), 1.79-2.04 (m, 2H), 2.05-2.26 (m, 1H), 2.27-2.48 (m, 1H), 3.07-3.30 (m, 2H), 3.37-3.62 (m, 2H), 4.32 (d, J = 5.6 Hz, 1H), 5.04-5.24 (m, 2H), 5.88 y 6.68 (s, 1H), 7.27-7.42 (m, 5H).
^{13}C-NMR (125 MHz, $CDCl_3$): δ 14.2, 22.8, 23.9, 24.7, 26.9, 29.4, 29.5, 29.6, 29.7 (2C), 32.0, 39.6, 47.2, 60.8, 67.5, 128.1, 128.3, 128.7, 136.5, 156.4, 171.5.
IR (ATR): ν_{max} (cm^{-1}) 1417, 1459, 1558, 1651, 1693, 1712, 2851, 2918, 2955, 3303.
ESI-MS (CH_3OH): m/z calcd for $C_{23}H_{36}N_2O_3$ 389.28 [M+H$^+$], found 389.4.
Melting point: 87-90 °C.
$[\alpha]^{25}_D$ (CH_2Cl_2): -60.76.

Synthesis of catalyst 8

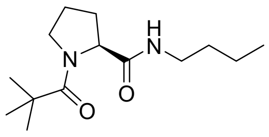

8

According to the general procedure 3.4., Piv-L-proline (3 g, 15.056 mmol), triethylamine (2.108 mL, 15.056 mmol), ethyl chloroformate (1.484 mL, 15.056 mmol) and butylamine (1.496 mL, 15.056 mmol) afforded **8** as a white solid (yield 50%). Purification by crystallization in *n*-hexane.

^{1}H-NMR (500 MHz, $CDCl_3$): δ 0.88 (t, J = 7.3 Hz, 3H), 1.25 (s, 9H), 1.27-1.34 (m, 2H), 1.37-1.47 (m, 2H), 1.76-1.85 (m, 1H), 1.86-1.95 (m, 1H), 2.02-2.16 (m, 1H), 2.24-2.29 (m, 1H), 3.12-3.26 (m, 2H), 3.62 (ddd, J = 10.2, 7.8, 4.8 Hz, 1H), 3.70 (dt, J = 10.0, 7.4 Hz, 1H), 4.60 (dd, J = 8.1, 3.1 Hz, 1H), 6.72 (s, 1H).
^{13}C-NMR (125 MHz, $CDCl_3$): δ 13.8, 20.1, 25.9, 26.6, 27.7, 31.7, 39.2, 39.4, 48.4, 61.7, 171.9, 177.9.
IR (ATR): ν_{max} (cm^{-1}) 1418, 1478, 1552, 1601, 1686, 2859, 2874, 2934, 2958, 3307.
ESI-MS (CH_3OH): m/z calcd for $C_{14}H_{26}N_2O_2$ 255.20 [M+H$^+$], found 255.3.
Melting point: 57-60 °C.
[α]$^{25}_D$ (CH_2Cl_2): -105.68.

Synthesis of catalyst 9

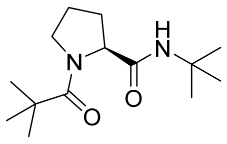

9

According to the general procedure 3.4., Piv-L-proline (1 g, 5.019 mmol), triethylamine (0.703 mL, 5.019 mmol), ethyl chloroformate (0.495 mL, 5.019 mmol) and *tert*-butylamine (0.538 mL, 5.019 mmol) afforded **9** as a white solid (yield 30%). No purification was needed.

^{1}H-NMR (500 MHz, $CDCl_3$): δ 1.21 (s, 9H), 1.25 (s, 9H), 1.72-1.79 (m, 1H), 1.80-1.88 (m, 1H), 1.99-2.07 (m, 1H), 2.15-2.21 (m, 1H), 3.54-3.61 (m, 1H), 3.66 (dt, J = 10.1, 7.3 Hz, 1H), 4.49 (dd, J = 8.2, 3.1 Hz, 1H), 6.49 (s, 1H).
^{13}C-NMR (125 MHz, $CDCl_3$): δ 25.7, 26.6, 27.6, 28.7, 39.3, 48.3, 50.9, 62.2, 171.1, 177.7.
IR (ATR): ν_{max} (cm^{-1}) 1417, 1454, 1478, 1509, 1552, 1585, 1684, 2879, 2933, 2941, 2960, 2988, 3069, 3235, 3291.
ESI-MS (CH_3OH): m/z calcd for $C_{14}H_{26}N_2O_2$ 255.20 [M+H$^+$], found 255.30.
Melting point: 96-98 °C.
[α]$^{25}_D$ (CH_2Cl_2): -117.44.

Synthesis of catalyst 10

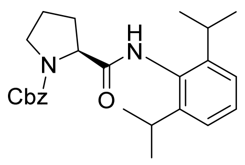

10

According to the general procedure 3.4., Cbz-L-proline (1 g, 4.012 mmol), triethylamine (0.562 mL, 4.012 mmol), ethyl chloroformate (0.395 mL, 4.012 mmol) and 2,6-diisopropylaniline (0.780 mL, 4.012 mmol) afforded **10** as a beige solid (yield 52%). Purification by crystallization in CH_2Cl_2/*n*-hexane.

^{1}H-NMR (500 MHz, $CDCl_3$): δ 1.12 (s, 12H), 1.99 (m, 3H), 2.38 (m, 1H), 2.97 (s, 2H), 3.55 (s, 2H), 4.61 (d, J = 5.2 Hz, 1H), 4.88-5.50 (m, 2H), 7.12 (d, J = 7.7 Hz, 2H), 7.22-7.27 (m, 1H), 7.28-7.39 (m, 5H), 8.10 (s, 1H).
^{13}C-NMR (125 MHz, $CDCl_3$): δ 23.6, 24.9, 28.1, 28.9, 47.3, 60.9, 67.8, 123.5, 128.0, 128.4, 128.8, 131.4, 136.4, 146.3, 156.8, 170.9.
IR (ATR): ν_{max} (cm^{-1}) 1412, 1445, 1523, 1658, 1677, 1704, 2869, 2960, 3239.
ESI-MS (CH_3OH): m/z calcd for $C_{25}H_{32}N_2O_3$ 409.2491 [M+H$^+$], found 409.2499.
Melting point: 110-112 °C.
[α]$^{25}_D$ (CH_2Cl_2): -107.66.

Synthesis of catalyst 11

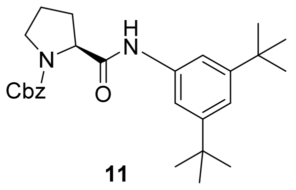

11

According to the general procedure 3.4., Cbz-L-proline (0.5 g, 2.006 mmol), triethylamine (0.280 mL, 2.006 mmol), ethyl chloroformate (0.198 mL, 2.006 mmol) and 3,5-di-*tert*-butylaniline (0.412 g, 2.006 mmol) afforded **11** as a white solid (yield 33%). Purification by crystallization in CH_2Cl_2/*n*-hexane.

^{1}H-NMR (500 MHz, $CDCl_3$): δ 1.32 (s, 18H), 1.86-1.99 (m, 1H), 2.00-2.15 (m, 1H), 2.16-2.39 (m, 1H), 2.45-2.62 (m, 1H), 3.38-3.75 (m, 2H), 4.52 (s, 1H), 5.21 (s, 2H), 7.13-7.46 (m, 8H), 7.68 and 9.05 (s, 1H).
^{13}C-NMR (125 MHz, $CDCl_3$): δ 24.8, 27.6, 31.5, 35.0, 47.2, 61.3, 67.7, 114.5, 118.4, 127.9, 128.3, 128.7, 136.4, 151.67, 156.9, 169.3.
IR (ATR): ν_{max} (cm^{-1}) 1412, 1446, 1562, 1604, 1614, 1664, 1711, 2868, 2902, 2958, 3267.
ESI-MS (CH_3OH): m/z calcd for $C_{27}H_{36}N_2O_3$ 437.2804 [M+H$^+$], found 437.2797.
Melting point: 165-167 °C.
[α]$^{25}_D$ (CH_2Cl_2): -86.60.

Synthesis of catalyst 12

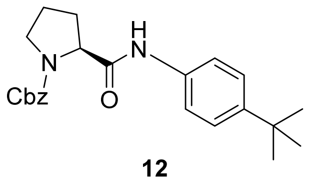

12

According to the general procedure 3.4., Cbz-L-proline (1 g, 4.012 mmol), triethylamine (0.562 mL, 4.012 mmol), ethyl chloroformate (0.395 mL, 4.012 mmol) and 4-*tert*-butylaniline (0.645 mL, 4.012 mmol) afforded **12** as a yellow solid (yield 56%). No purification was needed.

^{1}H-NMR (500 MHz, $CDCl_3$): 1.31 (s, 9H), 1.76-2.60 (m, 4H), 3.36-3.71 (m, 2H), 4.51 (s, 1H), 5.02-5.33 (m, 2H), 7.06-7.58 (m, 9H), 7.75 and 9.09 (s, 1H).
^{13}C-NMR (125 MHz, $CDCl_3$): δ 24.8, 27.7, 31.5, 34.4, 47.2, 61.2, 67.7, 119.7, 125.7, 125.9, 128.0, 128.3, 128.7, 135.6, 136.4, 156.9, 169.4.
IR (ATR): ν_{max} (cm^{-1}) 1410, 1446, 1519, 1535, 1604, 1671, 1696, 2872, 2901, 2959, 3307.
ESI-MS (CH_3OH): m/z calcd for $C_{23}H_{28}N_2O_3$ 381.2178 [M+H$^+$], found 381.2178.
Melting point: 48-52 °C.
[α]$^{25}_D$ (CH_2Cl_2): -109.64.

Synthesis of catalyst 13

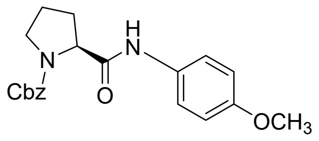

13

According to the general procedure 3.4., Cbz-L-proline (6 g, 24.071 mmol), triethylamine (3.370 mL, 24.071 mmol), ethyl chloroformate (2.373 mL, 24.071 mmol) and 4-methoxyaniline (2.995 g, 24.071 mmol) afforded **13** as a purple solid (yield 63%). Purification by crystallization in CH_2Cl_2/*n*-hexane.

^{1}H-NMR (500 MHz, $CDCl_3$): δ 1.80-2.58 (m, 4H), 3.37-3.66 (m, 2H), 3.77 (s, 3H), 4.48 (s, 1H), 4.94-5.34 (m, 2H), 6.80 (d, J = 8.7 Hz, 2H), 7.11-7.55 (m, 7H), 7.70 and 9.01 (s, 1H).
^{13}C-NMR (125 MHz, $CDCl_3$): δ 24.8, 27.9, 47.2, 55.6, 61.1, 67.6, 114.1, 121.5, 128.0, 128.3, 128.7, 130.5, 131.5, 136.4, 156.3, 169.3.
IR (ATR): ν_{max} (cm^{-1}) 1413, 1446, 1509, 1535, 1658, 1699, 1714, 1736, 1782, 1814, 2885, 2949, 2973, 3289.
ESI-MS (CH_3OH): m/z calcd for $C_{20}H_{22}N_2O_4$ 355.16 [M+H$^+$], found 355.2.
Melting point: 122-124 °C.
[α]$^{25}_D$ (CH_2Cl_2): -123.90.

Synthesis of catalyst 14

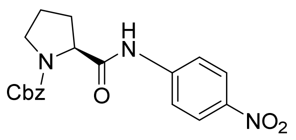

14

According to the general procedure 3.4., Cbz-L-proline (1 g, 4.012 mmol), triethylamine (0.562 mL, 4.012 mmol), ethyl chloroformate (0.395 mL, 4.012 mmol) and 4-nitroaniline (0.559 g, 4.012 mmol) afforded **14** as a yellow solid (yield 31%). Purification by crystallization in IPA.

^{1}H-NMR (500 MHz, $CDCl_3$): δ 1.89-2.14 (m, 3H), 2.31-2.46 (m, 1H), 3.44-3.57 (s, 1H), 3.58-3.67 (m, 1H), 4.53 (s, 1H), 5.14-5.31 (m, 2H), 7.26-7.44 (m, 5H), 7.50 (d, J = 8.2 Hz, 2H), 8.00 (d, J = 7.4 Hz, 2H), 8.16 and 9.88 (s, 1H).
^{13}C-NMR (125 MHz, $CDCl_3$): δ 24.7, 28.2, 47.4, 61.3, 67.9, 119.1, 124.8, 127.9, 128.5, 128.7, 136.2, 143.3, 144.2, 156.9, 170.5.
IR (ATR): ν_{max} (cm^{-1}) 1408, 1422, 1450, 1495, 1508, 1553, 1595, 1614, 1662, 1707, 2877, 2961, 3033, 3066, 3090, 3158, 3219, 3247, 3282.
ESI-MS (CH_3OH): m/z calcd for $C_{19}H_{19}N_3O_5$ 370.14 [M+H$^+$], found 370.1.
Melting point: 162-164 °C.
[α]$^{25}_D$ (CH_2Cl_2): -106.02.

Synthesis of catalyst 15

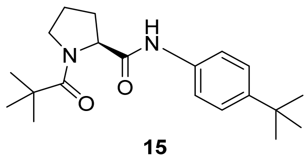

15

According to the general procedure 3.4., Piv-L-proline (3 g, 15.056 mmol), triethylamine (2.108 mL, 15.056 mmol), ethyl chloroformate (1.484 mL, 15.056 mmol) and 4-*tert*-butylaniline (2.421 mL, 15.056 mmol) afforded **15** as a white solid (yield 45%). Purification by crystallization in CH_2Cl_2/*n*-hexane.

^{1}H-NMR (500 MHz, $CDCl_3$): δ 1.26 (s, 9H), 1.30 (s, 9H), 1.83-2.01 (m, 2H), 2.13-2.25 (m, 1H), 2.29-2.38 (m, 1H), 3.66-3.81 (m, 2H), 4.81 (dd, J = 8.1, 3.2 Hz, 1H), 7.20-7.25 (m, 2H), 7.36-7.42 (m, 2H), 9.33 (s, 1H).
^{13}C-NMR (125 MHz, $CDCl_3$): δ 26.0, 26.5, 27.7, 31.5, 34.3, 39.3, 48.5, 62.5, 119.6, 125.5, 135.9, 146.6, 170.1, 178.3.
IR (ATR): ν_{max} (cm^{-1}) 1415, 1478, 1518, 1540, 1601, 1686, 2871, 2906, 2968, 3131, 3202, 3281, 3311.
ESI-MS (CH_3OH): m/z calcd for $C_{20}H_{30}N_2O_2$ 331.23 [M+H$^+$], found 331.4.
Melting point: 132-134 °C.
[α]$^{25}_D$ (CH_2Cl_2): -102.26.

Synthesis of catalyst 16

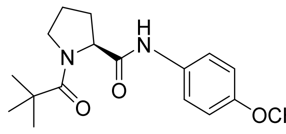

16

According to the general procedure 3.4., Piv-L-proline (3 g, 15.056 mmol), triethylamine (2.108 mL, 15.056 mmol), ethyl chloroformate (1.484 mL, 15.056 mmol) and 4-methoxyaniline (1.873 g, 15.056 mmol) afforded **16** as an orange solid (yield 44%). Purification by column chromatography (silica, CH_2Cl_2/CH_3OH = 95/5).

^{1}**H-NMR** (500 MHz, $CDCl_3$): δ 1.28 (s, 9H), 1.83-1.99 (m, 2H), 2.11-2.23 (m, 1H), 2.23-2.32 (m, 1H), 3.66-3.78 (m, 5H), 4.75 (dd, J = 8.1, 3.4 Hz, 1H), 6.65-6.73 (m, 2H), 7.31-7.38 (m, 2H), 9.29 (s, 1H).

^{13}**C-NMR** (125 MHz, $CDCl_3$): δ 26.0, 26.7, 27.6, 39.3, 48.5, 55.5, 62.5, 113.9, 121.2, 131.9, 155.9, 170.1, 178.1.

IR (ATR): ν_{max} (cm^{-1}) 1402, 1415, 1436, 1448, 1459, 1479, 1512, 1523, 1546, 1597, 1625, 1671, 1740, 2841, 2884, 2908, 2919, 2935, 2972, 2979, 3358.

ESI-MS (CH_3OH): m/z calcd for $C_{17}H_{24}N_2O_3$ 305.18 [M+H$^+$], found 305.3.

Melting point: 96-98 °C.

[α]$^{25}_D$ (CH_2Cl_2): -100.96.

4. NMR spectra

Catalyst 3

^{1}H-NMR (500 MHz, $CDCl_3$, rt)

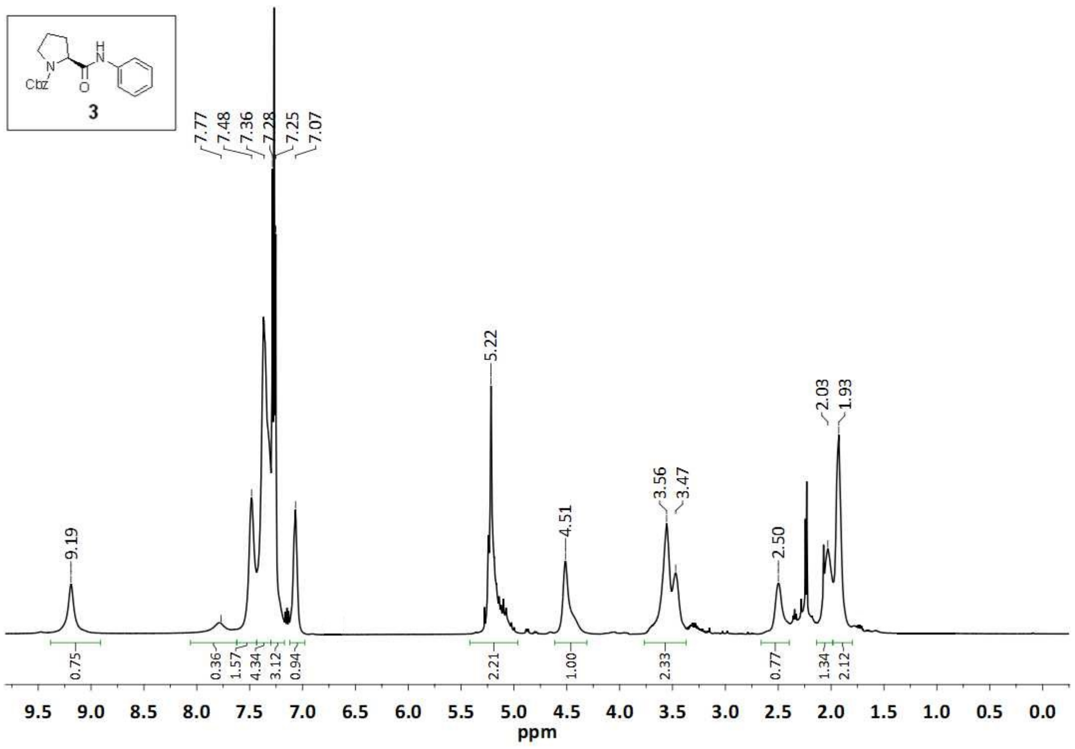

^{13}C-NMR (125 MHz, $CDCl_3$, rt)

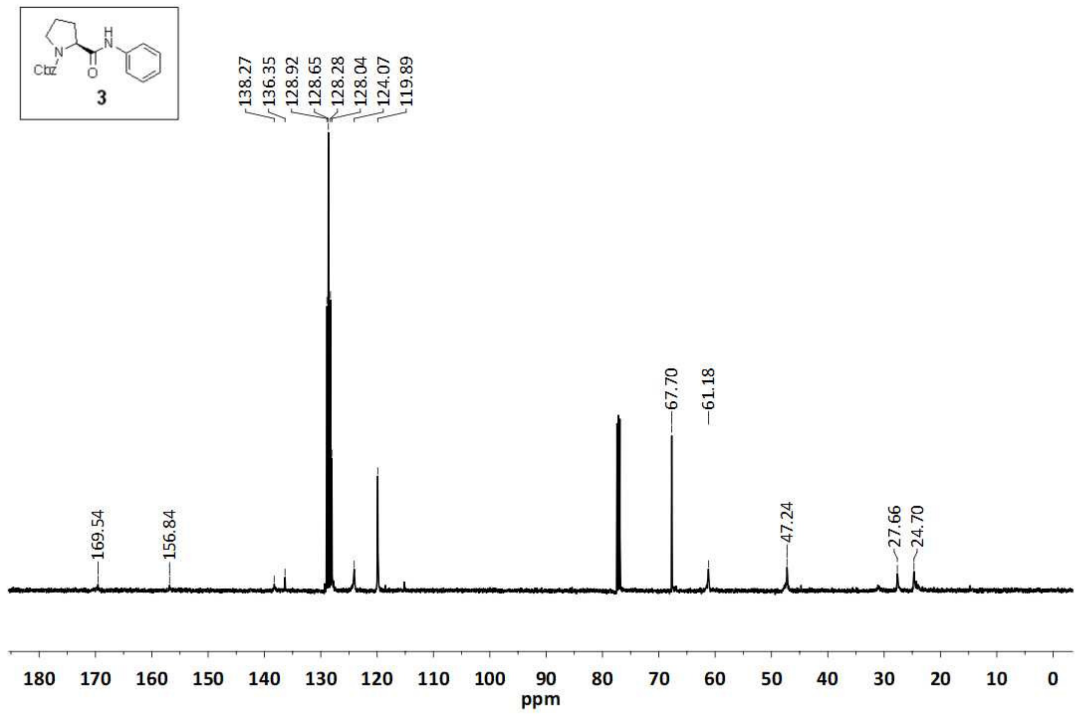
Cbz
3
138.27
136.35
128.92
128.65
128.28
128.04
124.07
119.89
67.70
61.18
169.54
156.84
47.24
27.66
24.70
180 170 160 150 140 130 120 110 100 90 80 70 60 50 40 30 20 10 0
ppm

Catalyst 4

^{1}H-NMR (500 MHz, $CDCl_3$, rt)

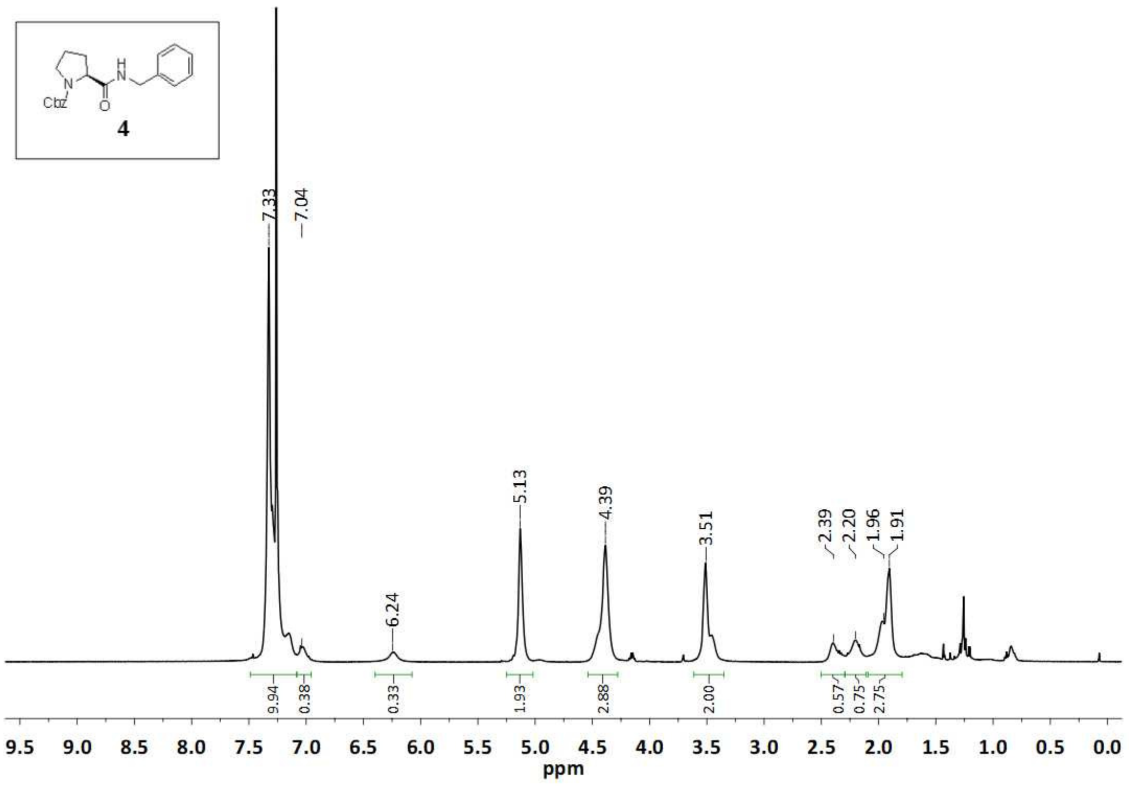

^{13}C-NMR (125 MHz, $CDCl_3$, rt)

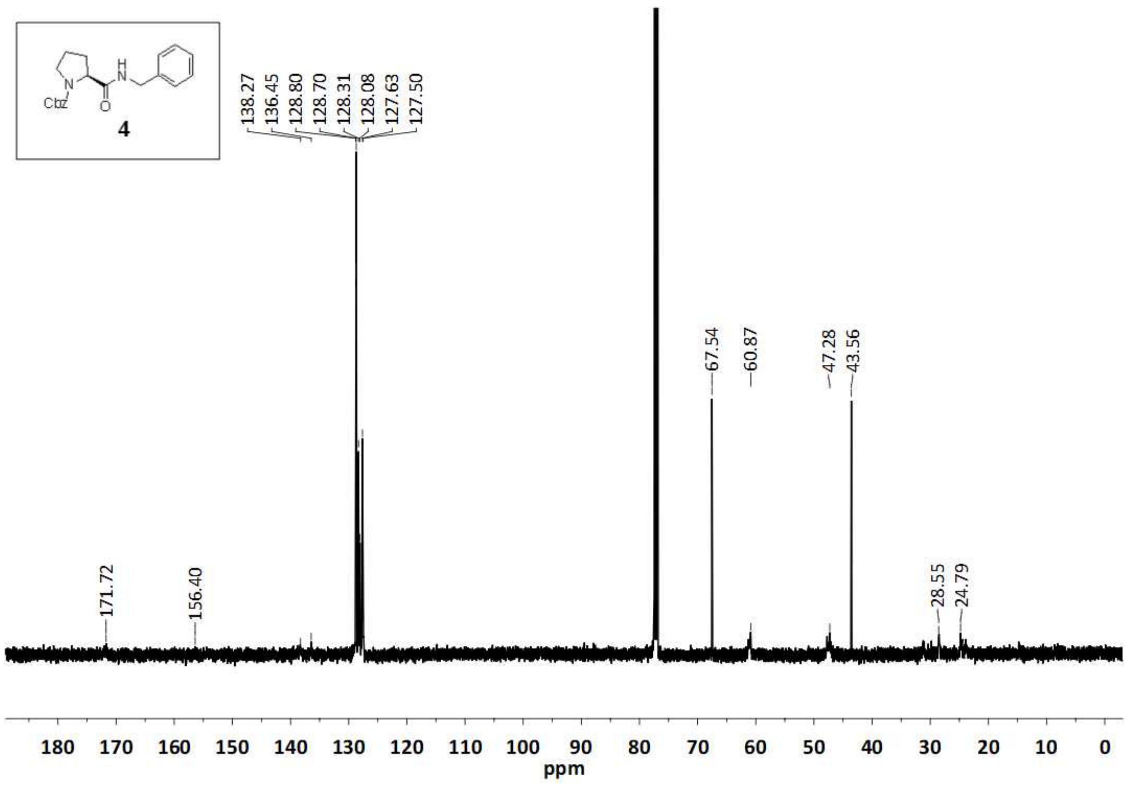
Cbz
4
138.27
136.45
128.80
128.70
128.31
128.08
127.63
127.50
67.54
60.87
47.28
43.56
171.72
156.40
28.55
24.79
180 170 160 150 140 130 120 110 100 90 80 70 60 50 40 30 20 10 0
ppm

Catalyst 5

^{1}H-NMR (500 MHz, $CDCl_3$, rt)

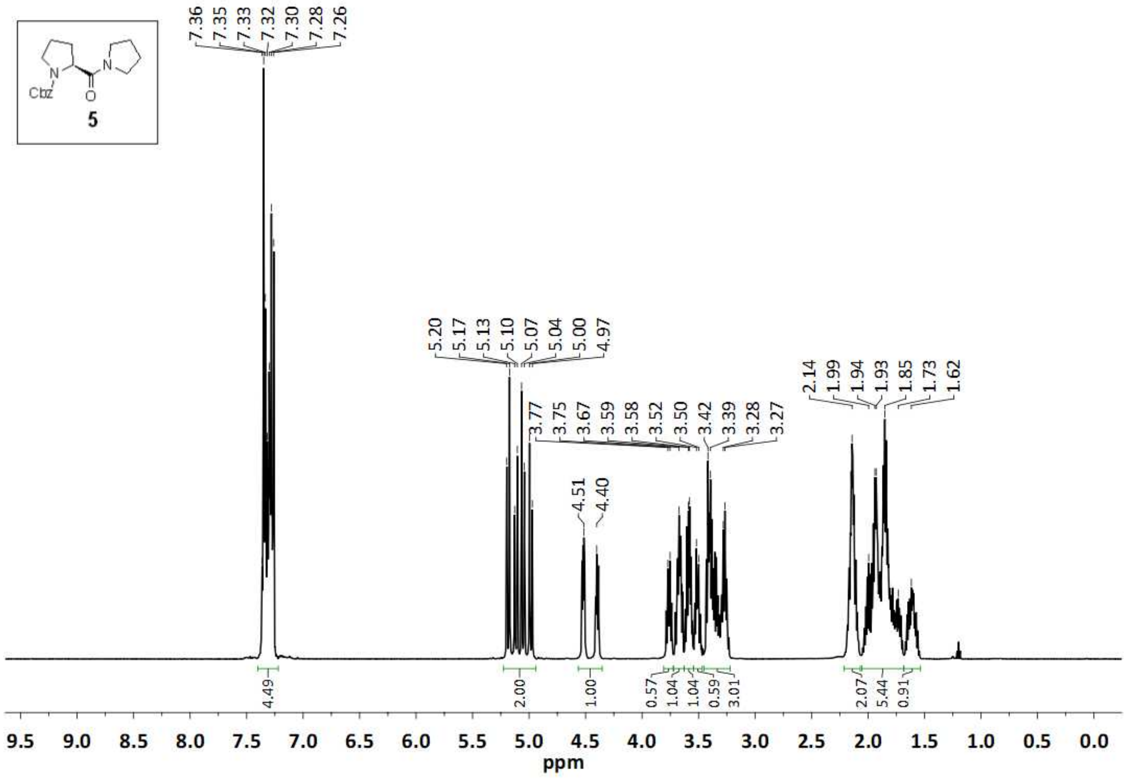

^{13}C-NMR (125 MHz, $CDCl_3$, rt)

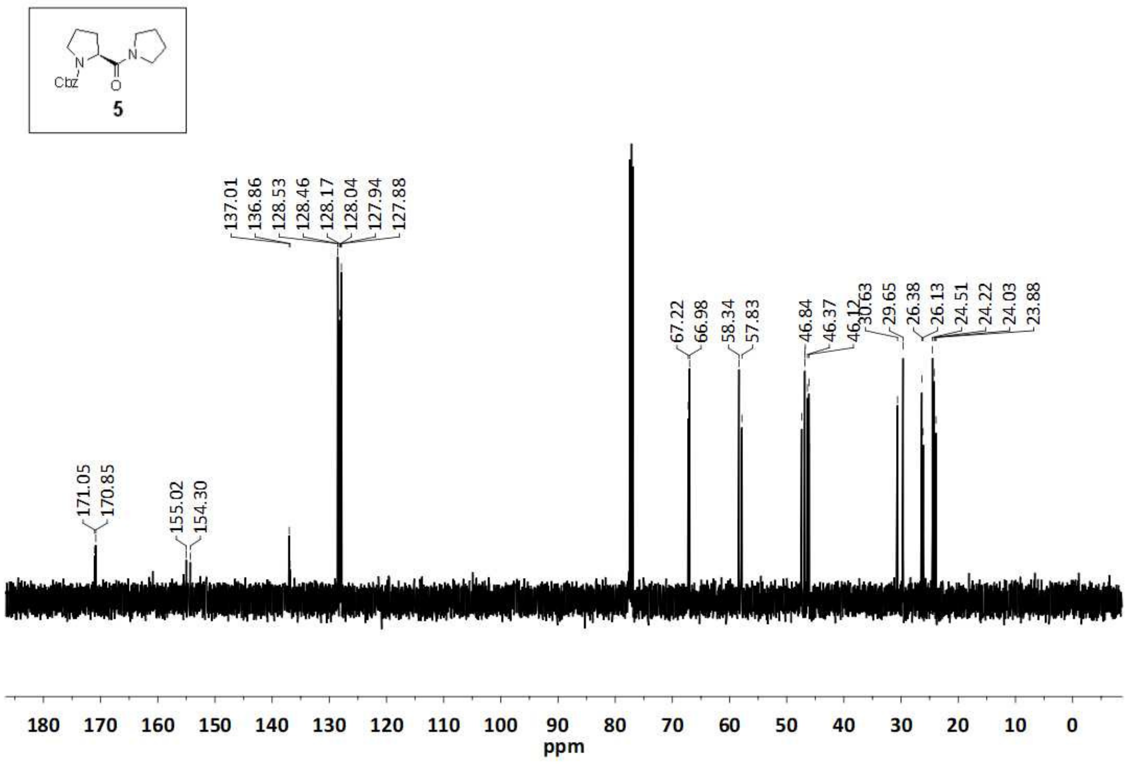

Catalyst 6

^{1}H-NMR (500 MHz, $CDCl_3$, rt)

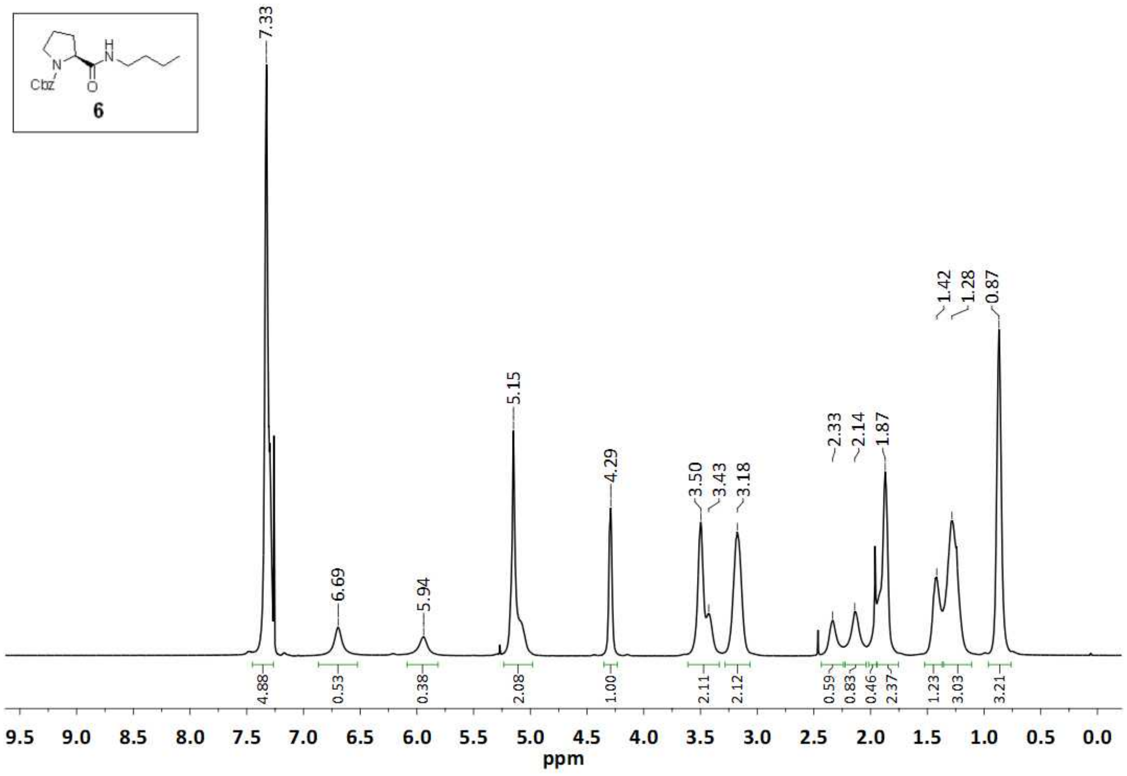

^{13}C-NMR (125 MHz, $CDCl_3$, rt)

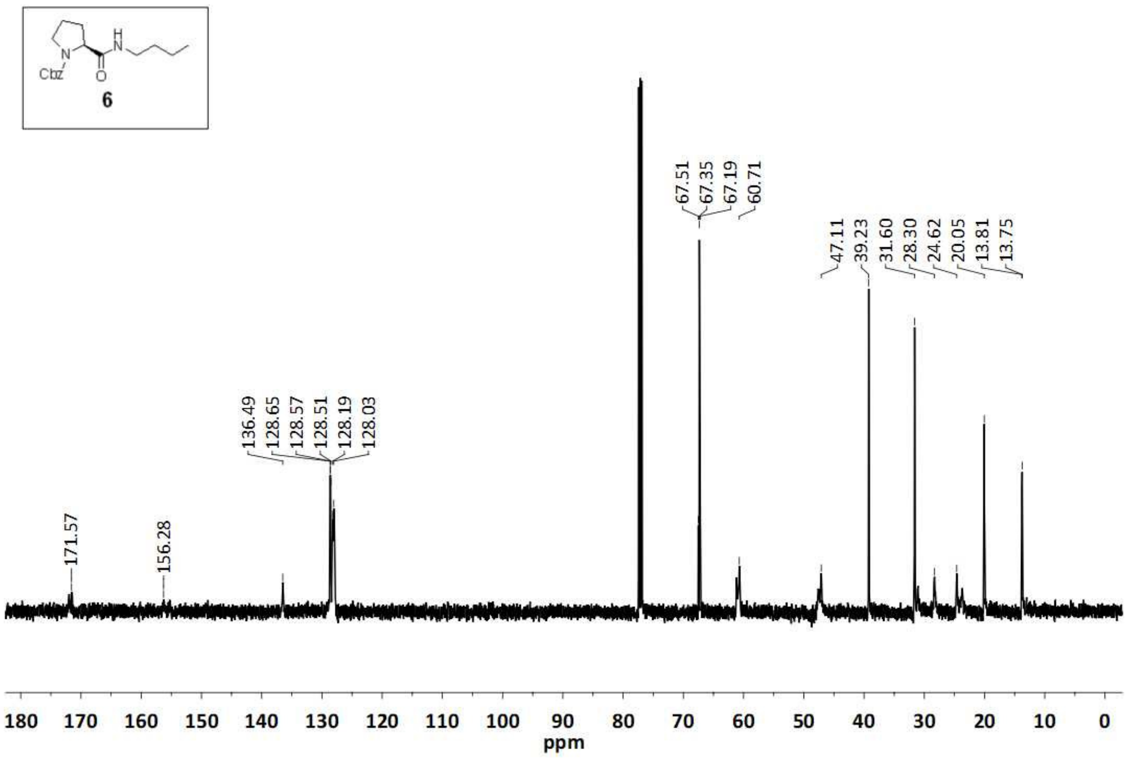
Cbz
6
171.57
156.28
136.49
128.65
128.57
128.51
128.19
128.03
67.51
67.35
67.19
60.71
47.11
39.23
31.60
28.30
24.62
20.05
13.81
13.75
180 170 160 150 140 130 120 110 100 90 80 70 60 50 40 30 20 10 0
ppm

Catalyst 7

^{1}H-NMR (500 MHz, $CDCl_3$, rt)

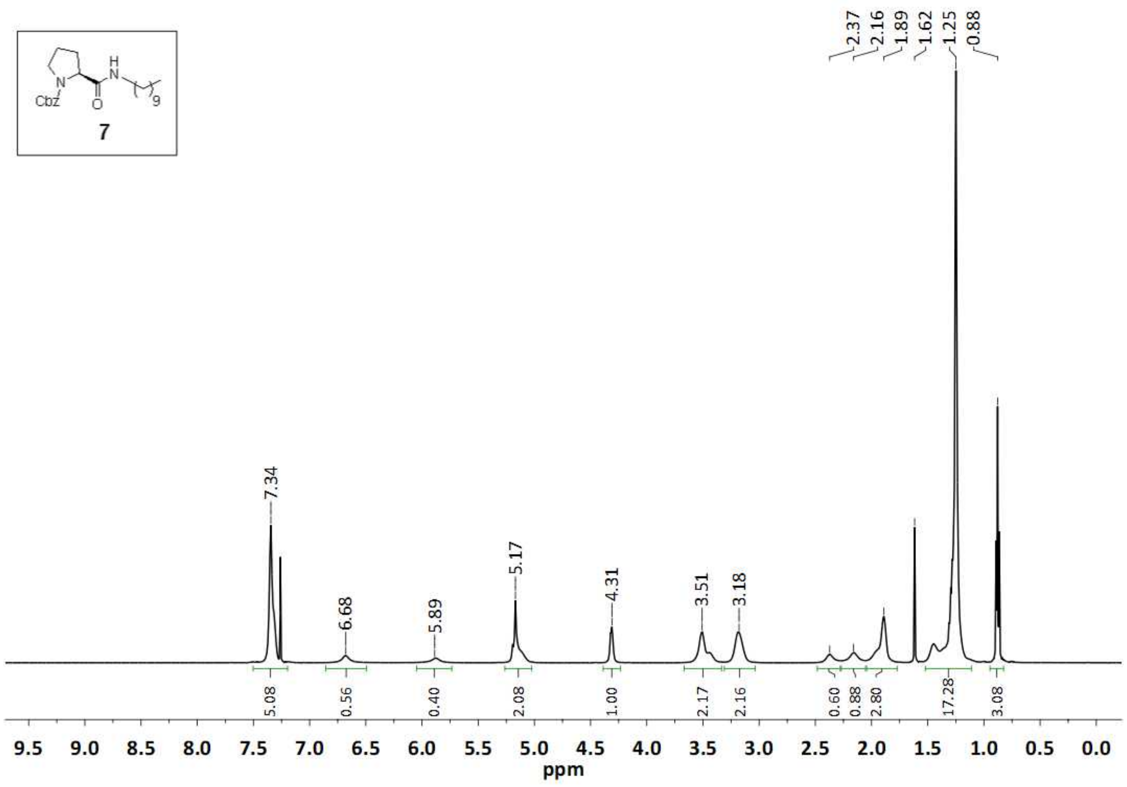

^{13}C-NMR (125 MHz, $CDCl_3$, rt)

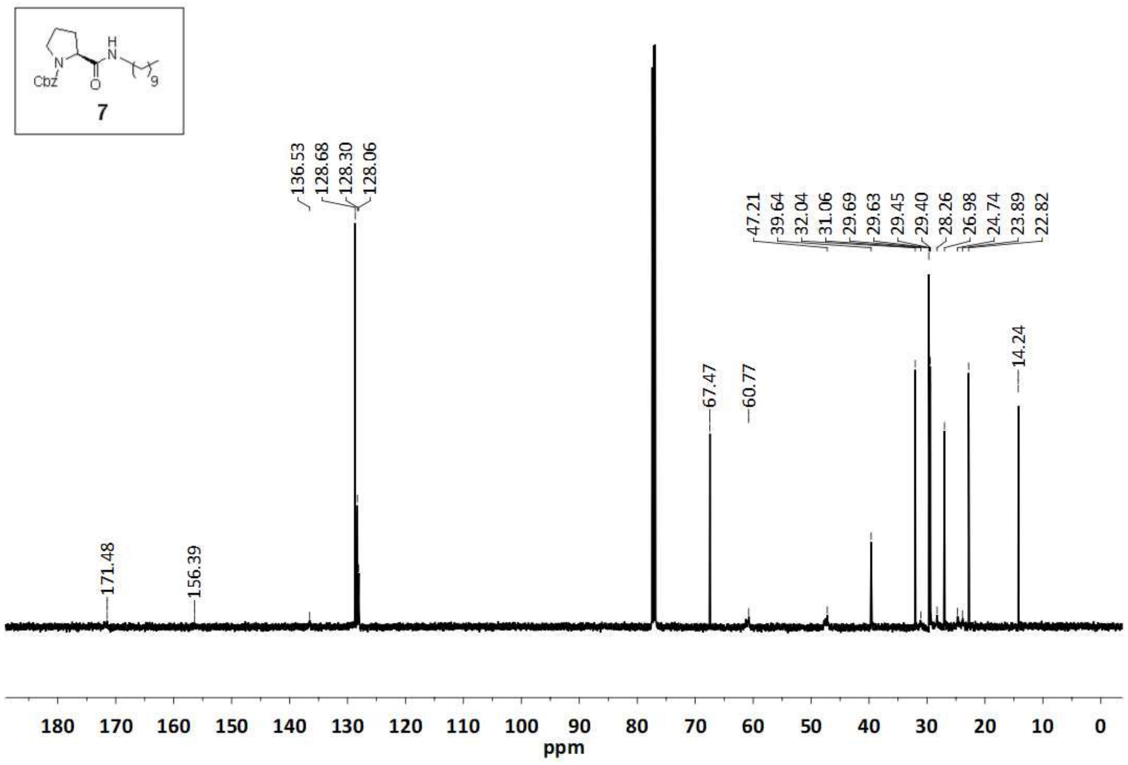
Cbz
7
136.53
128.68
128.30
128.06
47.21
39.64
32.04
31.06
29.69
29.63
29.45
29.40
28.26
26.98
24.74
23.89
22.82
14.24
67.47
60.77
171.48
156.39
180 170 160 150 140 130 120 110 100 90 80 70 60 50 40 30 20 10 0
ppm

Catalyst 8

^{1}H-NMR (500 MHz, $CDCl_3$, rt)

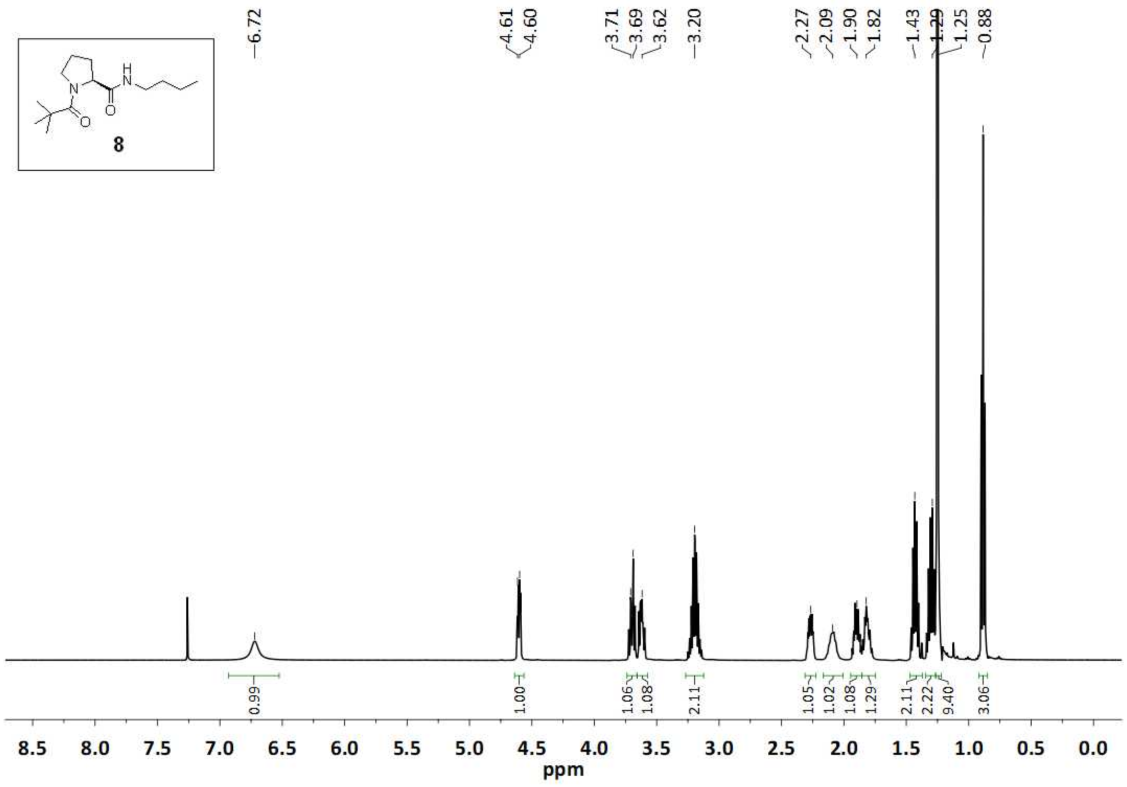

^{13}C-NMR (125 MHz, $CDCl_3$, rt)

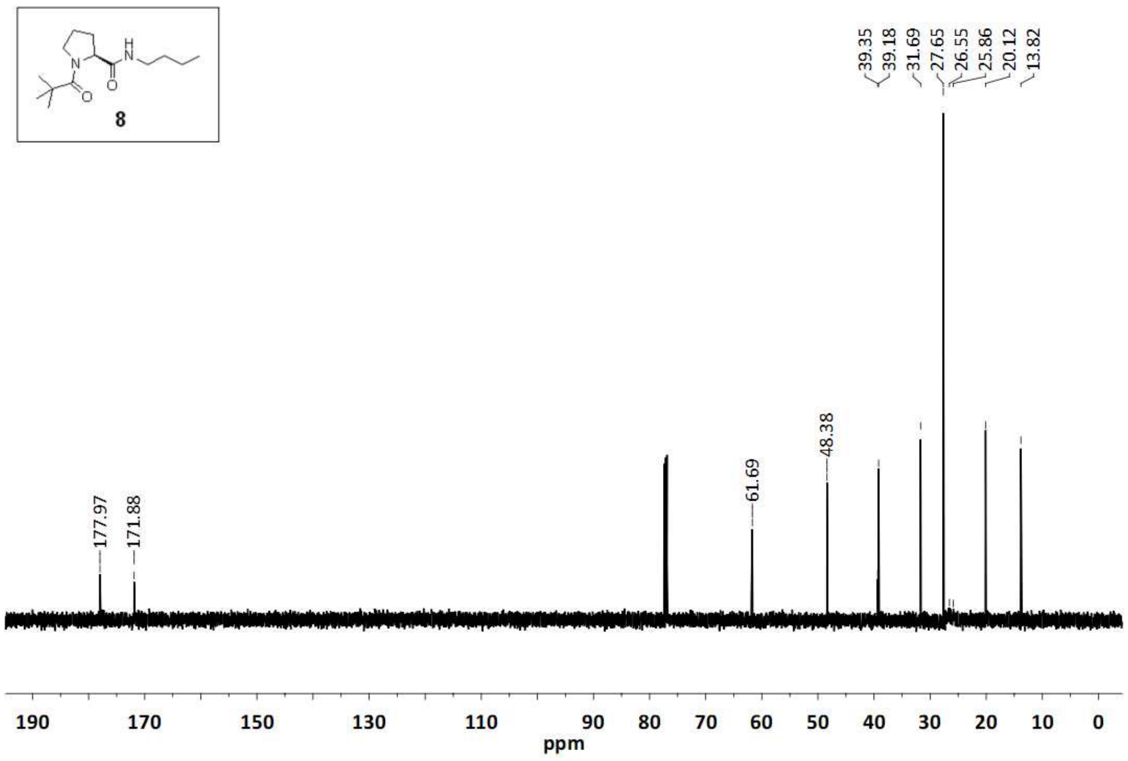
8
39.35
39.18
31.69
27.65
26.55
25.86
20.12
13.82
48.38
61.69
177.97
171.88
190
170
150
130
110
90
80
70
60
50
40
30
20
10
0
ppm

Catalyst 9

^{1}H-NMR (500 MHz, $CDCl_3$, rt)

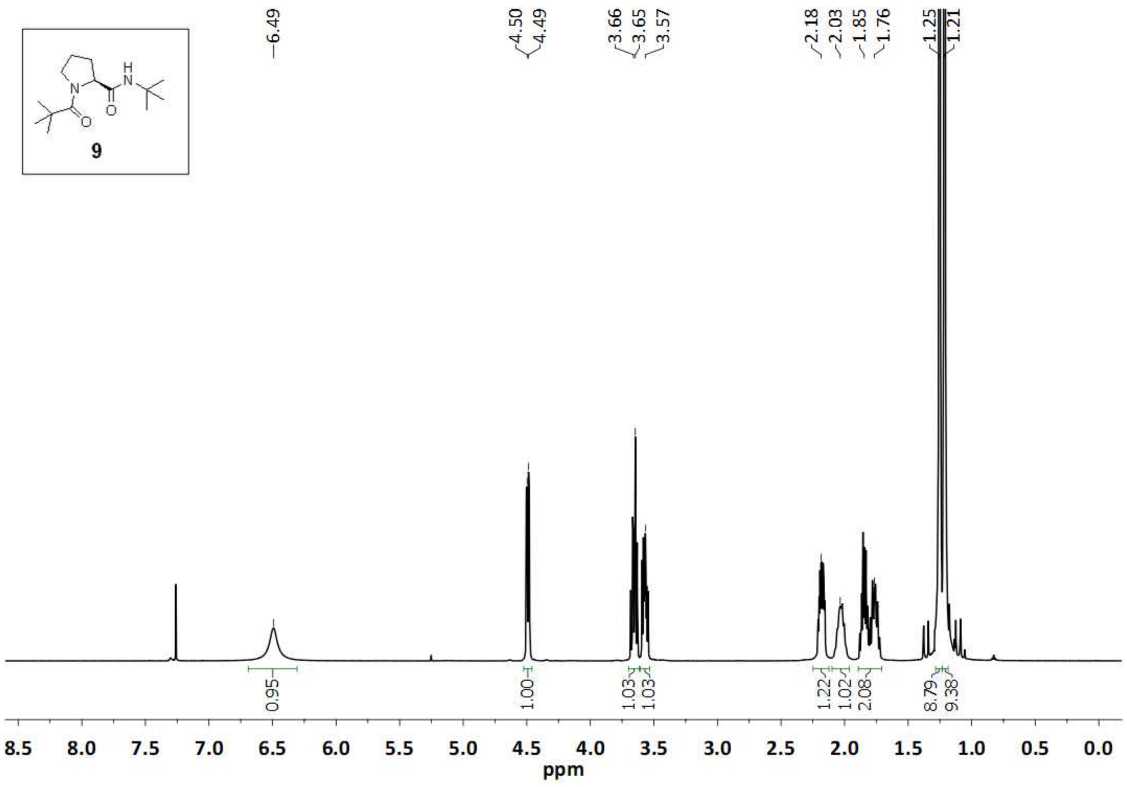

^{13}C-NMR (125 MHz, $CDCl_3$, rt)

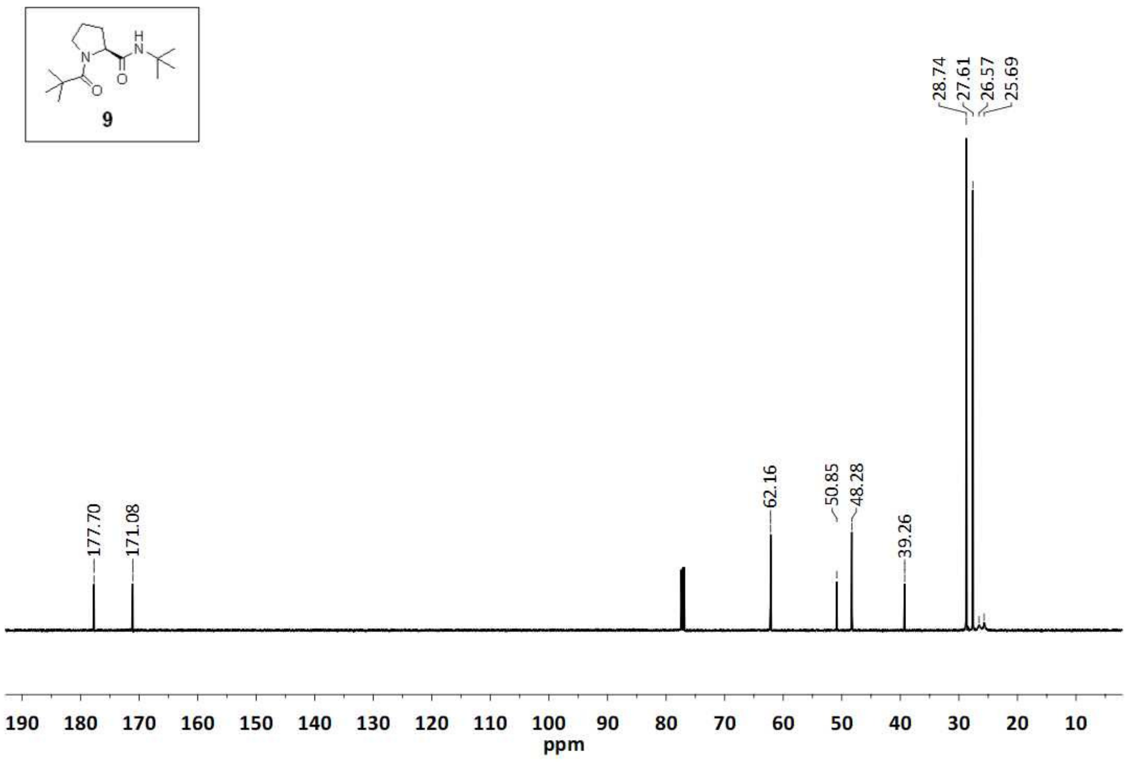
9
28.74
27.61
26.57
25.69
177.70
171.08
62.16
50.85
48.28
39.26
190 180 170 160 150 140 130 120 110 100 90 80 70 60 50 40 30 20 10
ppm

Catalyst 10

^{1}H-NMR (500 MHz, $CDCl_3$, rt)

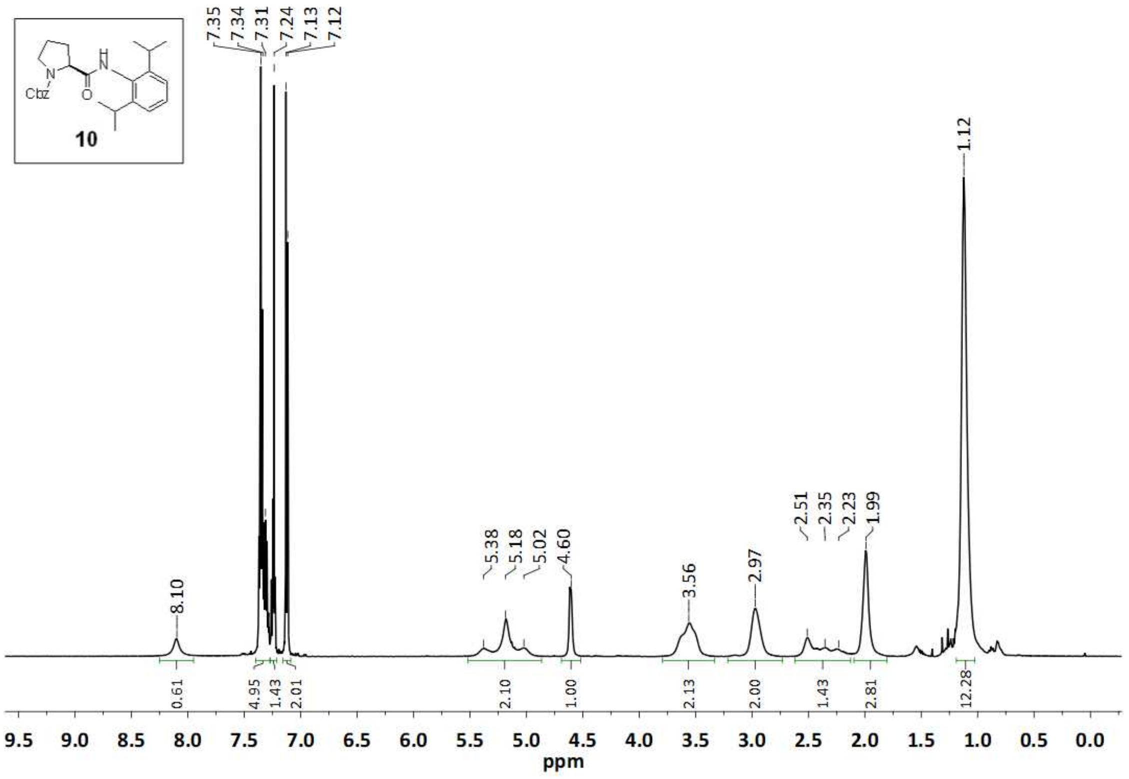

^{13}C-NMR (125 MHz, $CDCl_3$, rt)

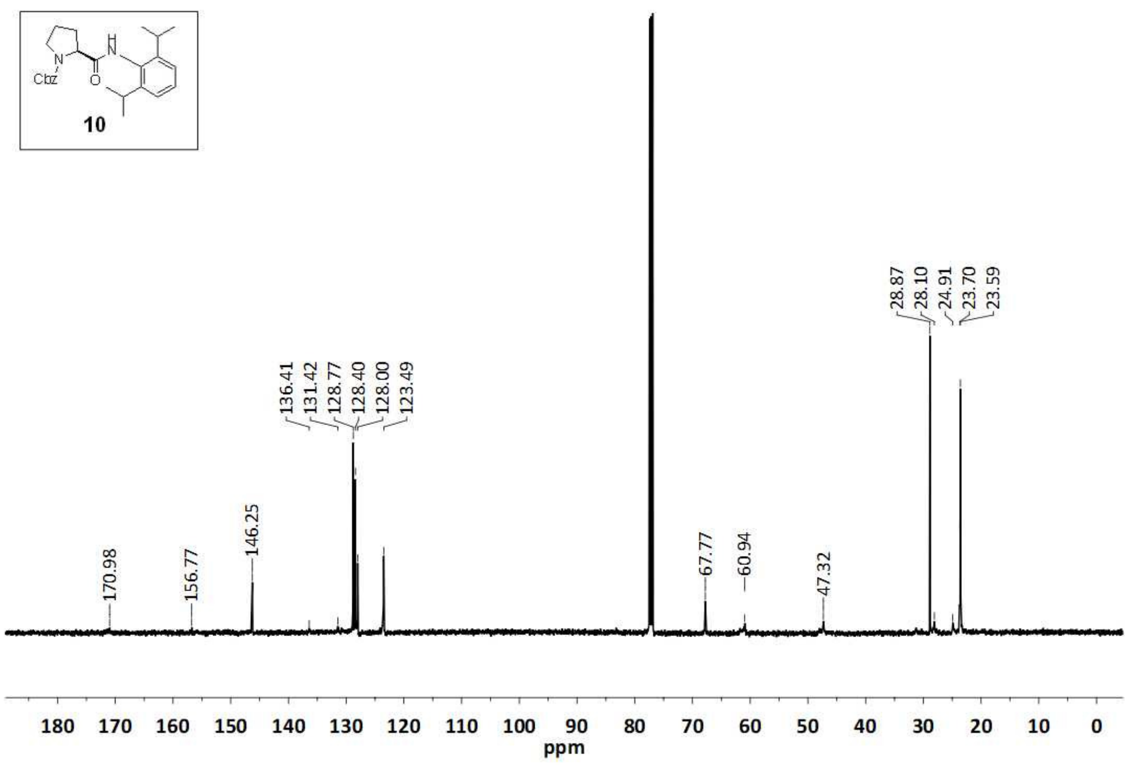
Cbz
10
170.98
156.77
146.25
136.41
131.42
128.77
128.40
128.00
123.49
67.77
60.94
47.32
28.87
28.10
24.91
23.70
23.59
180 170 160 150 140 130 120 110 100 90 80 70 60 50 40 30 20 10 0
ppm

Catalyst 11

^{1}H-NMR (500 MHz, $CDCl_3$, rt)

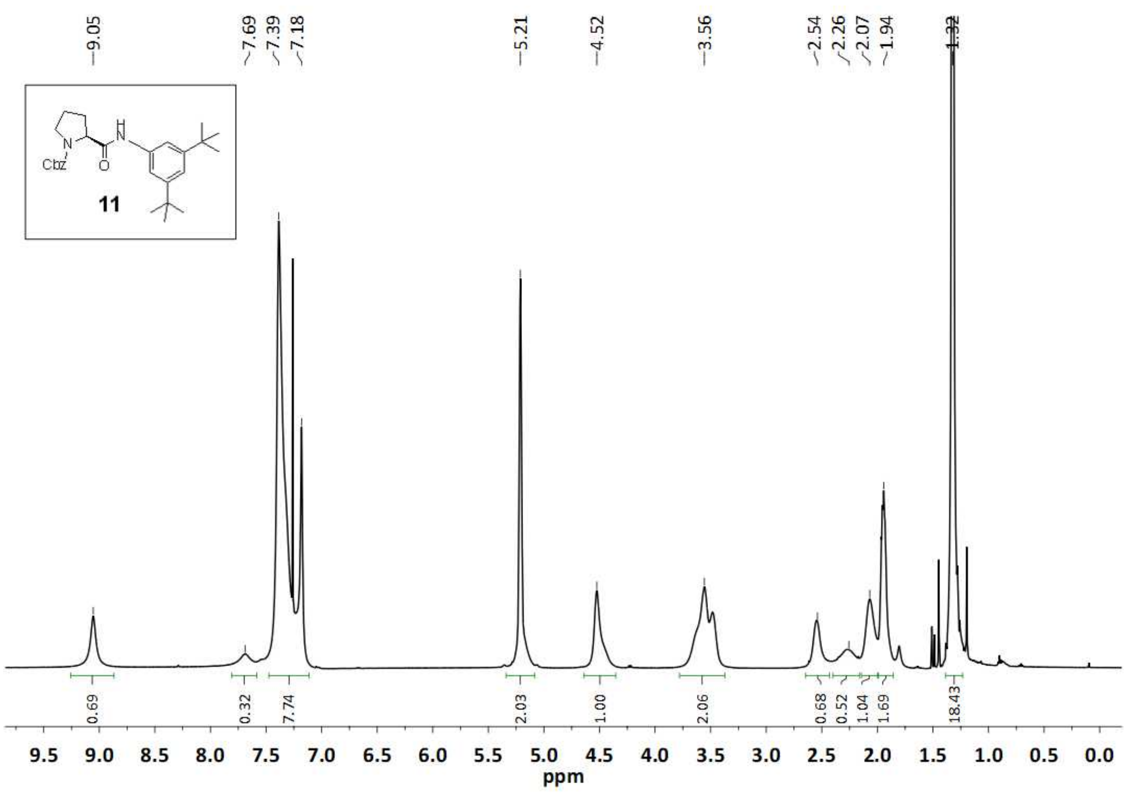

^{13}C-NMR (125 MHz, $CDCl_3$, rt)

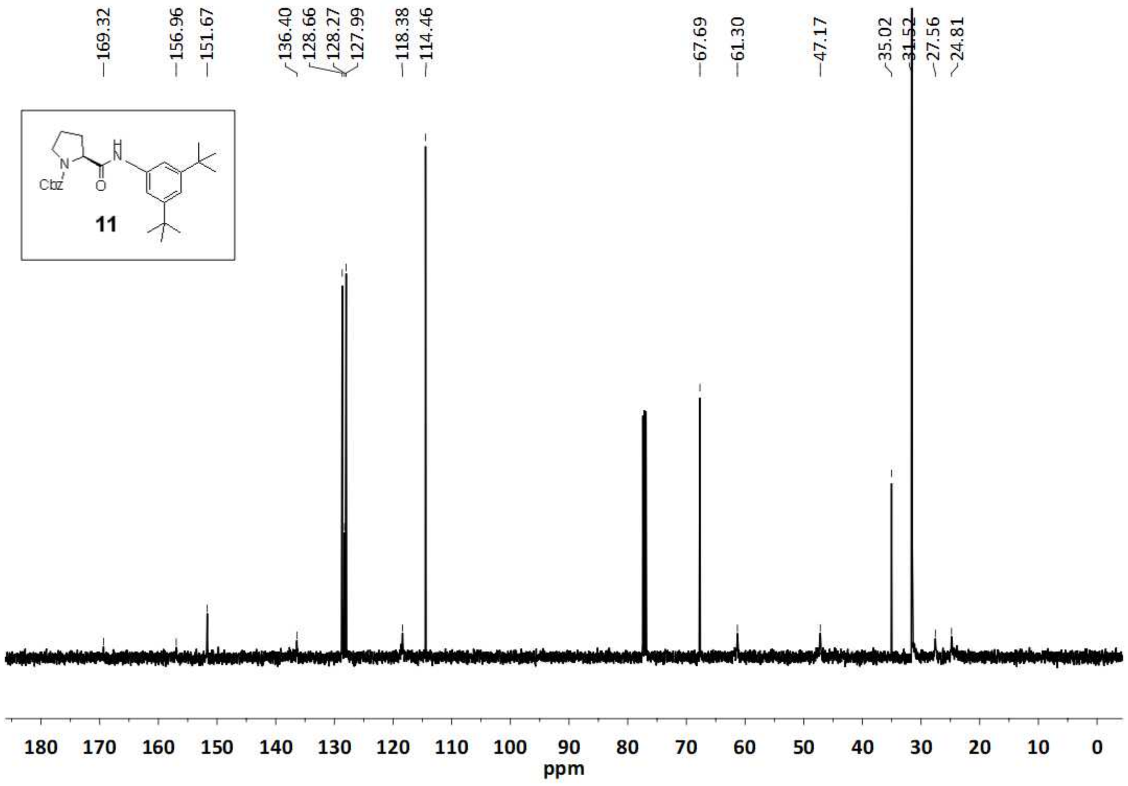
169.32
156.96
151.67
136.40
128.66
128.27
127.99
118.38
114.46
67.69
61.30
47.17
35.02
31.52
27.56
24.81
Cbz
N
H
O
11
180 170 160 150 140 130 120 110 100 90 80 70 60 50 40 30 20 10 0
ppm

Catalyst 12

^{1}H-NMR (500 MHz, $CDCl_3$, rt)

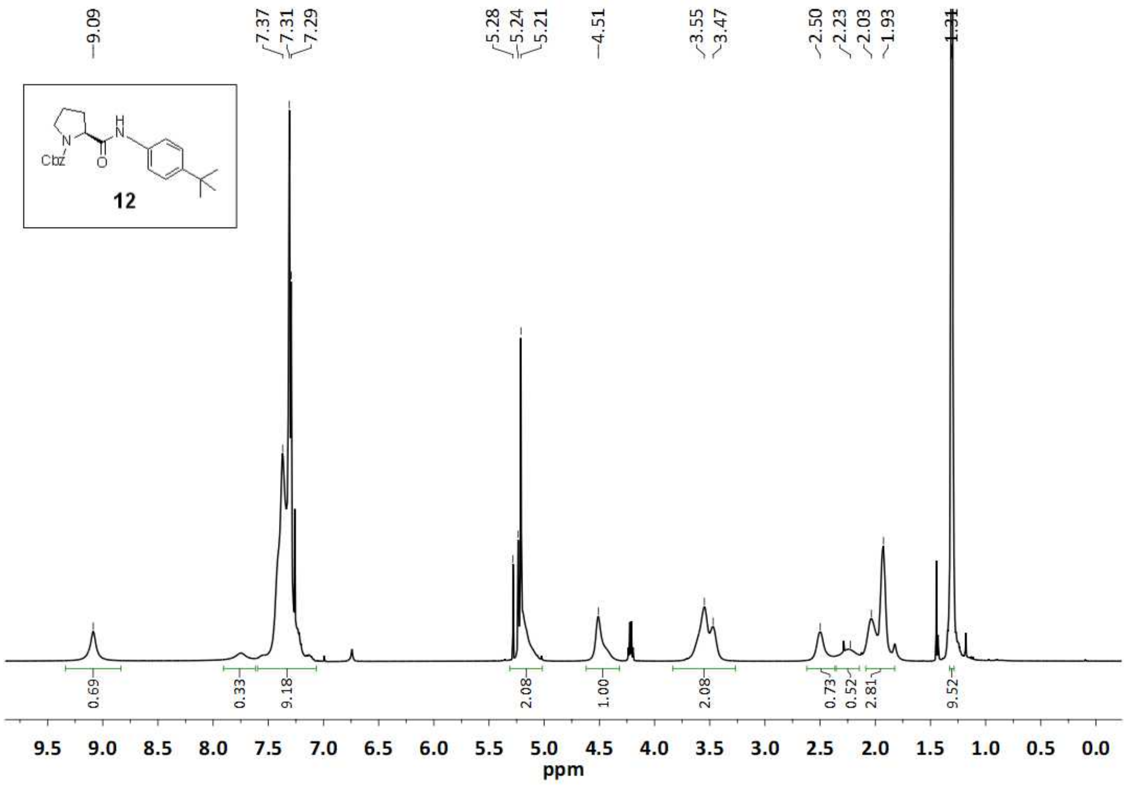

^{13}C-NMR (125 MHz, $CDCl_3$, rt)

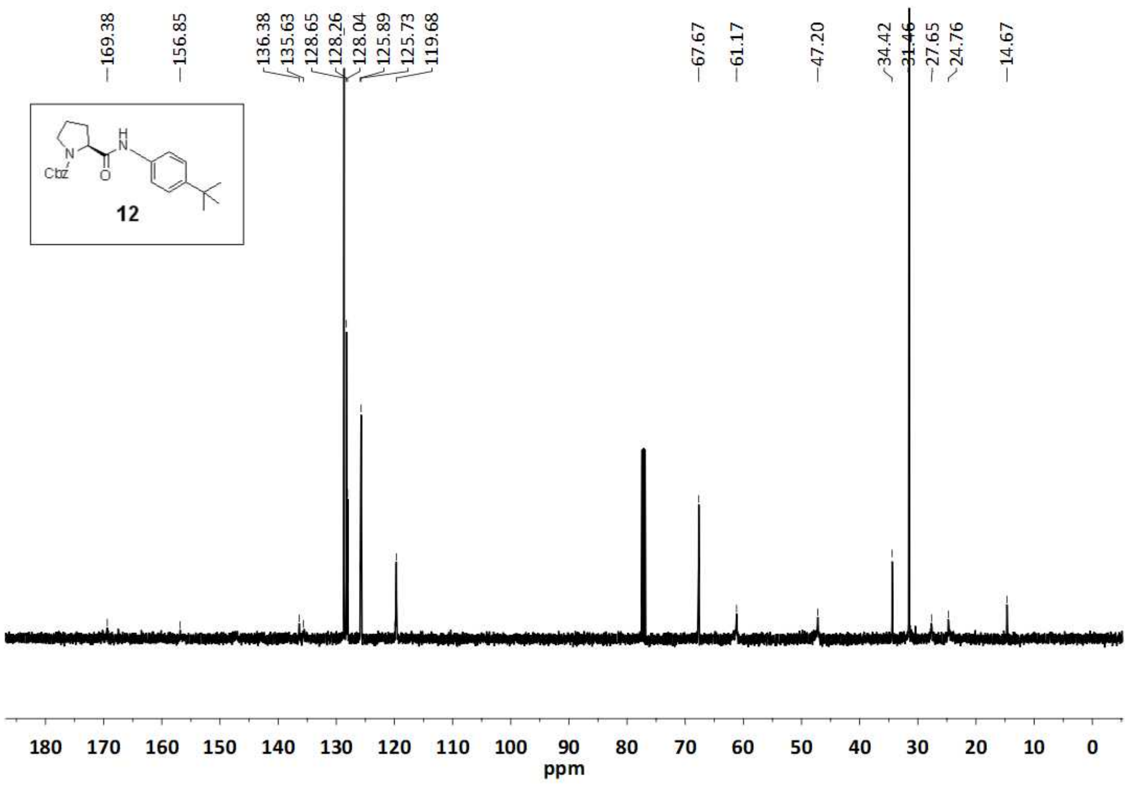

Catalyst 13

^{1}H-NMR (500 MHz, $CDCl_3$, rt)

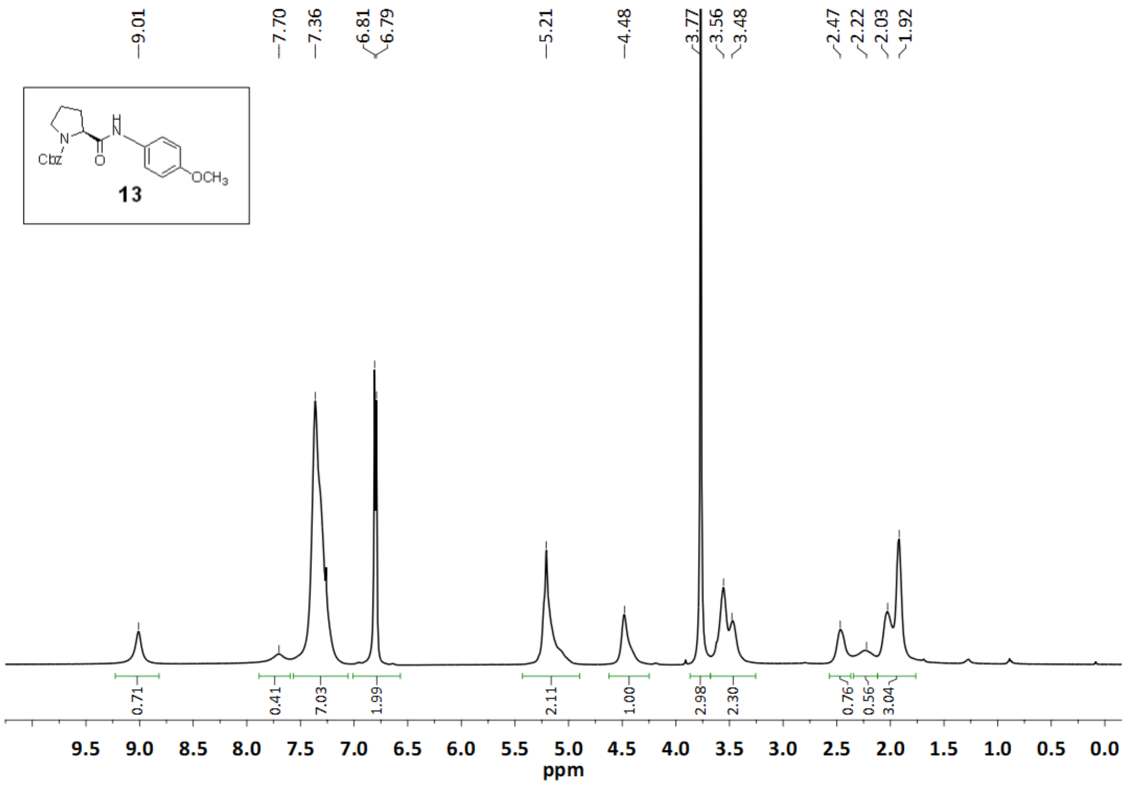

^{13}C-NMR (125 MHz, $CDCl_3$, rt)

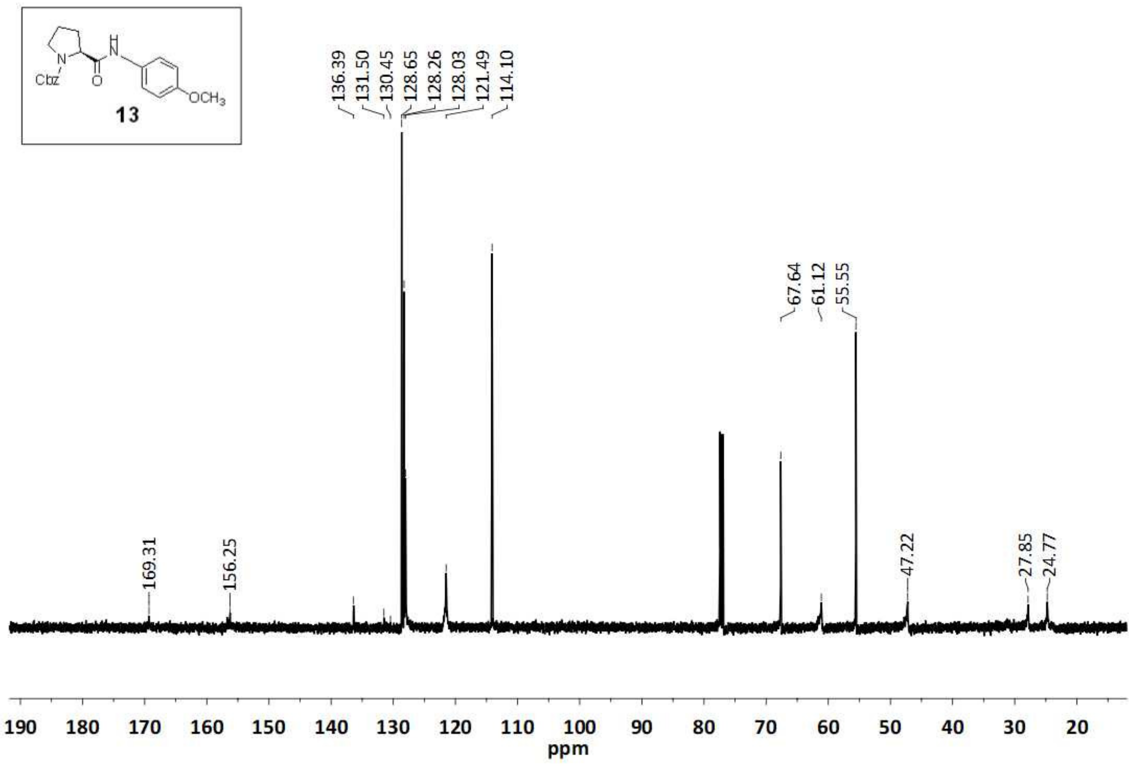
Cbz
13
OCH3
136.39
131.50
130.45
128.65
128.26
128.03
121.49
114.10
67.64
61.12
55.55
169.31
156.25
47.22
27.85
24.77
190 180 170 160 150 140 130 120 110 100 90 80 70 60 50 40 30 20
ppm

Catalyst 14

^{1}H-NMR (500 MHz, $CDCl_3$, rt)

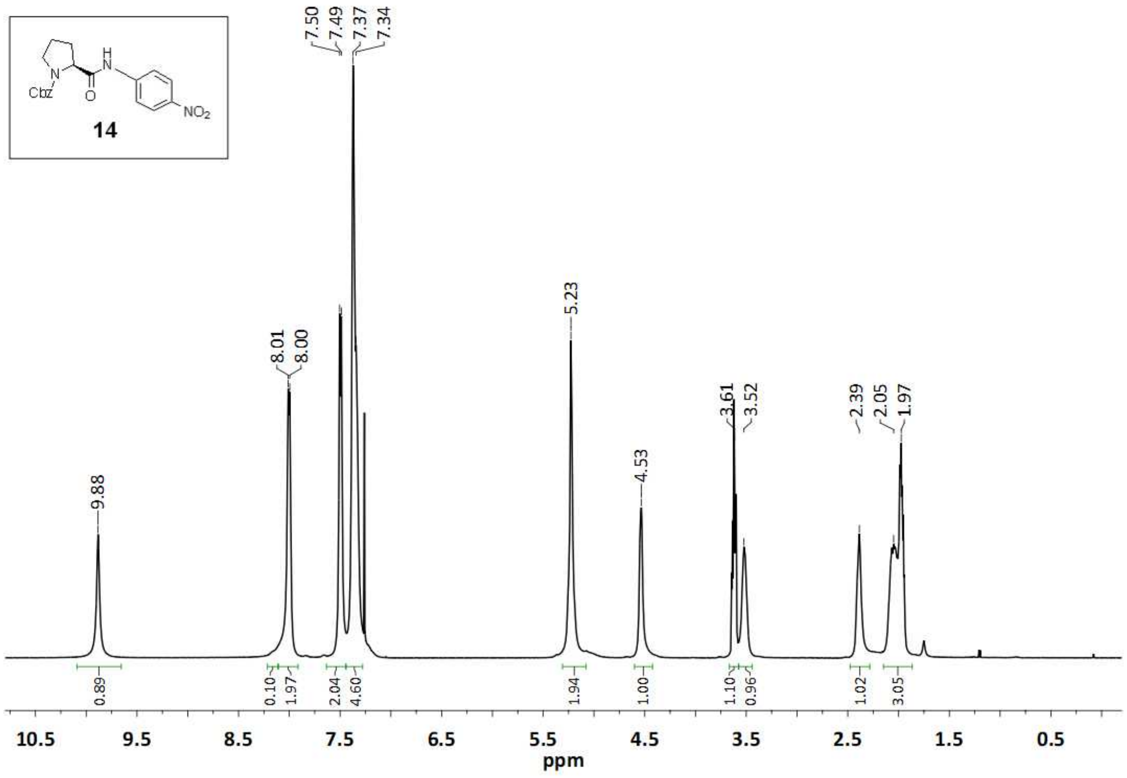

^{13}C-NMR (125 MHz, $CDCl_3$, rt)

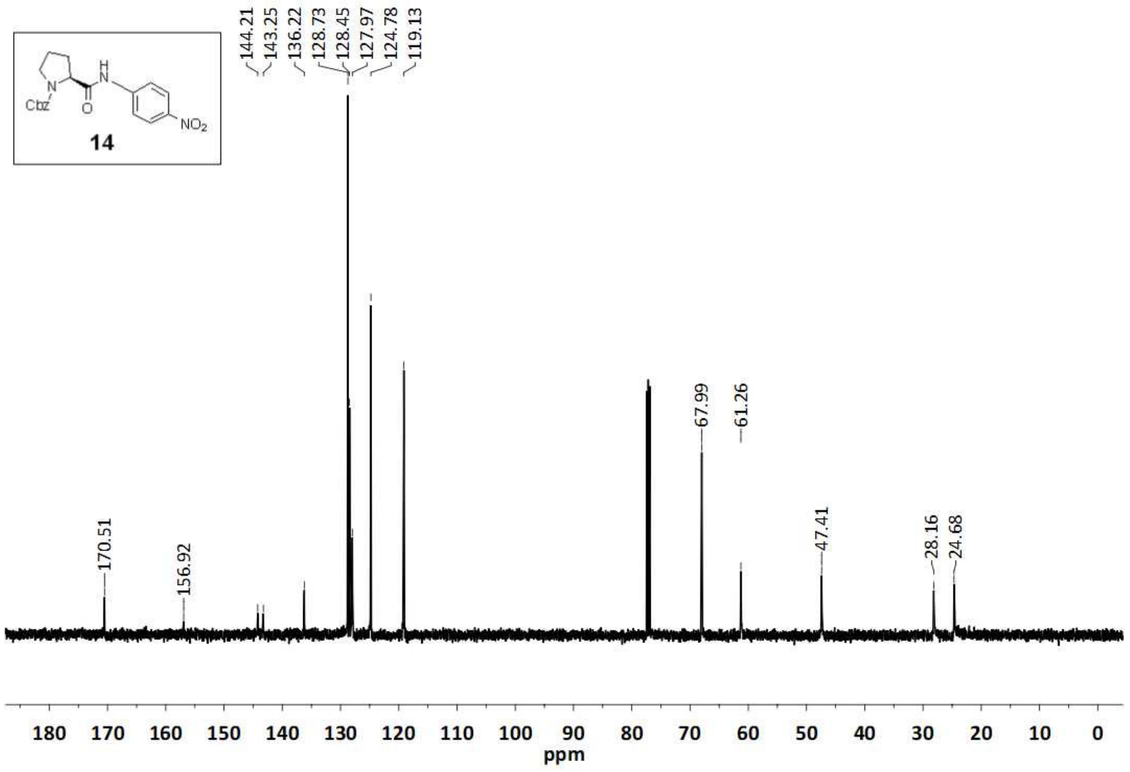
144.21
143.25
136.22
128.73
128.45
127.97
124.78
119.13
14
67.99
61.26
170.51
156.92
47.41
28.16
24.68
180 170 160 150 140 130 120 110 100 90 80 70 60 50 40 30 20 10 0
ppm

Catalyst 15

^{1}H-NMR (500 MHz, $CDCl_3$, rt)

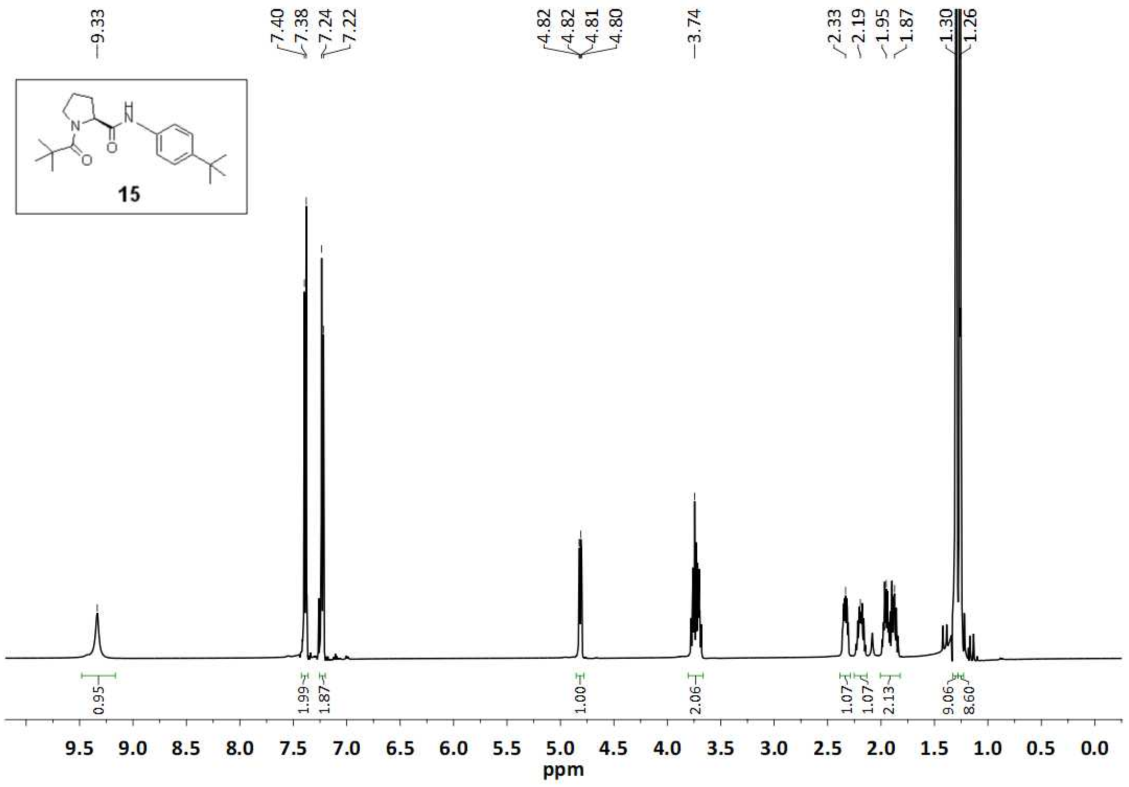

^{13}C-NMR (125 MHz, $CDCl_3$, rt)

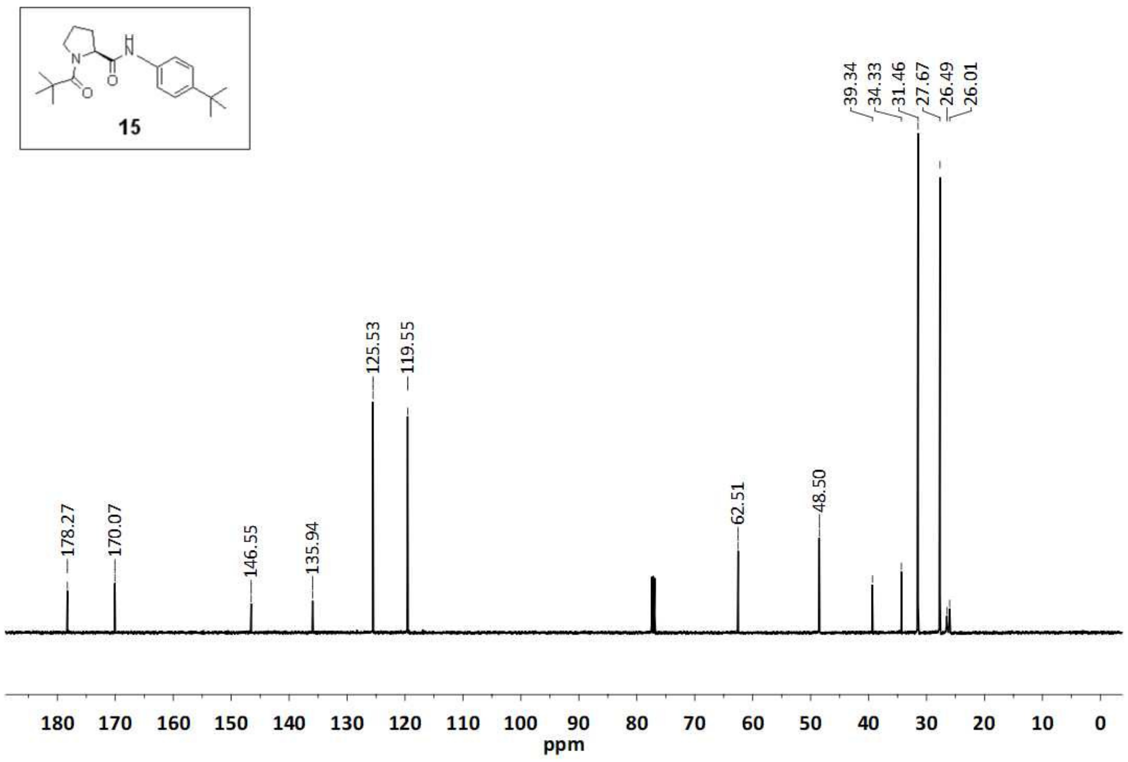
15
39.34
34.33
31.46
27.67
26.49
26.01
125.53
119.55
178.27
170.07
146.55
135.94
62.51
48.50
180 170 160 150 140 130 120 110 100 90 80 70 60 50 40 30 20 10 0
ppm

Catalyst 16

^{1}H-NMR (500 MHz, $CDCl_3$, rt)

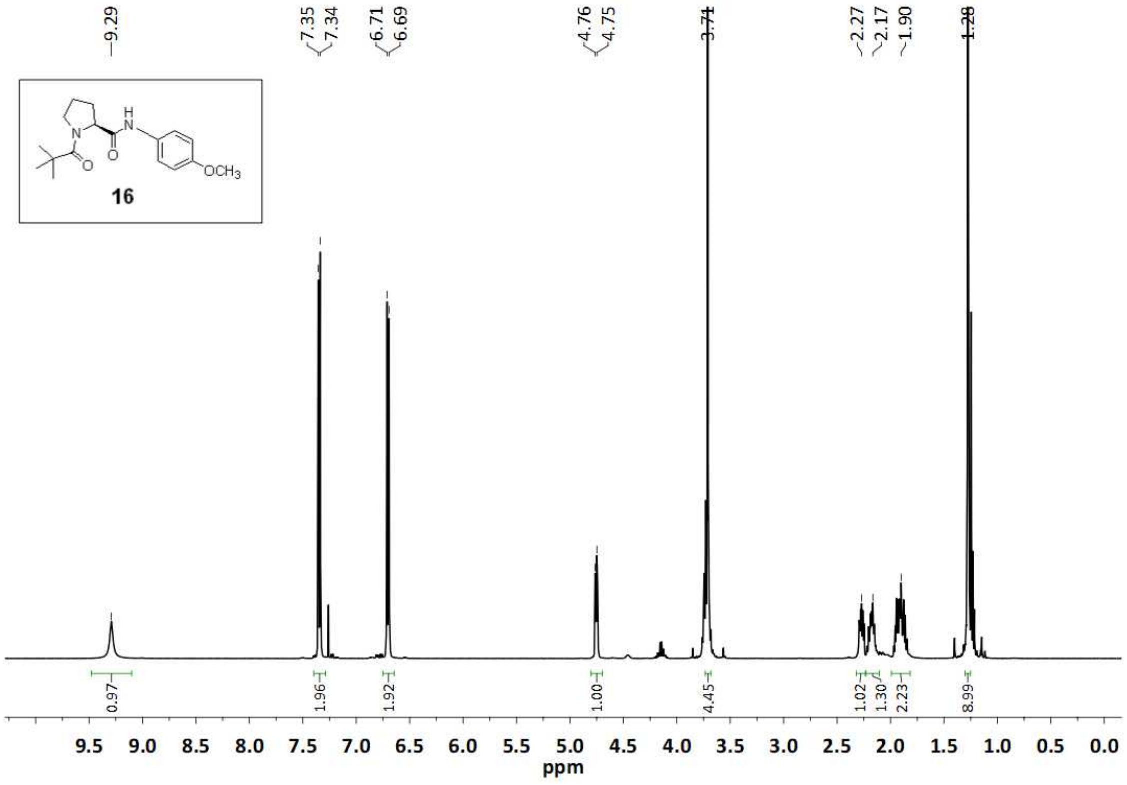

^{13}C-NMR (125 MHz, $CDCl_3$, rt)

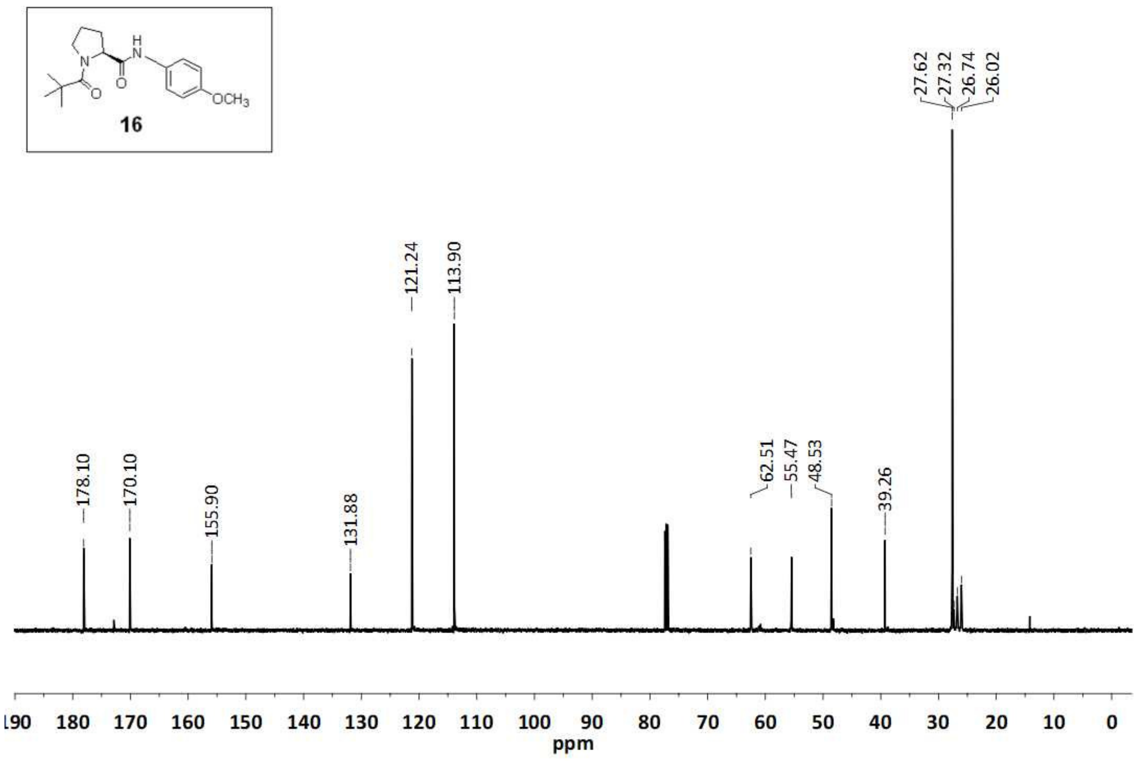
16
OCH3
178.10
170.10
155.90
131.88
121.24
113.90
62.51
55.47
48.53
39.26
27.62
27.32
26.74
26.02
ppm

5. References

[1] Chen, J.; Chen, X.; Zhang, Z.; Bao, Z.; Xing, H.; Yang, Q.; Ren, Q. MIL-101(Cr) as a synergistic catalyst for the reduction of imines with trichlorosilane. *Mol. Catal.* **2018**, *445*, 163−169.

[2] Gaussian 09, Revision B.01, M. J. Frisch, G. W. Trucks, H. B. Schlegel, G. E. Scuseria, M. A. Robb, J. R. Cheeseman, G. Scalmani, V. Barone, B. Mennucci, G. A. Petersson, H. Nakatsuji, M. Caricato, X. Li, H. P. Hratchian, A. F. Izmaylov, J. Bloino, G. Zheng, J. L. Sonnenberg, M. Hada, M. Ehara, K. Toyota, R. Fukuda, J. Hasegawa, M. Ishida, T. Nakajima, Y. Honda, O. Kitao, H. Nakai, T. Vreven, J. A. Montgomery, Jr., J. E. Peralta, F. Ogliaro, M. Bearpark, J. J. Heyd, E. Brothers, K. N. Kudin, V. N. Staroverov, T. Keith, R. Kobayashi, J. Normand, K. Raghavachari, A. Rendell, J. C. Burant, S. S. Iyengar, J. Tomasi, M. Cossi, N. Rega, J. M. Millam, M. Klene, J. E. Knox, J. B. Cross, V. Bakken, C. Adamo, J. Jaramillo, R. Gomperts, R. E. Stratmann, O. Yazyev, A. J. Austin, R. Cammi, C. Pomelli, J. W. Ochterski, R. L. Martin, K. Morokuma, V. G. Zakrzewski, G. A. Voth, P. Salvador, J. J. Dannenberg, S. Dapprich, A. D. Daniels, O. Farkas, J. B. Foresman, J. V. Ortiz, J. Cioslowski, and D. J. Fox, Gaussian, Inc., Wallingford CT, 2010.
